# Candida albicans Hyphal Extracellular Vesicles Are Different from Yeast Ones, Carrying an Active Proteasome Complex and Showing a Different Role in Host Immune Response

**DOI:** 10.1128/spectrum.00698-22

**Published:** 2022-05-23

**Authors:** Raquel Martínez-López, Maria Luisa Hernáez, Esther Redondo, Guillermo Calvo, Sonja Radau, Mercedes Pardo, Concha Gil, Lucía Monteoliva

**Affiliations:** a Department of Microbiology and Parasitology, Faculty of Pharmacy, Complutense University of Madrid (UCM), Madrid, Spain; b Ramon y Cajal Health Research Institute (IRYCIS), Madrid, Spain; c Proteomics Unit, Complutense University of Madrid, Madrid, Spain; d Thermo Fisher Scientific GmbH, Dreieich, Germany; e Functional Proteomics, The Institute of Cancer Research, London, United Kingdom; Septomics Research Center, Friedrich Schiller University and Leibniz Institute for Natural Product Research and Infection Biology—Hans Knöll Institute

**Keywords:** *Candida albicans*, proteomics, extracellular vesicles, yeast, hyphae, virulence factors, proteasome, cell wall maintenance, exosomes, macrophages, immunogenic

## Abstract

Candida albicans is the principal causative agent of lethal fungal infections, predominantly in immunocompromised hosts. Extracellular vesicles (EVs) have been described as crucial in the interaction of microorganisms with their host. Since the yeast-to-hypha transition is an important virulence trait with great impact in invasive candidiasis (IC), we have addressed the characterization of EVs secreted by hyphal cells (HEVs) from C. albicans, comparing them to yeast EVs (YEVs). YEVs comprised a larger population of bigger EVs with mainly cell wall proteins, while HEVs were smaller, in general, and had a much higher protein diversity. YEVs were able to rescue the sensitivity of a cell wall mutant against calcofluor white, presumably due to the larger amount of cell wall proteins they contained. On the other hand, HEVs also contained many cytoplasmic proteins related to protein metabolism and intracellular protein transport and the endosomal sorting complexes required for transport (ESCRT) pathway related to exosome biogenesis, pointing to an intracellular origin of HEVs. Interestingly, an active 20S proteasome complex was secreted exclusively in HEVs. Moreover, HEVs contained a greater number of virulence-related proteins. As for their immunogenic role, both types of EV presented immune reactivity with human sera from patients suffering invasive candidiasis; however, under our conditions, only HEVs showed a cytotoxic effect on human macrophages and could elicit the release of tumor necrosis factor alpha (TNF-α) by these macrophages.

**IMPORTANCE** This first analysis of HEVs of C. albicans has shown clear differences between them and the YEVs of C. albicans, showing their relevance and possible use in the discovery of new diagnostic markers and treatment targets against C. albicans infections. The data obtained point to different mechanisms of biogenesis of YEVs and HEVs, as well as different involvements in cell biology and host interaction. YEVs played a more relevant role in cell wall maintenance, while HEVs were more closely related to virulence, as they had greater effects on human immune cells. Importantly, an active 20S proteosome complex was described as a fungal-EV cargo. A deeper study of its role and those of many other proteins exclusively detected in HEVs and involved in different relevant biological processes of this fungus could open up interesting new areas of research in the battle against C. albicans.

## INTRODUCTION

Candida albicans can be found as a commensal fungus of humans, mainly on skin and mucosal surfaces, such as the oral cavity, gastrointestinal tract, and vagina. However, when host immunity is disrupted, C. albicans can cause an infection known as candidiasis, which can go from superficial candidiasis to life-threatening invasive candidiasis in immunosuppressed patients (1, 2). The C. albicans yeast-to-hypha transition is highly studied since it is critical for virulence. The hyphal morphology is generally considered to be more related to the invasion of host tissues, while the yeast morphology is more suited to bloodstream dissemination or surface commensalism (3). Proteomic studies of C. albicans dimorphism have used a variety of approaches, ranging from analyses of cytoplasmic and cell wall proteins from yeast cells, hyphae, and biofilms to quantitative analysis of the proteome during the yeast-to-hypha transition (4, 5). Different strategies have also been developed, such as the one described by Hernaez et al. based on “cell shaving” of live C. albicans cells (6, 7). This was applied to both yeast and hyphae and led to interesting findings, such as the identification of novel proteins involved in cell wall integrity, the yeast-to-hypha transition, and stress response and/or host-pathogen interactions (6, 7). Moreover, a similar strategy was used to decipher not only C. albicans proteins but also human serum proteins that were linked to the hyphal surface when yeast cells of C. albicans were incubated with serum, promoting their switch to hyphae (8).

Several proteins classically considered cytoplasmic because they lack signal peptides, including components of metabolic pathways, chaperones, and ribosomal proteins, have long been identified in proteomic studies as residing in the C. albicans cell wall or as part of the C. albicans secretome (9–13). Some secretory pathways that are alternatives to the endoplasmic reticulum (ER)-golgi apparatus for signal peptide-containing proteins have started to emerge (10, 11, 14). In addition, the existence of extracellular vesicles (EVs) in Gram-positive and Gram-negative bacteria and in fungi is being recognized (15, 16). Nowadays it is widely accepted that cells from almost every type of organism secrete these nano- to micrometer-scale lipid bilayer-delimited vesicles (15). A very detailed review on EVs secreted by different fungi has recently been published (17). In C. albicans, Anderson et al. demonstrated the existence of vesicle-like compartments in cell wall pimples from opaque cultures of C. albicans cells in 1990 (18). C. albicans EVs were first isolated and observed by transmission electron microscopy (TEM) in 2008 by Albuquerque et al., who demonstrated the presence of bilayered compartments similar to those initially described for Cryptococcus neoformans and Histoplasma capsulatum (19, 20). EVs of C. albicans yeast cells were later further analyzed to unravel their composition and implications for human immune responses in wild-type and mutant strains (9, 21–23). All this work has been extensively reviewed by Gil-Bona et al. (10).

Human EVs are well studied and have been classified as apoptotic bodies, microvesicles, or exosomes, depending upon their cellular origin and size (24). Apoptotic bodies are the largest (50 to 5,000 nm in size) and are derived from apoptotic cells. Microvesicles (100 to 1,000 nm) are generated by outward budding from the plasma membrane, followed by pinching off and release to the extracellular space. Exosomes are the smallest EVs (30 to 150 nm), and these structures originate from endosomal compartments (24–26). EVs shuttle bioactive molecules involved in many processes, including cell-cell communication, host-pathogen interactions, and even the sharing of microbial community resources in the case of microbial EVs. For example, Cryptococcus neoformans’ extracellular vesicles contain its major virulence factor, the capsular polysaccharide glucuronoxylomannan (27). EVs secreted by wild-type C. albicans biofilm are able to rescue the antifungal resistance of a defective biofilm produced by cells carrying mutations in genes encoding orthologues of endosomal sorting complexes required for transport (ESCRT) subunits (28). Other authors have proposed the use of these EVs as therapeutic carriers of drugs and metabolites, since the internalization of EVs secreted by different microorganisms and different mammalian cells has been widely proven in several recent research papers (29–31). The involvement of EVs from different microorganisms in the immunomodulatory response of the host has also been widely demonstrated (30, 32–36). Moreover, the use of these membranous structures as vaccination agents is the focus of many researchers (32, 37, 38).

In this context, and given the established contribution of EVs to key physiological aspects of cells from all kingdoms, we isolated and characterized EVs secreted by C. albicans cells of both major morphologies, yeast and hyphae, to better understand the mechanisms underlying the enhanced virulence associated with the morphologic transition. Differences in EV size and physical properties were analyzed by means of transmission electronic microscopy and dynamic light scattering (DLS). Protein cargoes were analyzed using liquid chromatography-mass spectrometry (LC-MS). The more interesting differences were observed in the proteomic analysis, suggesting that hyphal EVs (HEVs) differ in their biogenesis and function from yeast EVs (YEVs).

## RESULTS

We accomplished the study of YEVs and HEVs secreted by C. albicans cells using YNBS (5 g/L ammonium sulfate, 1.7 g/L yeast nitrogen base, 20 g/L sucrose) with either tartaric acid (pH 4) or a combination of MOPS (morpholinepropanesulfonic acid) and *N*-acetylglucosamine (N-AcGlc) (pH 7) to obtain yeast and hyphal morphologies, respectively. Prior to EV isolation, the morphological purity was assessed by microscopy, with around 99% pure yeast or hyphae observed. In addition, a viability assay with propidium iodide (PI) staining was conducted to verify the absence of cells with altered permeability. All samples showed a PI staining rate below 1% (Fig. S1 in the supplemental material).

### EVs secreted by hyphae are smaller than YEVs but carry a much more highly diverse protein cargo.

**FIG 1:**
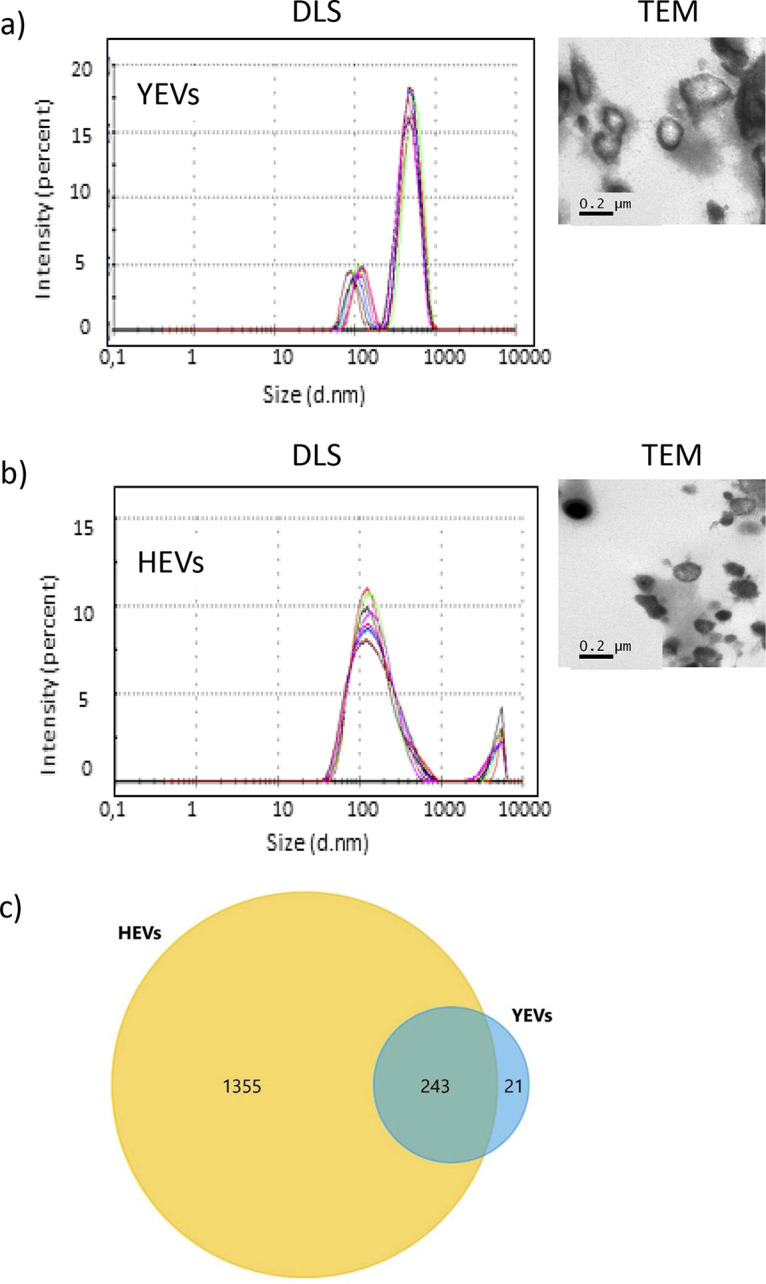
Differences in size and protein diversity between YEVs and HEVs. Size distribution by intensity pattern (DLS) and appearance (TEM) of YEVs (a) and HEVs (b). d.nm, diameter in nm. (c) Venn diagram showing the number of identified proteins that are in common or exclusive to EVs from each cell morphology.

YEVS and HEVs collected from culture supernatants were analyzed using DLS and TEM (Fig. 1a and b). Based on the size distribution, we observed that the majority of YEVs collected were significantly bigger than HEVs, most of them being in the range of 400 to 500 nm. However, there was also a small percentage of YEVs with a smaller size, more like the majority of HEVs, with a peak at 100 nm. HEVs presented a less homogeneous population, with the size distribution ranging from 50 to 450 nm, and there was a higher proportion of HEVs in the range of 100 to 200 nm. This difference in size could also be seen in the TEM analysis (Fig. 1a and b).

The protein cargoes of EVs from cells of each morphology were identified by a proteomic analysis. The LC-tandem MS (MS/MS) results showed a great difference in terms of the number of proteins identified, depending on the EVs’ origin (Fig. 1c). Taking into consideration only proteins that were identified in at least two biological replicates with at least two peptides in one of the replicates and a *q* value of <0.01 (Table S1, a and b), the numbers of proteins identified in HEVs and YEVs were 1,598 and 264, respectively, revealing the higher protein diversity of HEVs’ protein cargo.

### YEVs have a high proportion of cell surface-related proteins, which favors the growth of the *ecm33* mutant under cell wall-stressful conditions.

Of the 264 proteins identified in YEVs, 243 were also identified in HEVs (Fig. 1c).

**FIG 2:**
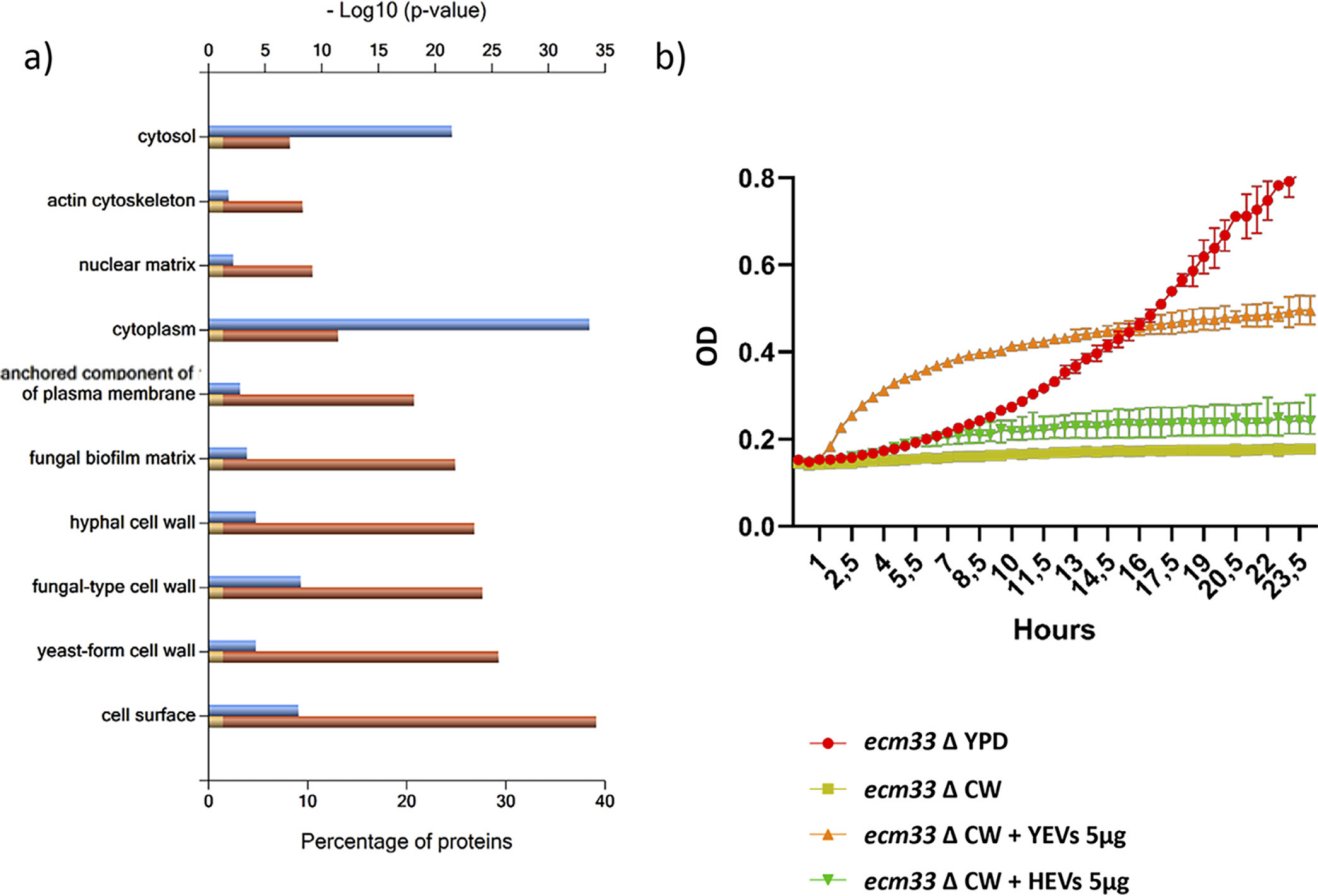
Proteins identified in both types of EVs are enriched in cell surface proteins and contribute to rescuing the *ecm33*Δ phenotype. (a) FunRich categorization of component enrichment of proteins identified in both YEVs and HEVs. The *P* value for significance is <0.001. (b) Rescue of the calcofluor white (CW) sensitivity exhibited by the *ecm33* mutant through the addition of YEVs and HEVs. The growth of the *ecm33* mutant was assayed in YPD and in YPD supplemented with 7 μg/mL of cell wall-disturbing agent CW in the absence or presence of 5 μg of either YEVs or HEVs. Error bars show standard deviations.

**FIG 3:**
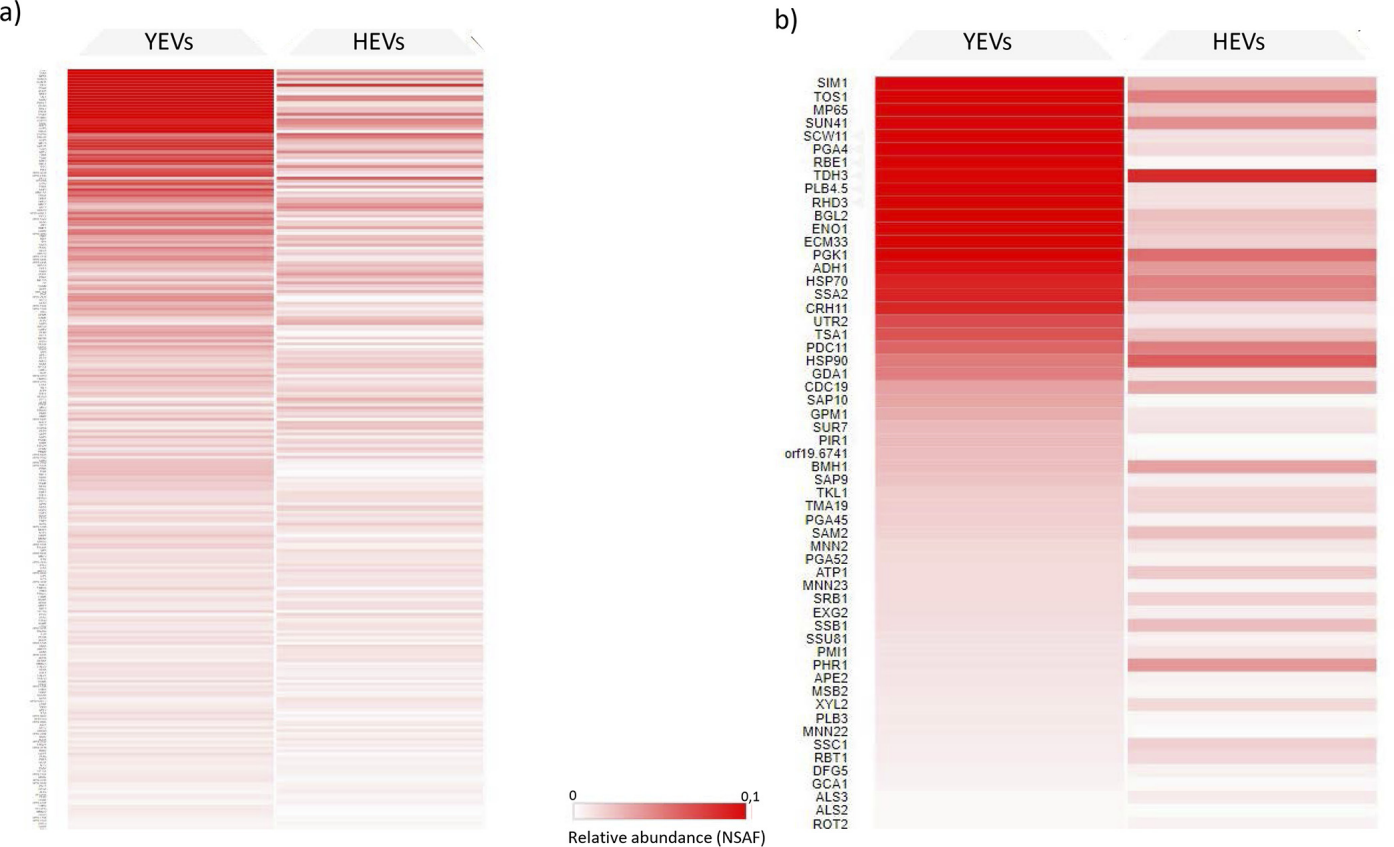
(a) Heat map of all proteins identified in YEVs based on their relative abundances (NSAF) in YEVs and HEVs. (b) Heat map of proteins from (a) described as cell surface related according to the CGD database. Proteins exclusively detected in YEVs show a value of zero for relative abundance in the HEV column.

Regarding the cell component category, and according to the Gene Ontology (GO) enrichment analysis from the Candida Genome Database (CGD), a high degree of enrichment in proteins from extracellular regions (including the cell surface, cell wall, or biofilm matrix) or anchored to the plasma membrane was clearly revealed. A FunRich analysis, which is based on a UniProt database and uses homologous proteins from all fungi, was in agreement with the CGD analysis (Fig. 2a). These proteins included typical cell wall proteins already described in numerous works, as well as cell surface-associated proteins, such as glycolytic enzymes (Eno1, Tdh3, and Pgk1). As shown by the data in Fig. 3, cell wall-related proteins had a higher relative abundance (normalized spectral abundance factor [NSAF] value) in YEVs than in HEVs.

Due to the abundance of cell periphery proteins in both types of EVs, we tested their effects on the growth of the C. albicans *ecm33* mutant, which lacks the glycosylphosphatidylinositol (GPI)-anchored cell wall protein Ecm33p and displays a defective cell wall and higher sensitivity to several cell wall-disturbing agents (39, 40), in a medium containing the cell wall-disturbing agent calcofluor white (CW). The addition of YEVs not only rescued completely the growth rate of the mutant, it enhanced it compared to the growth rate in YPD (1% d-glucose, 1% Difco yeast extract, and 2% agar) medium without CW (Fig. 2b). On the other hand, the addition of HEVs to the culture medium was able to minimally rescue the decreased growth rate of the *ecm33* mutant provoked by the CW. This difference could be due in part to the greater abundance of cell wall and periphery proteins within the YEVs’ protein cargo, as revealed by a higher NSAF value (Fig. 3).

### Only HEVs were enriched with proteins related to protein transport and protein metabolism, including an active 20S proteasome complex.

**FIG 4:**
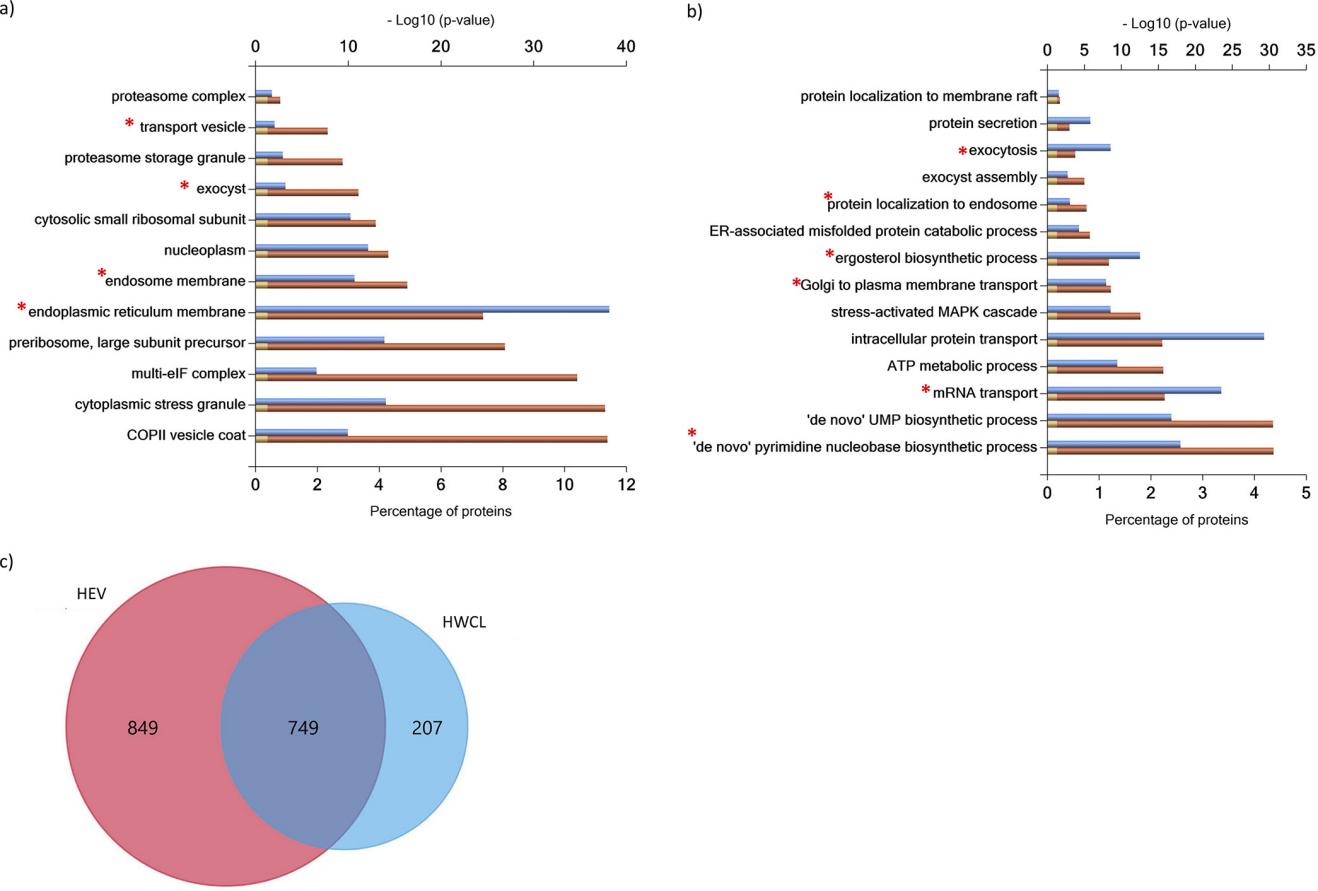
HEVs are enriched in proteins related to protein metabolism, transport, and biosynthetic pathways. FunRich categorization of component (a) and biological process (b) enrichment of proteins identified exclusively in HEVs and not in YEVs. Cellular components and biological processes marked with an asterisk (*) were enriched exclusively in HEVs and not in HWCL. (c) Venn diagram showing the number of identified proteins that are in common or exclusive to HEV and HWCL.

**FIG 5:**
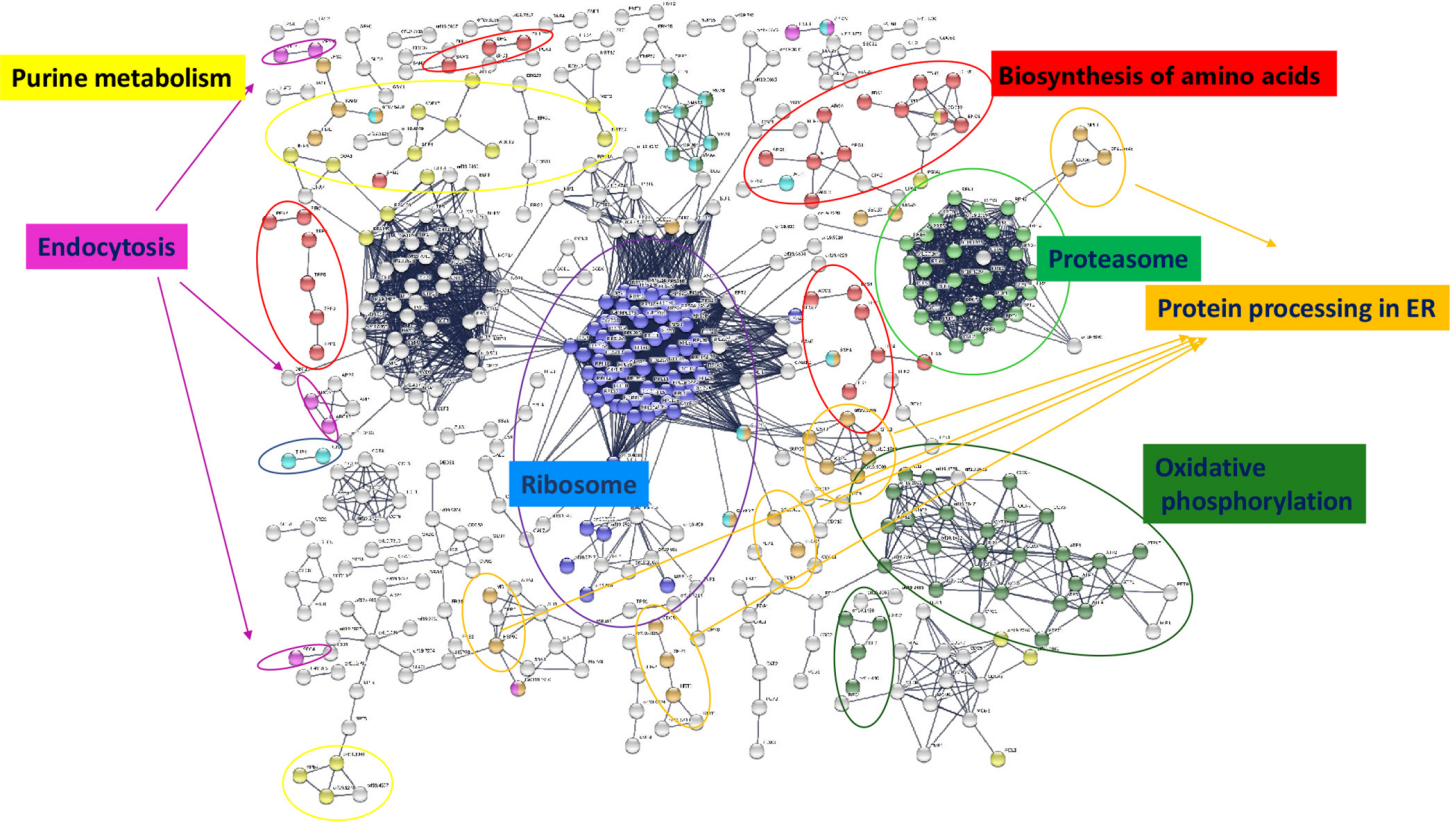
Protein-protein interaction network of proteins identified in HEVs using STRING software. Only nodes corresponding to proteins with the highest confidence (0.900) in active interaction sources of cooccurrence, coexpression, experiments, and neighborhood are shown.

In clear contrast to the enrichment in the extracellular component of proteins identified in both types of EVs or exclusively identified or enriched in YEVs, a GO enrichment analysis of the cellular component of the 1,355 proteins exclusively identified in HEVs revealed a predominantly cytoplasmic association, with remarkably high enrichment in GO categories related to protein metabolism (ribosome and the proteasome complex), protein transport (endocytosis and protein processing in the ER), and purine metabolism (Fig. 4a and b). Furthermore, when the proteins exclusively identified in HEVs were uploaded to STRING software, the relevance of these processes and others, such as oxidative phosphorylation and biosynthesis of amino acids also related to protein metabolism, was confirmed (Fig. 5).

Since we identified a much larger number of proteins in HEVs than in YEVs, we carried out a proteomic analysis of whole-cell lysates (WCLs) of cells of both morphologies in order to decipher whether this larger number of proteins in HEVs was merely due to a higher content of those proteins in the cytoplasm of hyphae. We identified up to 1,065 proteins in yeast WCL (YWCL) and 954 proteins in hyphal WCL (HWCL) (Table S2a and b). Interestingly, most proteins identified in the HEV cargoes were not identified in HWCL (Fig. 4c).

Only proteins identified in HEVs were enriched with proteins from the ER membrane, endosome membrane, transport vesicles, and exocyst (Fig. 4a), while the analysis of proteins identified in HWCL did not show enrichment in those components. Similarly, the enrichment of HEVs with enzymes from the *de novo* purine and pyrimidine biosynthetic pathways, the ergosterol biosynthetic process, and processes related to the maturation and transport of mRNA involved in translation were only observed in proteins identified in HEVs (Fig. 4b). These results make it very unlikely that the enrichment of HEVs with these proteins was a result of an artifact.

**FIG 6:**
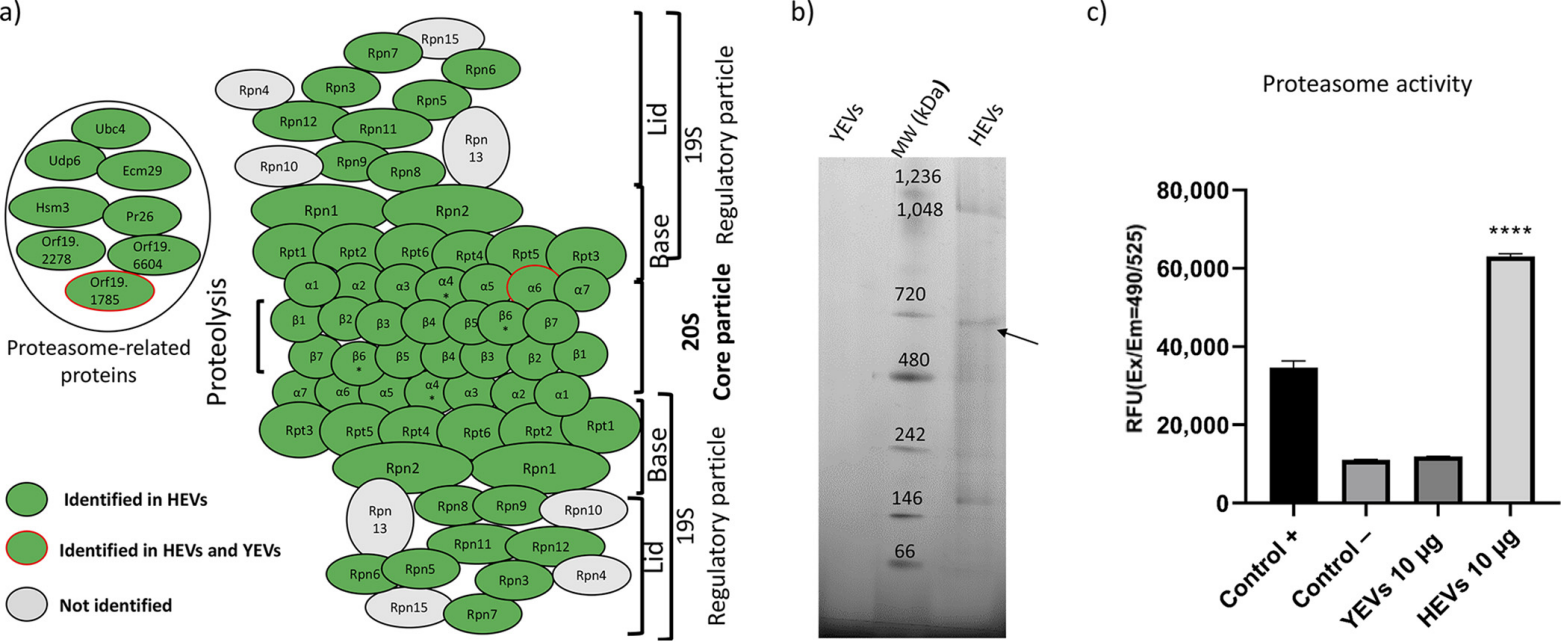
An active proteasome complex is only identified within HEVs’ protein cargo. (a) Schematic representation of the proteasome complex showing all the proteins from the 20S core particle and 19S regulatory particle. Proteins identified in HEVs are marked in green. *, proteins only identified in the proteasome complex from one-dimensional (1-D) blue native PAGE (BN-PAGE). α6 subunit, also identified in YEVs, is surrounded by a red line. (b) Separation of YEV and HEV complexes by 1-D BN-PAGE. The band corresponding to the proteasome complex is indicated by an arrow. (c) Validation of the chymotrypsin-protease activity of the proteasome through a fluorometric assay (in relative fluorescence units [RFU]). The chymotrypsin-protease activity of the proteasome from 100 μg of cytoplasmic extract was evaluated as a positive control. A significant change is indicated as follows: ****, *P* < 0.0001 (unpaired *t* test). Error bars show standard deviations.

Thus, we further analyzed the proteins involved in cellular processes that were exclusively enriched within the HEVs’ protein cargo from hyphal forms. Table 1 shows the proteins identified in YEVs and HEVs involved in these processes. In HEVs, regarding the proteasome structure, we identified 12 (92%) of the 14 proteins that form the 20S particle core and 16 (80%) of the 20 proteins that form the regulatory particle. Furthermore, two proteins (open reading frames [ORFs] Orf19.2278 and Orf19.6604) with a putative role in 20S proteasome assembly, two proteins (Ecm29 and Hsm3) that assist in the association of the proteasome core particle and regulatory particle, and four other proteins also related to the proteasome, Ubp6 (a putative ubiquitin-specific protease of the 26S proteasome), Ubc4 (with proteasome and ubiquitin binding activity), Pr26 (with similarity to the proteasomal 26S regulatory subunit of Saccharomyces cerevisiae), and Orf19.1785 (with a PI31 proteasome regulator domain), were also identified as part of the HEVs’ protein cargo (Fig. 6a). Since blue native PAGE (BN-PAGE) has been described as useful for the one-step isolation of protein complexes from biological samples (41), we carried out the separation of protein cargo from YEVs and HEVs by means of BN-PAGE to confirm the presence of the assembled complex in C. albicans HEVs (Fig. 6b). We could observe a protein band with a molecular weight of around 700 kDa (compatible with the molecular weight of the 20S core particle of the proteasome complex [42]) only in the HEV lane. Moreover, after the analysis of this band with LC-MS/MS, we confirmed the presence of all the subunits of the 20S core particle of the proteasome complex with high confidence and protein coverage, including the two subunits (Pre6 [α4] and Pre7 [β6]) that had not been included previously (Table 1) since they were only detected in one of the three biological replicates. The confirmation of the presence of the assembled 20S complex in HEVs led us to investigate whether its proteolytic activity was retained. A fluorometric assay based on the chymotrypsin-like protease activity associated with the proteasome complex was used. Interestingly, 10 μg of HEVs was able to surpass the chymotrypsin-like protease activity of 100 μg of C. albicans cytoplasmic extract that is recommended as the positive control in the manufacturer’s instructions. In contrast, the proteolytic activity of 10 μg of YEVs was comparable to that of the suggested negative control with phosphate-buffered saline (PBS) (Fig. 6c). Interestingly, this proteolytic activity, although higher with freshly isolated HEVs, was maintained over time for at least 2 weeks (data not shown).

**TABLE 1:** List of proteins identified in HEVs and YEVs related to biological processes that are enriched in HEV protein cargo and to virulence

Biological process	No. of proteins identified: name(s) or ORF(s)^*a*^			Total no. in:	
	In both YEVs and HEVs	Exclusively in:			
		YEVs	HEVs	YEVs	HEVs
Cellular processes					
Proteasome	2: Pre5 (α6), Orf19.1785		31: Scl1 (α1), Pre8 (α2), Pre9 (α3), Pup2 (α5), Prs1/Pre10 (α7), Pre3 (β1), Pup1 (β2), Pup3 (β3), Pre1 (β4), Pre2 (β5), Pre4 (β7), Rpt1, Rpt2, Rpt4, Rpt5, Rpt6, Rpn1, Rpn2, Rpn3, Rpn5, Rpn6, Rpn7, Rpn8, Rpn11, Ubp6, Ubc4, Ecm29, Hsm3, Orf19.2278, Orf19.6604, Pr26		33
Translation factors (initiation, elongation, and release)	4: Ded81, Anb1, Tif, Tef2^*b*^		13: Fun12, Sui2, Eif4e, Tif11, Tif5, Nip1, Prt1, Sui1, Sui3, Gcd2, Gcd11, Ria1,^*b*^ Erf1^*c*^	4	17
tRNA synthetases and ligases	1: Orf19.6701^*d*^		14: Grs, Dps1-1, Gln4, Mes1, Orf19.4931, Wrs1, Vas1, Tys1, Ths1, Hts1, Mes1, Frs1, Frs2, Gus1^*d*^	1	15
Ribosomal proteins	4: Asc1, Rpl12, Rpl14, Rpl10a		68: Rps18, Rps3, Rpl23a, Rpl6, Rps14b, Rpl3, Rpl20b, Rps8a, Rps26a, Rps27, Rpl10, Rps15, Rps5, Rps6a, Rps9b, Rpp0, Rps20, Rpl9b, Rpl4b, Rps22a, Rpl11, Rpl16a, Rpl19a, Rpl28, Rps24, Rpl38, Rpl24a, Rps17b, Rpl21a, Rps25b, Rps7a, Rpl15a, Rpl18, Rpl17b, Rpl13, Orf19.3341, Rps23a, Rpl5, Rpl7, Rps16a, Rps1, Orf19.4149.1, Rps13, Rpl27a, Orf19.2478.1, Rpl30, Rpl39, Rpl32, Rps19a, Orf19.3572.3, Orf19.3690.2, Rpl25, Rpl2, Rpl43a, Rpl35, Rps12, Rps21b, Rpl42, Rpp2a, Rps28b, Rpp2b, Rpl37b, Orf19.6220.4, Orf19.828, Orf19.512, Orf19.3778, Orf19.3559, Orf19.5698	4	72
Purine and pyrimidine biosynthesis	4: Ade13, Ade17, Ade 6, Ado1		17: Ade1, Ade2, Ade4, Ade5, Ade12, Imh3, Gua1, Cpa1, Cpa2, Ura1, Ura2, Ura3, Ura4, Ura5, Ura6, Ura7, Prs1	4	21
Amino acid biosynthesis	20: Hom6, Met6, Met15, Sah1, Sam2, Shm2, Cys3, Idp1, Idp2, Car2, His7, Asn1, Aro4, Aro8, Leu2, His1, Ser33, Arg1, Lys9, Ilv5	1: Hom2	46: Aro3, Aro9, Pro1, Pro2, Pro3, Lys1, Lys2, Lys4, Lys12, Lys21, Lys22, Arg3, Arg4, Arg5,6, Arg8, Trp2, Trp3, Trp4, Trp5 Cys4, Ser1, Ser2, His4, His5, His7, Tyr1, Prs1, Orf19.6306, Met2, Met3, Met10, Met13, Met14, Met16, Met18, Met13, Ilv1, Ilv2, Ilv3, Ilv6, Hom3, Sam51, Shm1, Leu1, Leu4, Leu42	21	66
Ergosterol biosynthesis	3: Erg10, Erg13, Erg20	2: Fmp45, Gcy1	11: Erg1, Erg3, Erg4, Erg5, Erg6, Erg9, Erg11, Ncp1, Erg26, Erg27, Hmg1	5	14
Required for resistance to toxic ergosterol analog	5: Car2, Dag7, Orf19.2047, Mnn23, Ypt31	1: Sap3	4: Amo2, Apl2, Nat2, Vid27	5	9
Induced by azole treatment or linked to azole resistance	52: Ach1, Aco1, Acs1, Adh1, Ado1, Ahp1, Ald5, Atp1, Cat1, Cht2, Dag7, Dak2, Ecm33, Eng1, Erg10, Ero1, Fba1, Fdh1, Fet34, Fma1, Gdh3, Glk1, Glx3, Gpm1, Grp2, Hhf1, Hsp70, Hsp90, Hxk2, Mcr1, Mid1, Mp65, Msi3, Orf19.1765, Orf19.1766, Orf19.7306, Pbr1, Pck1, Pdc11, Pet9, Pga52, Phr2, Plb3, Png2, Prx1, Pyc2, Rbt1, Rhd3, Sah1, Sur7, Tos1, Xyl2	7: Ade17, Als2, Bat22, Hom2, Orf19.4211, Pir1, Plb1	75: Asm3, Cdc3, Cka1, Cka2, Cmp1, Csh1, Cyb5, Ece1, Ecm331, Ena21, Erg11, Erg3, Erg4, Erg6, Erg9, Fas1, Fas2, Frp3, Fum12, Gal1, Gal10, Gal7, Glc3, Gph1, Gst2, Hgt7, Hsp21, Hym1, Hyr1, Ifd6, Ife2, Lsc1, Lys21, Lys22, Met13, Met3, Mir1, Mis11, Ncp1, Ole1, Op4, Orf19.1239, Orf19.2269, Orf19.2286, Orf19.2452, Orf19.2473, Orf19.3475, Orf19.3932, Orf19.4476, Orf19.6553, Orf19.6554, Orf19.7310, Orf19.7459, Orf19.851, Pda1, Pdb1, Pdr16, Pfk1, Pfk2, Plb5, Pma1, Por1, Rct1, Rnr21, Rpl35, Scs7, Sds24, Snf1, Snz1, Svf1, Tub2, Ucf1, Vma8, Zpr1	59	127
Virulence related					
Phospholipases	3: Plb4.5, Plb2, Plb3	1: Plb1	2: Plc2, Plb5	4	5
Sap proteins	4: Sap5, Sap7, Sap 9, Sap10.	1: Sap3	4: Sap2, Sap4, Sap6, Sap8	6	8
Als proteins	1: Als3	1: Als2	1: Als1	2	2
Proteins with a role in virulence according to CGD	12: Mnt1, Phr1, Het1, BglII, Rbt4, Rbt1, Asc1, Cdc42, Mnt2, Hex1, Kex2, Cat1	0	56: Hsp104, Nag1, Cdc3, Fas2, Vps21, Hxk1, Srv2, Rsr1, Mts1, Ras1, Tps1, Tps2, Alo1, Rvs161, Cdc10, Dac1, Arp2, Lap3, Kre5, Hsp21, Icl1, Ade5, Yhb1, Ade2, Gna1, Tpk2, Ftr1, Ssd1, Cka2, Mkc1, Orf19.3045, Ptc2, Met2, Nce103, Vtc4, Cmp1, Erg3, Nag6, Ura3, Slk19, Vps4, Orf19.3175, Och1, Ypt72, Csh3, Cek1, Pmt1, Pmt4, Bem1, Tcc1, Ssn6, Spf1, Pde2, Cla4, Mlt1, Spa2	12	68

a ORF, open reading frame.

b The protein is a translation elongation factor.

c The protein is a translation release factor.

d The protein is a tRNA synthetase or ligase.

We also identified up to 100 proteins related to ribosomes and the translation process in HEVs, including ribosomal proteins, translation initiation and elongation factors, and tRNA amino acid synthetases, but only 7 in YEVs (Table 1). The presence of these proteins, together with most of the proteins from the multi-eukaryotic initiation factor (eIF) complex, led to the identification of almost the entire 48S preinitiation complex, making translation one of the biological processes in which HEVs are significantly enriched (Fig. S2). Special attention should be given to the high number of proteins involved in the synthesis of different amino acids within the protein cargo of HEVs. In fact, the HEV protein cargo included all the enzymes necessary for the synthesis of valine, leucine, isoleucine, cysteine, serine, glycine, methionine, threonine, alanine, proline, lysine, tyrosine, and glutamine from fructose-6P. Moreover, HEV proteins also comprised all but one or two of the enzymes necessary for the biosynthesis of histidine, tryptophan, phenylalanine, and asparagine (Table 1).

Interestingly, only HEVs were enriched in proteins belonging to the oxidative phosphorylation route containing many of the F- and V-type ATPase subunits (Fig. S3), contributing to making ATP metabolic processes one of the more significantly and exclusively enriched processes in HEVs (Fig. S2). Also worth mentioning is the high significance of purine and pyrimidine biosynthetic processes among the biological processes represented by proteins exclusively identified in HEVs. All of the purine metabolic enzymes in the route from phosphoribosyl pyrophosphate (PRPP) to IMP and then to AMP were identified in HEVs, while only three were identified in YEVs (Table 1). Therefore, it is not surprising that HEVs were enriched in molecular functions like ATP binding, adenylate kinase activity, and AMP binding (Fig. S2). It is also remarkable that only HEVs were highly enriched with proteins that belong to cellular components of the classical protein secretion pathway, such as the ER, Golgi complex, or COPII vesicle coatings, or other components involved in vesicular traffic, such as components of the ESCRT-0 complex, the endosome membrane, or the exocyst (Table 2).

**TABLE 2:**
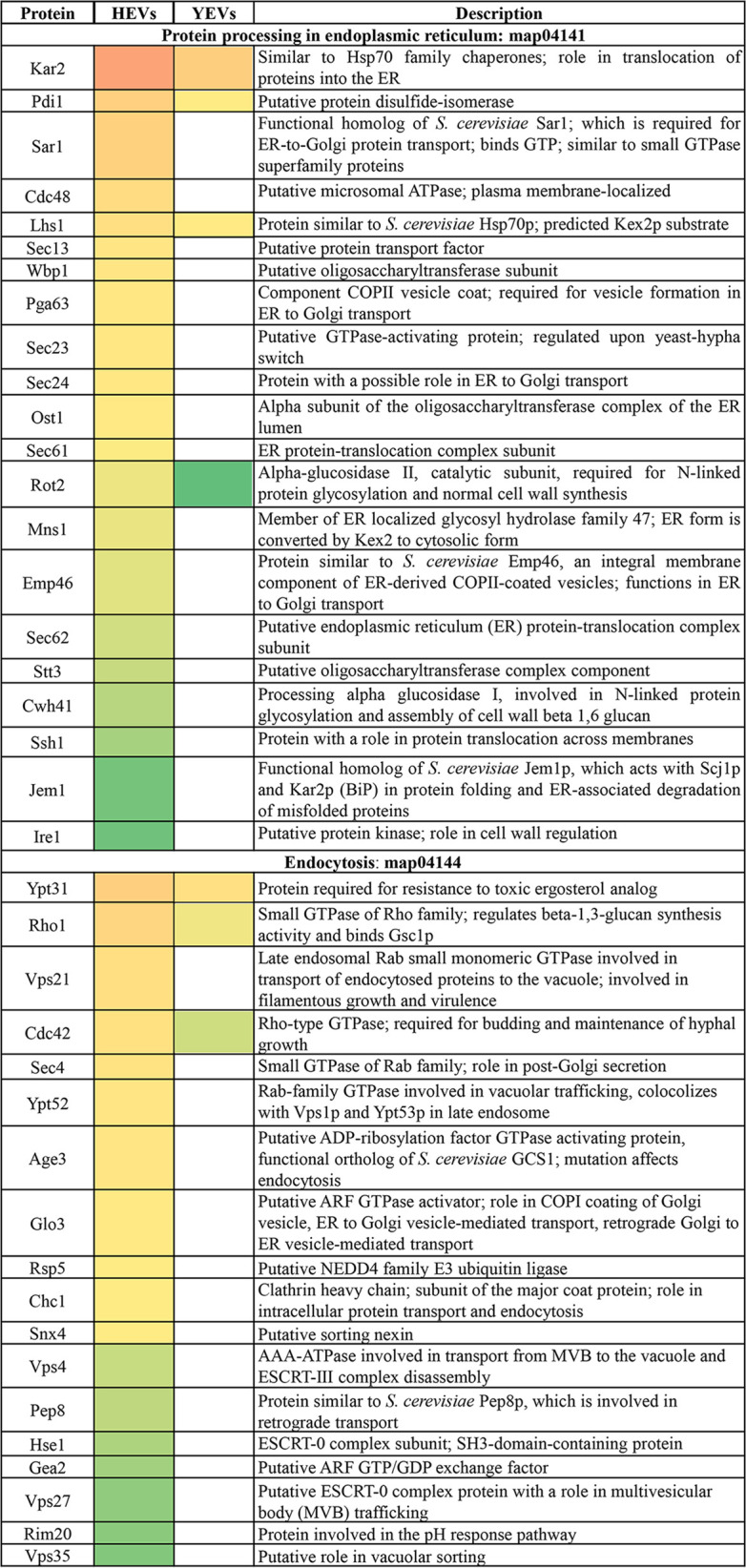
List of proteins identified in HEVs and related to pathways and structures involved in vesicular transport^*a*^

a Categorization is according to the CGD. Conditional coloring is applied according to the relative abundance of the protein (NSAF) within HEVs (red is the most abundant and green is the least). If the protein has also been identified in YEVs, its relative abundance within YEVs is also shown, while if such a protein has not been identified in YEVs, the YEV column is blank.

It is also interesting to note the enrichment of HEVs with proteins from the ergosterol biosynthetic pathway (Table 1), as ergosterol is the main target of azoles, a fundamental group of widely used antifungals. Furthermore, 127 of the proteins identified in HEVs have been described as either induced by azole treatment or linked to azole resistance (Table 1).

### Differences in virulence factors and interaction with the immune system between YEVs and HEVs.

Lipases, phospholipases (PLBs), and secreted aspartic proteases (Saps) are classical virulence factors secreted by C. albicans. We identified several proteins with phospholipase and protease activity in both types of EV but differences in the number and type depending on the EVs’ origin. Agglutinin-like sequence (Als) proteins were also differentially identified regarding the EVs’ origin (Table 1). Not surprisingly, HEVs contained a greater number of proteins related to C. albicans virulence (Table 1).

An interesting result regarding virulence factors is the identification of Ece1p protein in HEVs but not in YEVs. Ece1 proteolytic processing and maturation by Kex1 and Kex2 proteins yields the C. albicans toxin candidalysin. Ece1p and both Kex proteins were identified in the protein cargo of HEVs, but the peptide corresponding to candidalysin was not.

**FIG 7:**
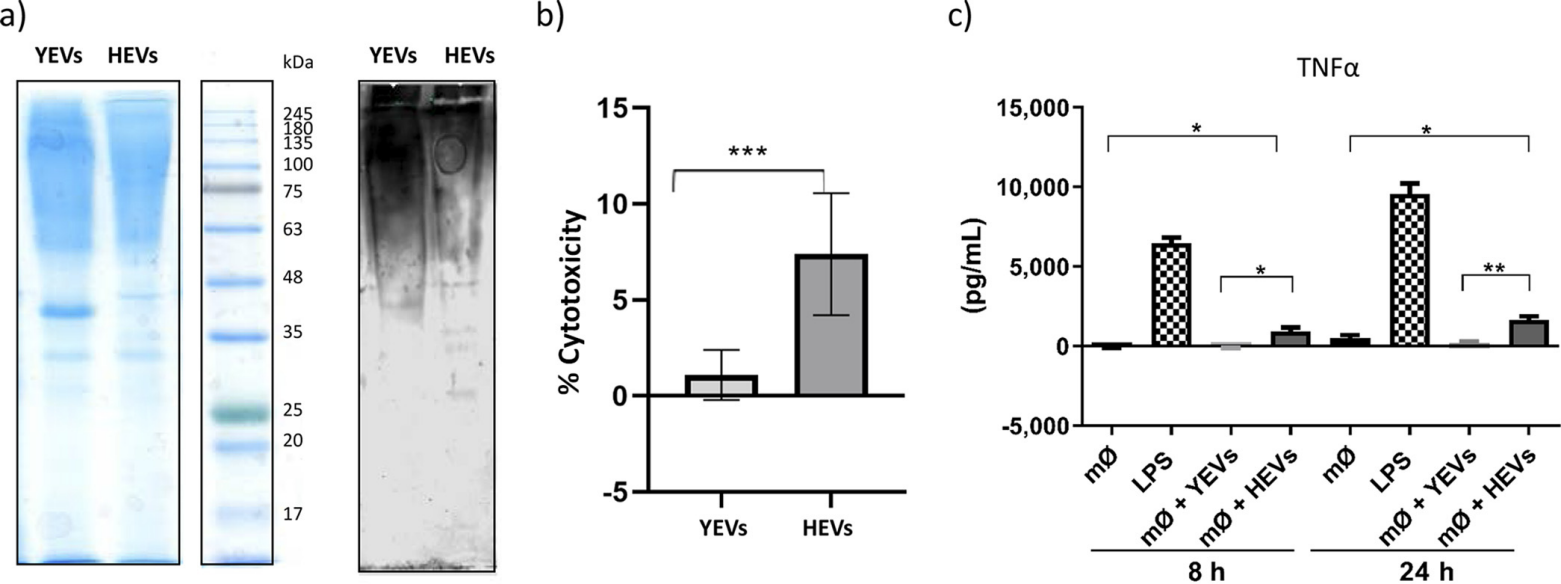
Differences in the immune response against YEVs and HEVs. (a) SDS-PAGE Coomassie-blue stained gel and Western blot showing the immunoreactive patterns of YEV and HEV protein extracts to sera from patients suffering invasive candidiasis. (b) Percentage of cytotoxicity produced by either HEVs or YEVs to TPH1 macrophages. (c) TNF-α release from TPH1-activated macrophages (mØ) incubated for 8 or 24 h with 5 μg of YEVs or HEVs. Negative and positive controls (with the addition of PBS and LPS, respectively) were also evaluated. A significant change is indicated as follows: *, *P* < 0.05; **, *P* < 0.01; ***, *P* < 0.001 (unpaired *t* test). Error bars show standard deviations.

Since both types of EVs contained many proteins related to the cell wall and cellular surface (Fig. 2a and 3), with many of them being reported as immunogenic, we tested the YEV and HEV protein cargoes for reactivity with human sera from invasive candidiasis patients. We had previously observed that protein cargoes from both types of EV displayed different electrophoretic patterns but contained high-molecular-weight proteins that were likely to correspond to highly glycosylated cell wall proteins (Fig. 7a). Moreover, even though sera from patients with invasive candidiasis were able to recognize proteins from both types of EV, high-molecular-weight proteins were recognized as having a stronger signal in YEVs (Fig. 7a). In contrast, other proteins with lower molecular weights were exclusively detected in HEVs.

Since the proteomic study rendered results showing large differences in terms of the diversity of the protein cargoes (HEVs containing many more virulence-related proteins), we wanted to validate such differences by testing the influence of each type of C. albicans EV on THP1 human macrophages. For this, we first tested their cytotoxic effects *in vitro* on this cell line by incubating the THP1 cells with 5 μg of either HEVs or YEVs for a period of 8 h and measuring the lactate dehydrogenase (LDH) activity (an internal enzyme that increases its abundance in culture supernatant upon cell damage) from the culture supernatant. The increase in the LDH activity of the culture supernatants from the THP-1 macrophages incubated with HEVs compared to the LDH activity of supernatants from the control macrophages was around 7%, while for the incubation with YEVs, this increase was not substantial. Therefore, the cytotoxic effects on THP-1 macrophages were significantly different in HEVs and YEVs, supporting the disparity in the contents of the two types of EVs (Fig. 7b).

Regarding the ability to stimulate the secretion of cytokines by THP-1-derived macrophages, only HEVs were able to increase the secretion of tumor necrosis factor alpha (TNF-α) at the two time points assayed, increasing the amount of this released cytokine at 24 h (Fig. 7c). No significant differences were observed in the case of the secretion of other cytokines, such as interleukin 10 (IL-10) and IL-12.

## DISCUSSION

To the best of our knowledge, this is the first work to analyze the EVs secreted from C. albicans during filamentous growth, comparing them to the EVs secreted by yeast cells.

Different sizes of EVs have been described in different works, and both the strain and culture conditions seem to contribute to the wide heterogeneity in sizes, protein cargoes, and biogenesis of EVs (10). Under our culture conditions, HEVs were in general smaller than YEVs, with a larger population of vesicles in the size range of 100 to 200 nm, in contrast to 400 to 500 nm for YEVs. In accordance with our results, an analysis of EVs secreted by biofilm and yeasts from the reference C. albicans strain DAY286 revealed that the EVs secreted by biofilms were also of smaller size (43).

The differences in the protein cargoes were also very relevant with respect to protein diversity. We identified 6-fold-more different proteins in HEVs than in YEVs, analyzing the same amount of protein from each. Ninety-two percent of the proteins identified in YEVs were also detected in HEVs, but in the latter EVs, these proteins only represented 16% of their protein cargo. A disparity in the number of proteins identified depending on cell morphology has also been observed in other C. albicans studies of extracellular proteins, e.g., Luo et al. identified 4-fold more proteins in the hyphal than in the yeast secretome (44). Similarly, in a study of C. albicans cell surface proteins (the surfome), Gil-Bona and coworkers described around 400 and almost 900 proteins in yeast and hyphal cells, respectively (7). All these data are evidence that the extracellular environment has higher protein diversity in hyphae than in yeast cells. Moreover, we confirmed that this higher protein diversity is not randomly due to a higher content of these proteins in the hyphal cell, since many of the proteins identified in HEVs were not identified in the proteomic analysis of HWCL. It is plausible that this higher protein diversity contributes to hyphal adhesion and tissue invasion.

On the other hand, we observed that YEVs but not HEVs rescued the growth rate of the *ecm33* cell wall mutant in CW-containing medium and, in fact, the growth rate of the mutant under these conditions was even better than that of the mutant growing in YPD medium without CW. This is in line with a recent work from Zhao and colleagues that describes a potential role of S. cerevisiae EVs in cell wall remodeling (29). We hypothesize that the abundance of cell wall proteins within YEVs would be the main reason for the reduction of the sensitivity of the cell wall mutant *ecm33* to CW.

### Only HEVs contain an active 20S proteasome and other proteins that could be relevant for the survival of the fungus.

One of the most important results of this work is the identification of an active 20S proteasome within the HEVs’ protein cargo. We demonstrated that all of the proteins of the 20S proteasome were assembled, since we were able to separate out the corresponding protein complex with a molecular weight of around 700 kDa in a native PAGE gel. Although the presence of different proteins from this complex has also been reported in EVs secreted by other microorganisms, such as Acanthamoeba castellani, and in other fungal EVs, including those of other strains of C. albicans (collected in the ExVe database) (20, 45, 46), an active 20S proteasome complex had not been observed in the EVs secreted by fungal cells so far.

This makes our result more interesting, since we have confirmed high proteolytic activity associated with the proteasome in C. albicans HEVs. Regarding the EVs secreted by human cells, the presence of proteins belonging to the human proteasome complex has been reported multiple times (see Vesiclepedia [www.microvesicles.org]). In some cases, the 20S proteasome complex contained in EVs was confirmed to be inactive, as in the study reported by Yunusova et al., regarding the plasma exosomes of patients with breast and ovarian tumors (47). More recently, the presence of an active 20S proteasome in the EVs secreted by platelets that has been involved in protein processing for antigen presentation via major histocompatibility complex I (MHC-I) molecules has been described (48). The presence of an active 20S proteasome in the EVs secreted by red blood cells (RBCs) infected with Plasmodium falciparum parasites has been proven to modify the cell membrane of naive RBCs, favoring the entrance of new parasites into them (49). Moreover, proteasome inhibitors negatively impact cell survival and proliferation processes, becoming an attractive niche for the treatment of cancer and inflammatory diseases (50, 51). All of these studies show the significant biological importance of this complex.

Furthermore, our group analyzed the proteomic response of C. albicans to 10 mM hydrogen peroxide (H_2_O_2_) and observed an increase in the abundance of different proteasomal proteins from the catalytic subunit and in the proteolytic activity associated with proteasome in the cytoplasmic extract (52). Since C. albicans faces oxidative conditions in its battle against the human immune system, it could be interesting to further investigate whether the release of large amounts of this complex within HEVs could somehow benefit the survival of the fungus against the immune system.

On the other hand, the enrichment of HEVs in proteins related to protein synthesis, including proteins from ribosomes, the translation initiation complex, and amino acid biosynthetic pathways, suggests that HEVs are somehow involved in C. albicans protein metabolism.

Also remarkable is the identification in HEVs of many enzymes involved in the biosynthesis of purines. Purines are essential molecules in DNA and RNA backbones, energy utilization, the regulation of enzyme activity, and cell signaling. Hence, it is not strange that all the enzymes needed to synthesize this valuable resource are packaged in EVs to be secreted and shared by all Candida cells in the community (28). Moreover, HEVs also contain a higher number of proteins from ergosterol biosynthesis and many proteins described in other works as required for resistance to toxic ergosterol analogues, induced by azole treatment or linked to azole resistance, which could counteract, to some extent, the effect of certain antifungals, such as azoles, one of the main treatments for fungal infections.

### C. albicans EVs contain virulence factors and influence the host immune response.

Ece1p is the candidalysin preproprotein, a fungal peptide toxin critical for mucosal infection (53, 54). After sequential proteolytic processing by Kex2 and Kex1, candidalysin is secreted and can be detected in culture supernatants and during growth on epithelial cells (53, 55, 56). We identified 13 different peptides from Ece1p covering 63% of the protein sequence, as well as both Kex2 and Kex1, exclusively in HEVs, though we did not identify the peptide corresponding to candidalysin. This does not necessarily support the lack of the toxin in HEVs, because it could also be due to difficulties in its ionization or detection. More experiments are needed to decipher the real presence of candidalysin in HEVs, which would represent an alternative mode of Ece1 processing and secretion.

Other virulence factors secreted by C. albicans, such as Sap, Als, and PLB proteins, were also identified in both types of vesicles, although with marked differences in the type and abundance depending on the morphology of origin and in agreement with previous proteomic studies of the secretome from both morphologies (57). The presence in HEVs of Sap4, -5, and -6 (reportedly essential for C. albicans systemic invasion [58]) and Als3 (which has been directly linked to adhesion to and invasion of human cells [59]) could be one of the reasons behind the cytotoxic damage displayed by THP-1 macrophages after 8 h of incubation with HEVs. Sap7, Sap9, and Sap10 showed higher relative abundances in YEVs than in HEVs (Fig. 3). According to Albrecht et al., Sap9 and Sap10 are retained at the cell surface via a modified GPI anchor (60). In fact, the higher abundance of these GPI-linked Saps and other cell surface-related proteins within the YEV cargo agrees with the hypothesis, proposed by Gil-Bona et al. (21), of YEVs’ main origin in outward budding from the plasma membrane. Moreover, the abundance of cell surface-related proteins within YEVs was able to enhance the slower growth of the defective cell wall mutant *ecm33* in a medium containing CW (Fig. 2b).

**FIG 8:**
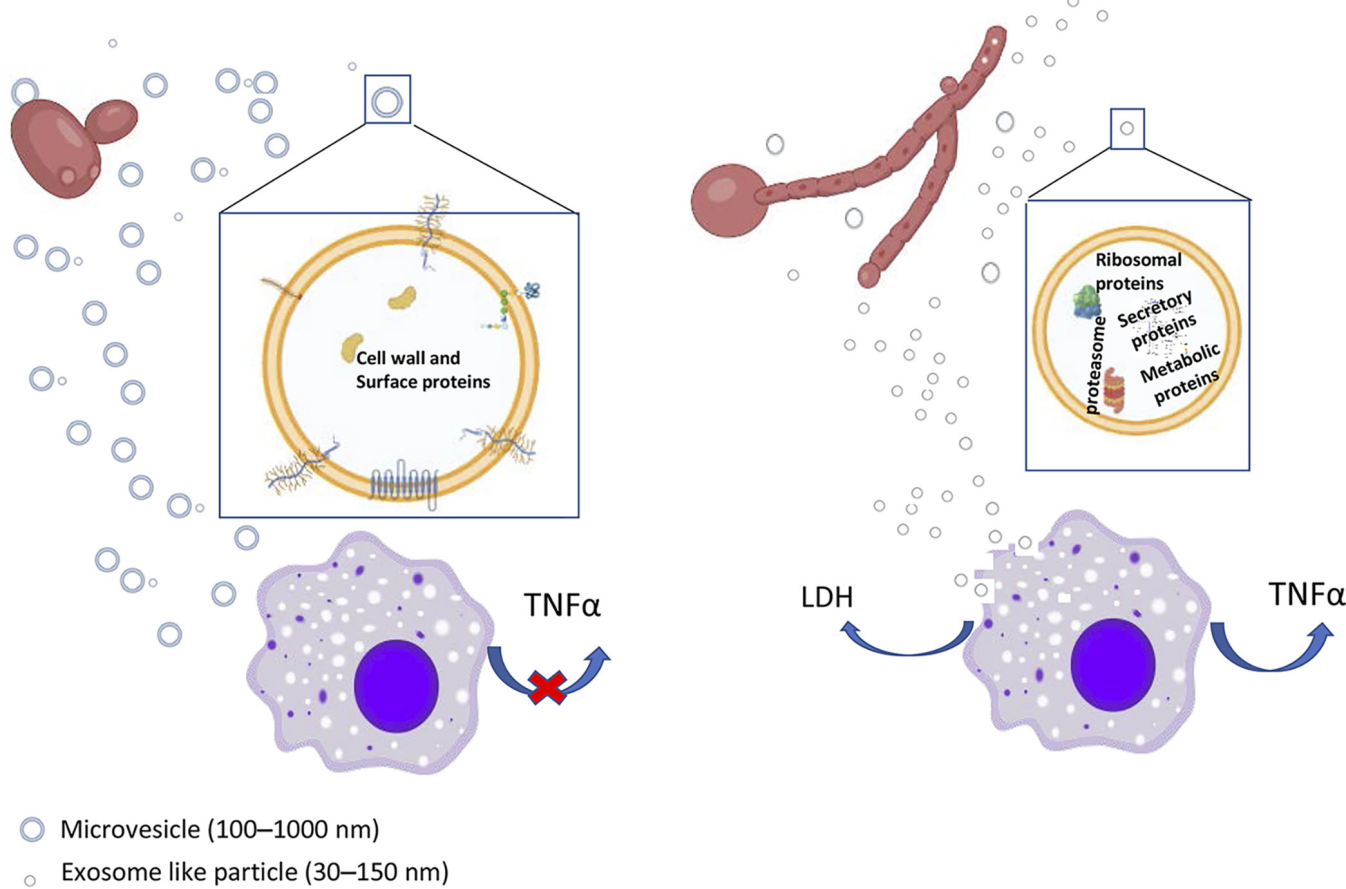
Schematic representation of the main differences observed between HEVs and YEVs regarding their protein cargoes and interactions with THP1 macrophages. An enlarged view of an HEV and a YEV showing the main component-related protein enrichment of each type of EV is also presented.

On the other hand, the immunomodulatory effects of EVs secreted by different organisms, including C. albicans, have already been described (17, 22, 30). Cell surface and secreted proteins, as well as proteins of YEVs, have also been proven to be immunogenic in many works (61–65). Moreover, the presence in both types of EVs of many immunogenic proteins was demonstrated using Western blots probed with sera from a patient suffering invasive candidiasis (Fig. 7a). However, it is worth pointing out that the signal corresponding to highly glycosylated proteins, which are expected to mainly be cell surface proteins, was stronger in YEVs, according to the proteomic data. On the other hand, the larger number of virulence-related proteins in HEVs could be responsible for the higher cytotoxic effect displayed by HEVs (Fig. 8).

Regarding cytokine release by THP-1 macrophages, under the conditions tested, only HEVs were able to enhance the induction of TNF-α when incubated with THP-1 macrophages (Fig. 8). Even though the release of TNF-α by bone marrow-derived murine macrophages stimulated by C. albicans YEVs has been described, it is worth mentioning that the strain of C. albicans used in that study was different from SC5314 and that the EVs were also obtained under different culture conditions, which greatly alters the composition of the vesicles, as has been suggested in several studies (22).

### C. albicans HEV and YEV cargoes point to different mechanisms of biogenesis.

Apart from the cell surface proteins also contained in YEVs, HEVs contain numerous cytoplasmic proteins. In fact, the 100 most abundant HEV proteins were cytoplasmic, in contrast to YEVs, in which the 100 most abundant proteins were cell surface related. This piece of evidence, together with the existence of a small proportion of larger vesicles secreted by hyphae (more similar in size to YEVs), suggests that hyphae might produce two different types of EV: bigger HEVs whose protein cargoes are probably cell surface- and membrane-related proteins commonly identified in YEVS and a larger proportion of smaller HEVs enriched in cytoplasmic proteins (Fig. 8), with the two types produced by different mechanisms. EV size has been one of the most widely used criteria for vesicle classification, with small (<150 nm) vesicles being classified as exosomes of endolysosomal origin, while larger vesicles (100 to >1,000 nm) are classified as microvesicles (66–68). Components from ESCRT machinery that have been linked to exosome biogenesis have been identified in exosomes in many proteomic studies (see Vesiclepedia [www.microvesicles.org]). In C. albicans, ESCRT pathway-related mutants were deficient in vesicles secreted from biofilms (a morphology intrinsically related to hyphal forms) (28).

In line with these facts and based on the generally larger size of a considerable number of YEVs and their preferential enrichment in cell wall and cytoplasmic membrane proteins (Fig. 8), these bigger vesicles are more likely to be microvesicles that budded, pinched off, and were released to the extracellular space from the plasma membrane, as previously proposed by other authors (21). This is probably also the origin of the smaller proportion of larger HEVs. In contrast, the contents of the larger proportion of smaller HEVs, including ESCRT components and many proteins from different endomembrane compartments, such as the ER, COPII vesicles, endosomes, multivesicular bodies (MVBs), and vacuole, suggest an origin of HEVs in these intracellular protein-trafficking regions. For instance, proteins related to MVB formation have been exclusively identified in HEVs, as have proteins belonging to the ESCRT pathway (Vps4 from ESCRT-III and Hse1 and Vps27 from ESCRT-0) (Table 2). In fact, the study on C. albicans EVs secreted by biofilms, highlighted the relevance of this ESCRT pathway in the secretion of EVs, since most ESCRT-defective mutations caused reduced biofilm EV production, reduced matrix polysaccharide levels, and greatly increased sensitivity to the antifungal drug fluconazole (28). Furthermore, the protein composition of EVs secreted by ESCRT pathway-related mutants was seen to be significantly different from the protein composition of those secreted by wild-type cells (69). Moreover, turbinmicin, an antifungal with proven efficacy in disrupting C. albicans biofilm growth, has been reported to exert its antifungal effect, at least in part, though the inhibition of vesicle trafficking (70). All of these data are in accordance with our hypothesis that, unlike YEVs, which seem to be budded and pinched off from the plasma membrane, the smaller HEVs would have an intracellular origin related to the ESCRT pathway.

## MATERIALS AND METHODS

### Fungal strains and culture conditions.

The C. albicans clinical isolate SC5314 (71) and the cell wall *ecm33* mutant strain RML2U (*ecm33*Δ::*hisG*/*ecm33*Δ::*hisG ura3Δ*::*imm434*/*ura3Δ*::*imm434*::*URA3*) (39, 40) were used in this work. EVs were obtained from strain SC5314. It was grown on YPD agar plates (1% d-glucose, 1% Difco yeast extract, and 2% agar) overnight at 30°C prior to the experiment. Two isolated colonies were used to inoculate 200 mL of liquid SD medium (20 g/L glucose, 5 g/L ammonium sulfate, 1.7 g/L yeast nitrogen base, 1.92 g/L synthetic amino acid mixture minus uracil Formedium supplemented with 0.1 g/L uracil). C. albicans cultures were grown for 6 h at 30°C and 180 rpm. Cells were then collected by 10 min of centrifugation at 2,500 rpm in an Eppendorf 5810R centrifuge, the supernatant discarded, and cells washed with 1 mL of phosphate-buffered saline (PBS) and collected again by 3 min of centrifugation at 5,000 rpm in a Heraeus Fresco 21 microcentrifuge (Thermo Scientific). Cells were then counted in a Neubauer chamber to inoculate 10^6^ cells into 1-L volumes of the different media used to obtain the specific morphologies.

For yeast morphology, 1 L of YNBS (5 g/L ammonium sulfate, 1.7 g/L yeast nitrogen base, 20 g/L sucrose) was supplemented with 75 mM tartaric acid adjusted to pH 4.

For hyphal morphology, 1 L of YNBS was supplemented with 75 mM MOPS (morpholinepropanesulfonic acid) adjusted to pH 7.4 and 5 mM *N*-acetylglucosamine (N-AcGlc) (44, 57).

Cell wall mutant strain RML2U was maintained in YPD agar plates. The culture conditions used to calculate its growth rates in YPD and YPD supplemented with 7 μg/mL of calcofluor white are described next.

### Complementation of calcofluor white sensitivity assay.

The C. albicans *ecm33* mutant (lacking the GPI-anchored cell wall protein Ecm33p) (39) was used to test the ability of YEVs and HEVs to complement the sensitivity of this cell wall mutant to the cell wall-disturbing agent calcofluor white. For this, the growth curve of the *ecm33* mutant in YPD medium supplemented with 7 μg/mL calcofluor white was measured in the presence of 5 μg of either YEVs or HEVs. As controls, measurements of the growth rates of the *ecm33* mutant in YPD medium and YPD medium supplemented with 7 μg/mL calcofluor white were also performed. All growth curve experiments were carried out simultaneously in 96-well Nunc plates with the same *ecm33* inoculum of 10^4^ cells in 180 μL of medium. Dissolved oxygen (DO) measurements were taken every 30 min using a SPECTROstar Nano (BMG Labtech). Each growth curve experiment was performed in triplicate with 3 different biological replicates.

### C. albicans cell viability measurement.

Prior to isolation of EVs, 1-mL amounts of yeast and hypha culture media were treated with propidium iodide (PI) to test cell viability. PI is nonpermeable to intact cell membranes but can enter dead cells with compromised membranes and dye DNA molecules. Amounts of 10^6^ cells were incubated with 10 μL of 5 mM PI (Fluka), and the fluorescent cells were counted under a fluorescence microscope at λ = 450 nm. Cells treated with 70% ethanol–PBS were used as the positive control for dead cells. At least 500 cells of each sample were counted to calculate the percentage of nonviable PI-stained cells.

### Isolation of extracellular vesicles.

Three independent experiments were done with yeast and hyphal cultures. The isolation of EVs was done according to Gil-Bona et al. (21). The whole process was conducted at 4°C. In brief, the supernatants from 1-L volumes of yeast- and hypha-specific culture media grown during 16 h at 37°C and 180 rpm were collected by 20 min of centrifugation at 8,000 rpm at 4°C in a Beckman Coulter J2-HS centrifuge using the JA-10 rotor. The supernatants were then filtrated using a 0.45-μm filter to ensure the elimination of all the cells and cell debris. One protease inhibitor tablet (Pierce, EDTA-free; Thermo Fisher) along with 1 mL of phenylmethanesulfonylfluoride (PMSF) was added to each of the 1-L volumes of filtrated supernatants. These supernatants were concentrated afterwards, using a Centricon plus-70 filter (100-kDa-cutoff filter; Millipore), by centrifugation at 2,500 rpm in an Eppendorf 5810R centrifuge to a final volume of 8 mL. The concentrated supernatants were subsequently ultracentrifuged at 100,000 × g (34,200 rpm) for 1 h at 4°C in a Beckman Optima XL-90 using a 90 Ti rotor. The pellets containing the isolated EVs were washed twice with PBS and solubilized in 50 μL of 0.5 M triethylammonium bicarbonate (TEAB) buffer. Protein concentration was measured using the Bradford protein assay (Bio-Rad), following the manufacturer’s instructions.

### TEM.

Transmission electron microscopy (TEM) was used to visualize EVs isolated from both yeast and hyphal-cell morphologies. Samples were fixed for 2 h at room temperature in a buffer containing 2.5% glutaraldehyde and 0.1 M cacodylate and then incubated overnight at 4°C in 4% paraformaldehyde, 1% glutaraldehyde, and 0.1% PBS. After that, the samples were treated with 2% osmium tetroxide (TAAB Laboratories, UK) for 90 min, serially dehydrated in ethanol, and embedded in EMBed-812 resin (Electron Microscopy Sciences). Thin sections (50 to 70 nm) were obtained by ultracut and observed in a JEOL JEM 1010 transmission electron microscope operating at 100 kV. Pictures were taken with a Megaview II camera. TEM images were analyzed with Soft Imaging Viewer software. TEM was carried out in the Electron Microscopy Facility (ICTS) of the Complutense University of Madrid (UCM).

### Analysis of vesicles by DLS.

EV sizes (Z average diameter) were measured by dynamic light scattering (DLS) using a Zetasizer (Nano ZS; Malvern). Three biological replicates of EVs (from both types of cells, yeast and hyphal) were transferred to a disposable cuvette, and 10 measurements for each were performed with the refractive index at 1.33 and absorption at 0.01. Data analysis was performed using Zetasizer software 7.11 (Malvern). DLS was carried out at the spectroscopy and correlation facility of the Complutense University of Madrid (UCM).

### Whole-cell lysate (WCL) protein extraction.

Three independent experiments were done with yeast and hyphal cultures. All the processes were conducted at 4°C. Amounts of 20 mL of yeast- and hypha-specific culture media grown during 16 h at 37°C and 180 rpm were collected in a 50-mL Falcon tube by 10 min of centrifugation at 2,500 rpm at 4°C in an Eppendorf 5810R centrifuge. The cell pellets were subsequently washed twice with 20 mL of ice-cold PBS and transferred to a 2-mL Eppendorf tube. Equal volumes of 0.45-mm glass beads were added. The cell pellets were disrupted in 500 μL of lysis buffer (50 mM Tris-HCl, pH 7.5, 1 mM EDTA, 150 mM NaCl, 1 mM dithiothreitol [DTT]) with 1 mM PMSF and protease inhibitor cocktail tablets (Roche) by vigorous shaking in a FastPrep cell breaker (Bio 101) (level 5.5, 5 times for 30 s). Cell debris and glass beads were removed by centrifugation (13,000 rpm for 15 min), and the cell extracts were collected in a new Eppendorf tube. Protein quantification was performed using the Bradford assay (Bio-Rad, Hercules, CA, USA), and protein samples were stored at −80°C.

### SDS-PAGE and Western blotting.

The EVs’ protein extracts (30 μg of each) were denatured by heating for 5 min at 99°C in SDS-containing buffer (4% SDS, 100 mM Tris HCl, pH 6.8, 20% glycerol, 0.2% bromophenol blue, and 20% DTT). Protein samples were separated in 10% SDS–polyacrylamide gels using the Mini-Protean II electrophoresis system (Bio-Rad). The gel was stained with a fixative solution of 40% methanol (MeOH), 10% acetic acid (vol/vol), and Coomassie brilliant blue G-250 (Bio-Rad). For Western blotting, 30 μg of EV protein extracts were separated in 10% SDS-polyacrylamide gels, transferred to nitrocellulose membranes, and blocked in 5% milk–PBS. Western blots were probed with sera from patients suffering invasive candidiasis at a dilution of 1:3,000 (72). After an overnight incubation with the sera, membranes were washed five times with 0.1% Tween 20 containing PBS and then incubated with fluorescently labeled secondary antibodies at a dilution of 1/1,000 (IR dye 800-labeled goat anti-human IgG; LI-COR Biosciences). The Western blotting was performed with the Odyssey system (LI-COR Biosciences, NE, USA).

### Digestion and desalting of peptides.

In-gel protein digestion is useful to eliminate contaminants that could interfere with MS/MS analyses. For this, 25 μg of each protein extract was concentrated in a stacking gel, and protein bands were cut from the acrylamide gel for in-gel trypsin digestion. Briefly, cut protein bands were first reduced with DTT (Sigma-Aldrich, St. Louis, MO, USA), then treated with iodoacetamide for protein alkylation (Sigma-Aldrich, St. Louis, MO, USA), and ultimately digested with 1.25 μg of recombinant trypsin (sequencing grade; Roche, Mannheim, Germany) overnight at 37°C (73). C18 reverse-phase chromatography was used for desalting and concentration of the peptides from the digested proteins (Poros R2; Applied Biosystems), which were then eluted with 80% acetonitrile/0.1% trifluoroacetic acid (Thermo Fisher Scientific). The elution buffer was then evaporated in a SpeedVac vacuum concentrator (Thermo Fisher Scientific, Rockford, IL, USA), and the freeze-dried samples resuspended in 2% acetonitrile, 0.1% formic acid (Thermo Fisher Scientific, Rockford, IL, USA) before performing nanoscale liquid chromatography coupled with mass spectrometry in tandem (LC-MS/MS).

### LC-MS/MS.

The desalted peptides were analyzed by reversed-phase liquid chromatography-electrospray ionization-tandem mass spectrometry (RP-LC-ESI-MS/MS) in an Ultimate 3000 nLC (Thermo Fisher Scientific) coupled to an Orbitrap Fusion Lumos Tribrid mass spectrometer (Thermo Fisher) through an EasySpray nano emitter (all from Thermo Scientific, Bremen, Germany). Peptides were loaded first onto an Acclaim PepMap 100 trap column (20 mm, 75-μm inner diameter [ID], 3 μm of C_18_ resin with 100-Å pore size; Thermo Scientific, Germering, Germany) using buffer A (mobile phase A: 2% acetonitrile, 0.1% formic acid) and then separated and eluted on a C_18_ resin analytical column NTCC (50 cm, 75-μm ID, 3-μm C_18_ resin with 100-Å pore size; Nikkyo Technos Co., Ltd., Tokyo, Japan) with an integrated spray tip. The analysis was performed with a 95-min gradient of 5% to 27% buffer B (100% acetonitrile, 0.1% formic acid), a 5-min gradient of 27% to 44% buffer B, and finally, 10 min more to 95% buffer A at a constant flow rate of 0.3 μL/min.

All data were acquired using data-dependent acquisition (DDA) in positive mode with Xcalibur 4.0 software (Thermo Fisher Scientific, Inc., USA). For the MS^2^ scan, the top 15 most abundant precursors with charges of 2 to 7^+^ selected in MS^1^ scans were selected for higher-energy collisional dissociation (HCD) fragmentation with a dynamic exclusion of 60 s. The MS^1^ scans were acquired at an *m*/*z* range of 375 to 1,500 Da with a Orbitrap mass resolution of 120,000 and an automatic gain control (AGC) target of 4E5 at a maximum ion time (IT_max_) of 50 ms. The threshold to trigger MS^2^ scans was 5E3, the normalized collision energy (NCE) was 30%, and the resolved fragments were scanned at a mass resolution of 30,000 and an AGC target value of 1E4 in an IT_max_ of 60 ms.

### Protein identification.

Peptide identifications from raw data were carried out using the Mascot version 2.6.1 (MatrixScience, London, UK) search engine through the Protein Discoverer 2.4 software (Thermo Fisher Scientific, Waltham, MA, USA). A database search was performed against Candida albicans CGD21 (6,209 sequences) from http://www.candidagenome.org. The following parameters were used for the searches. For tryptic cleavage, up to two missed cleavage sites were allowed, with tolerances of 10 ppm for precursor ions and 0.02 Da for MS/MS fragment ions, and the searches were performed allowing optional methionine oxidation and acetyl protein N-terminal and fixed carbamidomethylation of cysteine. A search against the decoy database (integrated decoy approach) was used to calculate the false discovery rate (FDR). The Mascot scores were adjusted by a percolator algorithm. The acceptance criterion for protein identification was an FDR of <0.01. The mass spectrometry proteomics data have been deposited to the ProteomeXchange Consortium via the PRIDE partner repository with the data set identifiers PXD021488 and PXD021504. As an estimation of the relative protein abundances, the normalized spectral abundance factor (NSAF) was used, and the average of the normalized values was calculated (74).

### Bioinformatic analysis.

We used the Candida Genome Database (CGD; www.candidagenome.org) for the analyses. Proteins that were identified in at least two replicates with at least two peptides in one of them were used for the analysis. Venn diagrams were prepared using the Venn tool available in the program FunRich 3.1.3 (75). The GO enrichment analysis of the set of proteins identified in yeast or hyphal EVs was done using the Gene Ontology (GO) annotation application (http://www.candidagenome.org/cgi-bin/GO/goTermFinder) from CGD and the GO enrichment analysis from the FunRich 3.1.3 program, which is based on a UniProt database and uses protein homologues from all fungi (75).

Metabolic pathways were retrieved from the KEGG database (https://www.genome.jp/kegg/) (76).

### THP-1 cell culture and macrophage differentiation.

THP-1 cells (human acute monocytic leukemia cell line) were grown and maintained in Dulbecco’s modified Eagle’s medium (DMEM) supplemented with antibiotics (10,000 U/mL each of penicillin and streptomycin), 2 mM l-glutamine, and 10% heat-inactivated fetal bovine serum (FBS). THP-1 cultures were incubated in a humidified atmosphere containing 5% CO_2_ at 37°C. Twenty-four-well plastic plates were seeded with THP-1 cells at a density of 3 × 10^5^ cells per well in complete medium after being treated with 30 ng/mL phorbol 12-myristate 13-acetate (PMA; Sigma-Aldrich). These 24-well plastic plates were then incubated for 48 h to induce maturation mediated by PMA toward adherent macrophage-like cells. After this 48-h period, the medium containing PMA was replaced with fresh medium without PMA to remove unattached cells.

### Determination of cytokine production.

For cytokine measurements, differentiated macrophages from the THP-1 cell line were incubated for 8 and 24 h with or without 5 μg of YEVs or HEVs. As a positive control, macrophages were treated with lipopolysaccharide (LPS) (100 ng/mL). After the corresponding incubation period, supernatants from THP-1 macrophages (untreated, LPS treated [1,000 ng/mL], and YEVS or HEVS treated) were collected. They were tested for cytokine production by enzyme-linked immunosorbent assay (ELISA) using matched paired antibodies specific for IL-12p40, TNF-α, and IL-10 (ImmunoTools) according to the manufacturer’s instructions. Cytokine concentrations were measured spectrophotometrically at 450 nm in a total of 3 biological replicates.

### Macrophage damage assay.

A colorimetric assay based on the measurement of LDH activity released by damaged cells was used (Roche). Experiments were performed in 96-well plates, and the manufacturer’s instructions were followed. Briefly, differentiated macrophages from the THP-1 cell line were incubated for 8 h with or without 5 μg of YEVs or HEVs. Lysis buffer was added to the positive-control cells 15 min before the end of the incubation time. To determine LDH activity, 100 μL of reaction mixture (catalyst and dye solution) was added to each well on the 96-well plate and incubated (protected from light) for up to 30 min at room temperature. After this, 50 μL of stop solution (2 M H_2_SO_4_) was added to each well and the absorbance at 490 nm was measured for each one. Cytotoxicity was calculated as follows: % cytotoxicity = (experimental value − low control)/(high control − low control) × 100.

Three biological replicates were performed.

### Proteasome activity assay.

A fluorometric assay based on the chymotrypsin-like protease activity associated with the proteasome complex was used (Sigma-Aldrich). Experiments were performed in triplicates in black 96-well plates with clear flat bottoms following the manufacturer’s instructions. Briefly, different tested amounts of YEVs and HEVs were added to PBS to a final volume of 100 μL. Negative and positive controls were also included by adding PBS or PBS with 100 μg of cytoplasmic C. albicans extract, respectively. This cytoplasmic C. albicans extract used as the positive control must be prepared without adding protease inhibitors. Then, 100 μL of proteasome assay loading solution was added to each well. Plates were incubated at 37°C overnight protected from light. The fluorescence intensity (directly related to proteasome activity) was monitored at an excitation wavelength (λ_ex_) of 490 nm and emission wavelength (λ_em_) of 525 nm.

Three biological replicates were performed.

### Blue native PAGE.

Separation of complexes from protein samples can be carried out by means of electrophoresis in a native PAGE bis-Tris gel system under native (nondenaturing) conditions. The NativePAGE Novex gel system is based on the blue native polyacrylamide gel electrophoresis (BN-PAGE) technique developed by Schagger et al. (77) that uses Coomassie G-250 as a charge shift molecule. The NativePAGE system from Life Technologies was used as reported before (78). HEV and YEV samples, each containing 25 μg of protein, were mixed with 4× NativePAGE sample buffer (Life Technologies) and the nonionic detergent Nonidet to a final concentration of 0.1%, and 1% NativePAGE G-250 sample additive (Life Technologies) was added to a final concentration of 0.01%. The electrophoresis was performed using NativePAGE 3 to 12% bis-Tris gels (Life Technologies). NativeMark unstained protein standard (20 kDa to 1.2 MDa; Life Technologies) was used as a molecular weight marker. Electrophoretic buffers were prepared and used according to the manufacturer’s protocol (Life Technologies). Gels were fixed in 40% methanol and 2% acetic acid for 30 min and then left in water until further processing.

### Statistical analysis.

Bar graphs were plotted and statistical analyses (unpaired *t* test) were performed using GraphPad Prism 8. Results represent the average values from at least three biological replicates.

### Data availability.

The data set from this paper have been deposited in the ProteomeXchange Consortium 554 via the PRIDE partner repository with the data set identifiers PXD021488 and PXD021504.
