# Supplementary material for: Candida albicans Hyphal Extracellular Vesicles Are Different from Yeast Ones, Carrying an Active Proteasome Complex and Showing a Different Role in Host Immune Response
Source: Microbiol Spectr. 2022 May 23;10(3):e00698-22. doi: 10.1128/spectrum.00698-22 (PMC9241596; doi:10.1128/spectrum.00698-22)
Supplement: SUPPLEMENTAL FILE 1 — Supplemental material. Download spectrum.00698-22-s001.pdf, PDF file, 2.9 MB [file spectrum.00698-22-s001.pdf]

Supplementary materials

***Candida albicans* hyphal extracellular vesicles are different to yeast ones, carrying an active proteasome complex and showing a different role in host immune response**

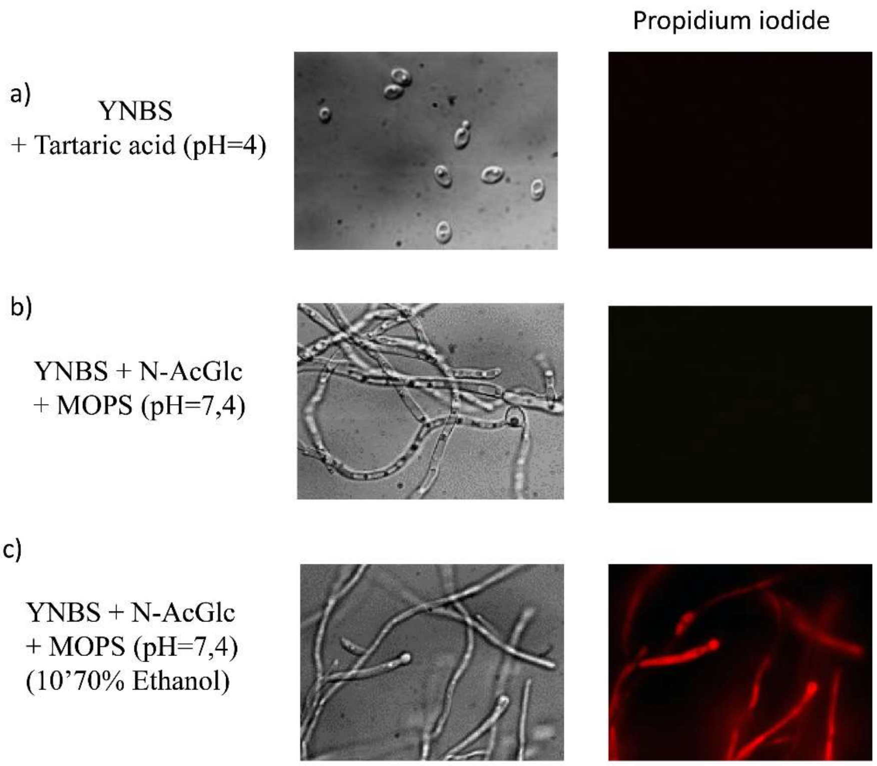

Figure S1. Phase-contrast micrographs and PI stained micrographs of *C. albicans* yeast and hyphal cells.

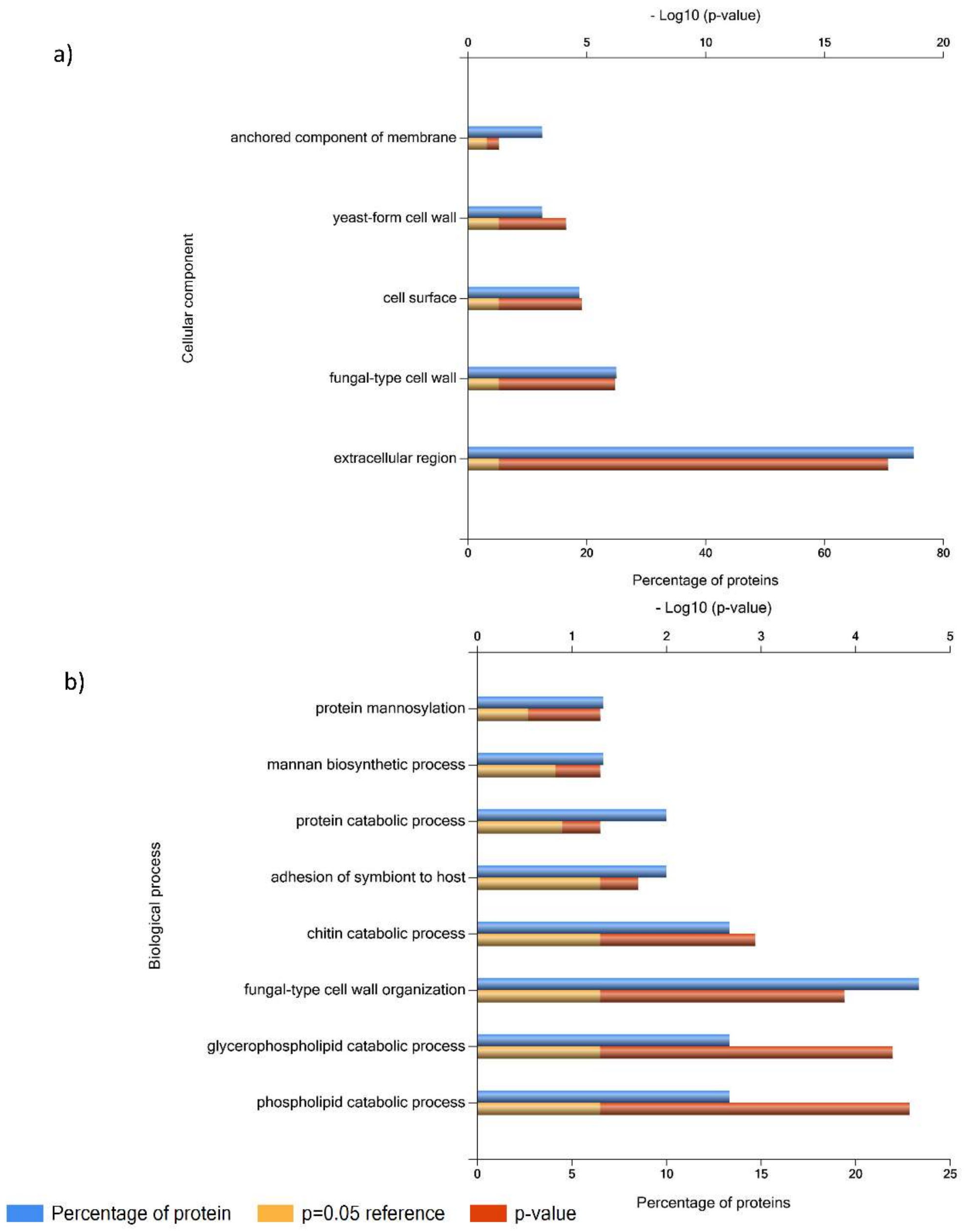

Figure S2. The FunRich categorization of component and biological process enrichment of proteins exclusively identified in (a) YEVs and (b) HEVs

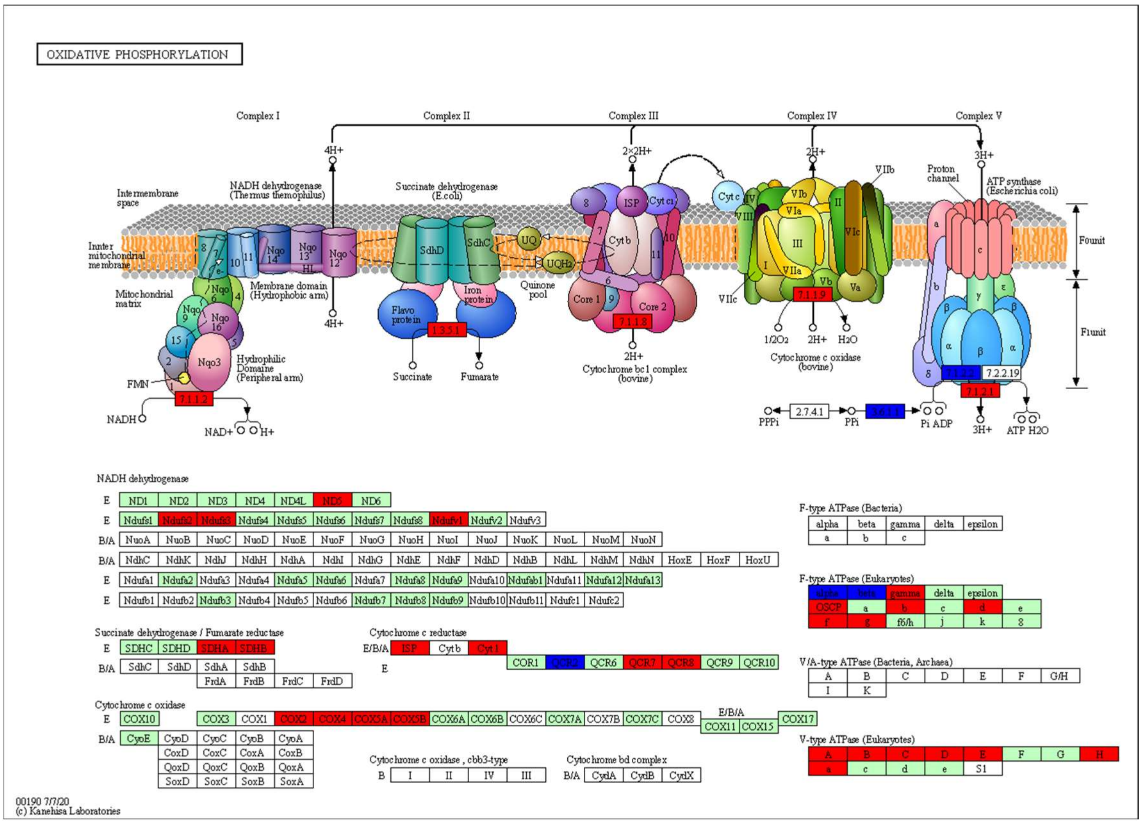

Figure S3 The KEEG pathways related to oxidative phosphorylation. The proteins exclusively identified in HEVs are filled in red while the proteins identified in EVs from both types of morphologies are filled in Blue. The proteins described in *C. albicans* but not identified in this study are filled in green while the proteins with no *C. albicans* orthologues described so far are in a white box.

Table S1a. Proteins identified in at least 2 replicates of YEVs with at least 2 peptides in a replicate (listed in decreasing order of abundance (NSAF).

	Description	Coverage [%]	PSMs	MW [kDa]	NSAF
SIM1	Adhesin-like protein	54	833	39,4	0,058991
TOS1	Protein similar to alpha agglutinin anchor subunit	40	995	49,4	0,056199
MP65	Cell surface mannoprotein	35	759	39,2	0,054024
SUN41	Cell wall glycosidase	37	792	43,7	0,050568
SCW11	Cell wall protein	46	620	54,3	0,031859
PGA4	GPI-anchored cell surface protein	42	393	49	0,022378
RBE1	Pry family cell wall protein	28	225	29,1	0,021574
XOG1	Exo-1,3-beta-glucanase	52	369	50	0,020592
TDH3	NAD-linked glyceraldehyde-3-phosphate dehydrogenase	77	226	35,8	0,017614
PLB4.5	Phospholipase B	32	480	77	0,017393
RHD3	GPI-anchored yeast-associated cell wall protein	47	131	21,5	0,017001
TAL1	Transaldolase	65	210	35,7	0,016413
BGL2	Cell wall 1,3-beta-glucosyltransferase	17	188	33,6	0,015612
ENO1	Enolase	49	264	47,2	0,015606
KAR2	Similar to Hsp70 family chaperones	46	387	74,5	0,014494
ECM33	GPI-anchored cell wall protein	32	220	43,5	0,014111
DAG7	Secretory protein	20	113	26,3	0,011988
PGK1	Phosphoglycerate kinase	65	168	45,2	0,010371
CYP5	Putative peptidyl-prolyl cis-trans isomerase	65	82	22,4	0,010214
ADH1	Alcohol dehydrogenase	49	127	36,8	0,009629
HSP70	Putative hsp70 chaperone	43	225	70,3	0,00893
SSA2	HSP70 family chaperone	49	222	70	0,008849
KRE9	Protein of beta-1,6-glucan biosynthesis	19	90	29,1	0,008629
CRH11	GPI-anchored cell wall transglycosylase, putative ortholog of S. cerevisiae Crh1p	27	144	46,7	0,008604
MET6	Essential 5-methyltetrahydropteroyltriglutamate-homocysteine methyltransferase (cobalamin-independent methionine synthase)	44	249	85,6	0,008116
COI1	Secreted protein	18	60	21,3	0,00786
CYP1	Peptidyl-prolyl cis-trans isomerase	49	49	17,6	0,007768
orf19.4150	Putative glutaredoxin	45	70	25,5	0,007659
SAP3	Secreted aspartyl proteinase, acts in utilization of protein as nitrogen source	45	117	42,8	0,007627
TRX1	Thioredoxin	41	31	11,5	0,007521
orf19.3226	Protein of unknown function	33	54	21,2	0,007107
RBT4	Pry family protein	20	95	37,4	0,007087
UTR2	Putative GPI anchored cell wall glycosidase	23	130	51,7	0,007016
TSA1	TSA/alkyl hydroperoxide peroxidase C (AhPC) family protein	60	53	21,8	0,006783
ENG1	Endo-1,3-beta-glucanase	26	293	124	0,006593
PDI1	Putative protein disulfide-isomerase	25	146	63	0,006466
orf19.5322	Protein of unknown function	29	62	28,3	0,006113
PDC11	Pyruvate decarboxylase	42	133	62,4	0,005947
orf19.4952.1	Protein of unknown function	22	34	16,5	0,005749
orf19.86	Putative glutathione peroxidase	43	37	18,1	0,005704
MDH1-1	Predicted malate dehydrogenase precursor	63	70	34,7	0,005629
orf19.3053	Protein of unknown function	38	40	20,3	0,005498

HSP90	Essential chaperone, regulates several signal transduction pathways and temperature-induced morphogenesis	34	146	80,8	0,005042
PHR2	Glycosidase	22	104	58,7	0,004943
GDA1	Golgi membrane GDPase, required for wild-type O-mannosylation, not N-glycosylation	30	116	65,9	0,004911
IDP2	Isocitrate dehydrogenase	36	79	46,3	0,004761
GLX3	Glutathione-independent glyoxalase	41	43	25,8	0,00465
ARF2	Putative ADP-ribosylation factor	48	34	20,6	0,004605
GND1	6-phosphogluconate dehydrogenase	29	89	54,3	0,004573
orf19.6484	Protein of unknown function	26	27	16,5	0,004566
GDH3	NADP-glutamate dehydrogenase	35	80	49,5	0,004509
orf19.7578	Protein of unknown function	26	47	29,4	0,004461
orf19.2870	Protein of unknown function	18	26	16,6	0,00437
CHT3	Major chitinase	17	93	60	0,004325
FBA1	Fructose-bisphosphate aldolase	39	59	39,2	0,0042
TEF2	Translation elongation factor 1-alpha	22	73	50	0,004074
HET1	Putative sphingolipid transfer protein	47	31	21,8	0,003968
ASC1	40S ribosomal subunit similar to G-beta subunits	31	49	34,5	0,003963
TPI1	Triose-phosphate isomerase	34	37	26,6	0,003881
PLB1	Phospholipase B	27	91	66,4	0,003824
orf19.1766	Secreted protein	25	60	45	0,00372
LDG3	Putative LDG family protein	26	21	16,4	0,003573
PGI1	Glucose-6-phosphate isomerase	23	78	61,1	0,003562
AHP1	Alkyl hydroperoxide reductase	45	24	19,2	0,003488
CDC19	Pyruvate kinase at yeast cell surface	35	69	55,4	0,003475
YNK1	Nucleoside diphosphate kinase (NDP kinase)	41	21	16,9	0,003467
CHT1	Chitinase	14	60	48,9	0,003424
SAP10	Secreted aspartyl protease	13	60	49,3	0,003396
MET15	O-acetylhomoserine O-acetylserine sulfhydrylase	36	57	48	0,003313
BAT22	Putative branched chain amino acid aminotransferase	23	47	40,8	0,003214
orf19.6119	Protein of unknown function	12	86	75,4	0,003182
orf19.1946	Similar to an aldose 1-epimerase-related protein	18	38	33,8	0,003137
RPL12	Ribosomal protein L12, 60S ribosomal subunit	33	20	17,8	0,003135
GPM1	Phosphoglycerate mutase	34	30	27,4	0,003055
orf19.6487	Protein of unknown function	20	18	16,7	0,003007
orf19.4898	Putative protein of unknown function	21	26	24,3	0,002985
RDI1	Putative rho GDP dissociation inhibitor	38	24	22,9	0,002924
GRP2	Methylglyoxal reductase	28	39	37,6	0,002894
HHF1	Putative histone H4	21	12	11,6	0,002886
HOM6	Putative homoserine dehydrogenase	41	40	38,8	0,002876
MNT1	Alpha-1,2-mannosyl transferase	29	49	50	0,002734
SUR7	Protein required for normal cell wall, plasma membrane, cytoskeletal organization, endocytosis	36	28	29,9	0,002613
UBI3	Fusion of ubiquitin with the S34 protein of the small ribosomal subunit	13	21	22,6	0,002593
SAP7	Pepstatin A-insensitive secreted aspartyl protease	20	58	62,5	0,002589
orf19.2757	Protein of unknown function	19	36	39,2	0,002562
PIR1	1,3-beta-glucan-linked cell wall protein	13	33	36,5	0,002523
RBT7	Protein with similarity to RNase T2 enzymes	17	33	36,8	0,002502
GLK1	Putative glucokinase	26	46	52,1	0,002464
ERO1	Ortholog of S. cerevisiae Ero1	11	59	66,9	0,002461
APE3	Putative vacuolar aminopeptidase Y,	17	53	60,8	0,002432

orf19.6741	Putative plasma membrane protein	17	39	44,9	0,002424
orf19.5925	Protein of unknown function	16	37	43,5	0,002373
GRE3	Putative D-xylose reductase	18	36	42,4	0,002369
BMH1	Sole 14-3-3 protein in C. albicans	20	25	29,5	0,002365
YPS7	Putative aspartic-type endopeptidase with limited ability to degrade alpha pheromone	12	62	75,8	0,002282
PEP1	Type I transmembrane sorting receptor for multiple vacuolar hydrolases	19	123	150,4	0,002282
SAH1	S-adenosyl-L-homocysteine hydrolase	24	40	49	0,002278
RPL14	Ribosomal protein L14	23	12	14,7	0,002278
FDH1	Formate dehydrogenase	22	34	41,8	0,00227
PRB1	Endoprotease B	13	40	50,2	0,002223
LHS1	Protein similar to S. cerevisiae Hsp70p	19	83	104,8	0,00221
orf19.2460	Protein of unknown function	18	29	37	0,002187
ERV25	Component of COPII-coated vesicles	20	19	24,5	0,002164
SAP9	Secreted aspartyl protease	17	45	58,5	0,002146
HXK2	Hexokinase II	26	41	53,4	0,002142
PRX1	Thioredoxin peroxidase	23	21	27,4	0,002138
YWP1	Secreted yeast wall protein	5	41	54,2	0,002111
ADH2	Alcohol dehydrogenase	22	27	36,8	0,002047
MCR1	NADH-cytochrome-b5 reductase	24	24	33,4	0,002005
PBR1	Protein of unknown function	17	14	19,6	0,001993
ACT1	Actin	19	29	41,7	0,00194
IPP1	Putative inorganic pyrophosphatase	26	22	32,1	0,001912
PRC2	Putative carboxypeptidase	20	38	56,3	0,001883
APR1	Vacuolar aspartic proteinase	23	30	45,4	0,001844
INO1	Inositol-1-phosphate synthase	23	38	57,7	0,001838
KEX2	Subtilisin-like protease (proprotein convertase)	14	69	104,8	0,001837
TKL1	Putative transketolase	30	48	73,7	0,001817
TMA19	Cell wall protein, ortholog of S. cerevisiae Tma19p (Ykl065cp)	17	12	18,5	0,00181
OSM1	Putative flavoprotein subunit of fumarate reductase	18	35	54,3	0,001798
PET9	Mitochondrial ADP/ATP carrier protein involved in ATP biosynthesis	22	21	32,7	0,001792
FMP45	Predicted membrane protein induced during mating	20	20	31,2	0,001789
RHO1	Small GTPase of Rho family	20	14	22	0,001776
SBA1	Similar to co-chaperones	16	15	23,9	0,001751
orf19.1054	Protein of unknown function	11	19	30,5	0,001738
FET99	Multicopper oxidase family protein	8	44	71,3	0,001722
RPL10A	Predicted ribosomal protein	14	15	24,4	0,001715
PGA45	Putative GPI-anchored cell wall protein	13	30	48,9	0,001712
SEC14	Essential protein	25	21	34,7	0,001689
PFY1	Profilin	45	8	13,8	0,001618
COF1	Putative cofilin	44	9	15,8	0,001589
SAM2	S-adenosylmethionine synthetase	21	24	42,2	0,001587
NTF2	Putative nuclear envelope protein	19	8	14,2	0,001572
orf19.1765	Secreted protein	14	25	47,3	0,001475
LEU2	Isopropyl malate dehydrogenase	18	21	40,1	0,001461
PGM2	Ortholog of S. cerevisiae Pgm2	18	32	61,8	0,001445
ERG10	Acetyl-CoA acetyltransferase	22	21	41,9	0,001398
HOM2	Aspartate-semialdehyde dehydrogenase	18	19	39,2	0,001352
PMM1	Phosphomannomutase	20	14	29	0,001347
YPT31	Protein required for resistance to toxic ergosterol analog	14	12	25,1	0,001334
MNN2	Alpha-1,2-mannosyltransferase	15	33	69,1	0,001333

DCW1	Protein with predicted GPI modification	10	24	50,3	0,001331
ANB1	Translation initiation factor eIF-5A	11	8	17,1	0,001305
orf19.3910	Protein of unknown function	14	21	45,7	0,001282
PGA52	GPI-anchored cell surface protein of unknown function	12	19	41,4	0,001281
ATP1	ATP synthase alpha subunit	15	27	58,9	0,001279
PHO8	Putative repressible vacuolar alkaline phosphatase	12	25	55	0,001268
TIF	Translation initiation factor	15	20	44,6	0,001251
FBP1	Fructose-1,6-bisphosphatase	16	16	35,9	0,001244
MNN23	Alpha-1,2-mannosyltransferase	12	31	69,6	0,001243
TFS1	Putative carboxypeptidase y inhibitor	20	13	29,3	0,001238
FDH3	Glutathione-dependent formaldehyde dehydrogenase	17	18	40,6	0,001237
orf19.540	Protein of unknown function	22	16	36,4	0,001226
orf19.7306	Aldo-keto reductase	28	17	39	0,001216
HIS1	ATP phosphoribosyl transferase	16	14	32,6	0,001198
SRB1	Essential GDP-mannose pyrophosphorylase	20	17	40	0,001186
PST3	Putative flavodoxin	19	9	21,2	0,001185
CIP1	Possible oxidoreductase	16	14	33	0,001184
EXG2	GPI-anchored cell wall protein, similar to S. cerevisiae exo-1,3-beta-glucosidase Exg2p	12	23	54,5	0,001178
SER33	Predicted enzyme of amino acid biosynthesis	13	21	50,3	0,001165
MDH1	Mitochondrial malate dehydrogenase	22	15	36	0,001163
IFR2	Zinc-binding dehydrogenase	16	16	38,5	0,00116
ALD5	NAD-aldehyde dehydrogenase	25	22	53,9	0,001139
MNT2	Alpha-1,2-mannosyl transferase	10	22	54,5	0,001126
FMA1	Putative oxidoreductase	11	11	27,4	0,00112
ZWF1	Glucose-6-phosphate dehydrogenase	13	23	58,3	0,001101
GCY1	Aldo/keto reductase	16	13	33	0,001099
SSB1	HSP70 family heat shock protein	13	26	66,4	0,001093
ATP2	F1 beta subunit of F1F0 ATPase complex	18	21	53,9	0,001087
CHT2	GPI-linked chitinase	8	23	60,8	0,001056
CDC42	Rho-type GTPase	10	8	21,2	0,001053
orf19.4609	Putative dienelactone hydrolase	19	10	26,7	0,001045
GSP1	Small RAN G-protein	14	9	24,3	0,001033
ARG1	Argininosuccinate synthase	13	17	46,1	0,001029
orf19.3558	Protein of unknown function	8	9	24,6	0,001021
CAT1	Catalase	14	20	54,8	0,001018
orf19.1785	Protein with a PI31 proteasome regulator domain	7	13	35,9	0,00101
HAL22	Putative phosphoadenosine-5'-phosphate or 3'-phosphoadenosine 5'-phosphosulfate phosphatase	13	14	38,7	0,001009
HAL21	Putative phosphoadenosine-5'-phosphate or 3'-phosphoadenosine 5'-phosphosulfate phosphatase	14	13	36,8	0,000986
orf19.6507	Putative curved DNA-binding protein orthologous to S. pombe Cdb4	12	15	43,1	0,000971
YPT1	Functional homolog of S. cerevisiae Ypt1p, which is an essential small Ras-type GTPase involved in protein secretion at ER-to-Golgi	7	8	23	0,000971
SSU81	Predicted adaptor protein involved in activation of MAP kinase-dependent signaling pathways	12	14	41,1	0,00095
orf19.2484	Protein of unknown function	9	16	47,4	0,000942
orf19.2516	Protein of unknown function	10	9	27,4	0,000916
PMI1	Phosphomannose isomerase	10	16	48,8	0,000915
PHR1	Cell surface glycosidase	12	19	59,5	0,000891
HAM1	Putative deoxyribonucleoside triphosphate pyrophosphohydrolase	10	7	22	0,000888
orf19.4211	Multicopper oxidase	5	22	70,6	0,000869

CIT1	Citrate synthase	8	16	52	0,000859
APE2	Neutral arginine, alanine, leucine specific metallo-aminopeptidase	16	32	104,3	0,000856
orf19.2047	Putative protein of unknown function	13	7	24	0,000814
SHM2	Cytoplasmic serine hydroxymethyltransferase	15	15	52	0,000805
ADO1	Adenosine kinase	16	11	38,2	0,000803
SOL3	Putative 6-phosphogluconolactonase	14	8	28,5	0,000783
GLO1	Putative monomeric glyoxalase I	8	11	39,5	0,000777
orf19.7502	Protein of unknown function	11	8	29,5	0,000757
IDP1	Putative isocitrate dehydrogenase	10	13	48,5	0,000748
PLB2	Putative phospholipase B	7	18	67,3	0,000746
AGM1	Phosphoacetylglucosamine mutase (N-acetylglucosamine-phosphate mutase)	10	16	60,4	0,000739
MSB2	Mucin family adhesin-like protein	5	37	141	0,000732
RAX2	Plasma membrane protein involved in establishment of bud sites and linear direction of hyphal growth	4	40	154,3	0,000723
MRF1	Putative mitochondrial respiratory protein	8	10	38,6	0,000723
ADE13	Adenylosuccinate lyase	7	14	54,4	0,000718
CYS3	Cystathionine gamma-lyase	19	11	42,9	0,000715
CAR2	Ornithine aminotransferase	14	12	47,3	0,000708
ASN1	Putative asparagine synthetase	9	16	64,6	0,000691
orf19.6809	Putative phosphomutase-like protein	11	9	37,3	0,000673
ARO8	Aromatic transaminase of the Ehrlich fusel oil pathway of aromatic alcohol biosynthesis	9	13	54,7	0,000663
HEX1	Beta-N-acetylhexosaminidase/chitobiase, highly glycosylated enzyme that is secreted to the periplasm and culture medium	8	15	63,4	0,00066
XYL2	D-xylulose reductase	7	9	38,7	0,000649
orf19.3915	Putative metallodipeptidase	15	11	47,9	0,000641
QCR2	Ubiquinol-cytochrome-c reductase	17	9	39,5	0,000636
LPD1	Putative dihydrolipoamide dehydrogenase	9	12	52,8	0,000634
ACH1	Acetyl-coA hydrolase	8	13	57,9	0,000626
LYS9	Saccharopine dehydrogenase	12	11	49,2	0,000624
SAP5	Secreted aspartyl proteinase	5	10	45,6	0,000612
HBR2	Putative alanine glyoxylate aminotransferase	8	10	46,4	0,000601
PLB3	GPI-anchored cell surface phospholipase B	6	15	69,7	0,0006
PHO113	Putative constitutive acid phosphatase	10	11	51,3	0,000598
orf19.7297	Putative cystathionine gamma-synthase	9	9	42,4	0,000592
orf19.6883	Putative oxysterol binding protein family	4	10	48,2	0,000579
orf19.6701	Protein with similarity to amino acid-tRNA ligase	8	16	77,3	0,000578
MNN22	Alpha-1,2-mannosyltransferase	8	14	68,2	0,000573
TRR1	Thioredoxin reductase	19	7	34,7	0,000563
ERG13	3-hydroxy-3-methylglutaryl coenzyme A synthase	12	10	49,7	0,000561
SSC1	Heat shock protein	7	14	69,7	0,00056
ADE17	5-Aminoimidazole-4-carboxamide ribotide transformylase, enzyme of adenine biosynthesis	11	13	64,9	0,000559
GCV3	Glycine decarboxylase, subunit H	10	4	20	0,000558
ARO4	3-deoxy-D-arabinoheptulosonate-7-phosphate synthase	8	8	40,3	0,000554
FET34	Multicopper ferroxidase	3	14	70,7	0,000553
PRE5	Alpha6 subunit of the 20S proteasome	10	6	31,4	0,000533
HSP60	Heat shock protein	8	11	60,1	0,000511
NIT3	Putative nitrilase	8	6	32,9	0,000509
PCK1	Phosphoenolpyruvate carboxykinase	6	11	60,9	0,000504
ILV5	Ketol-acid reductoisomerase	11	8	44,9	0,000497

RBT1	Cell wall protein with similarity to Hwp1	6	13	73,6	0,000493
orf19.6596	Putative esterase	14	6	34,2	0,00049
POL30	Similar to proliferating cell nuclear antigen (PCNA)	6	5	29	0,000481
ERG20	Putative farnesyl pyrophosphate synthetase involved in isoprenoid and sterol biosynthesis, based on similarity to S. cerevisiae Erg20p	11	7	40,7	0,00048
PRD1	Putative proteinase	12	14	81,7	0,000478
DAK2	Putative dihydroxyacetone kinase	10	10	64,9	0,00043
PHO114	Acid phosphatase	7	8	52,1	0,000428
AAT1	Aspartate aminotransferase	11	7	48,9	0,000399
PST1	Putative 1,4-benzoquinone reductase	18	3	21,1	0,000397
DFG5	N-linked mannoprotein of cell wall and membrane	6	7	50	0,000391
orf19.7196	Putative vacuolar protease	4	7	52,1	0,000375
orf19.7020	Protein similar to S. cerevisiae Kex1p, which is a pheromone-processing peptidase	5	10	78,7	0,000355
orf19.5917.3	RNA binding protein required for export of poly(A)+ mRNA from the nucleus	7	3	24	0,000349
UGA1	Putative GABA transaminase	9	6	52,6	0,000318
GCA1	Extracellular/plasma membrane-associated glucoamylase	8	12	105,6	0,000317
ACS2	Acetyl-CoA synthetase	5	8	74,3	0,0003
ACS1	Acetyl-CoA synthetase	6	8	75,1	0,000297
MSI3	Essential HSP70 family protein	5	8	78,5	0,000284
DED81	Putative tRNA-Asn synthetase	5	6	62,2	0,000269
ACO1	Aconitase	7	8	84,2	0,000265
AXL2	Ortholog of S. cerevisiae Axl2	3	9	101,8	0,000247
UBA1	Ubiquitin-activating enzyme	5	9	114,2	0,00022
EFT2	Elongation Factor 2 (eEF2)	7	7	93,3	0,000209
MID1	Putative stretch-activated Ca2+ channel of the high affinity calcium uptake system	6	4	62,6	0,000178
CDC60	Cytosolic leucyl tRNA synthetase	2	8	125,3	0,000178
orf19.4395	Putative actin cytoskeleton component	7	4	67,2	0,000166
ALS3	Cell wall adhesin	2	6	123,7	0,000135
ALS2	ALS family protein	1	12	251,1	0,000133
ROT2	Alpha-glucosidase II, catalytic subunit, required for N-linked protein glycosylation and normal cell wall synthesis	2	4	100	0,000112
PYC2	Putative pyruvate carboxylase	2	5	129,7	0,000108

Table S1b. Proteins identified in at least 2 replicates of HEVs with at least 2 peptides in a replicate (listed in decreasing order of abundance (NSAF).

	Description	Coverage [%]	PSMs	MW [kDa]	NSAF
orf19.2515	ZZ-type zinc finger protein	78	527	35,8	0,0084973
ZCF39	Zn(II)2Cys6 transcription factor	54	359	32,7	0,0063372
IFR2	Zinc-binding dehydrogenase	63	184	17	0,0062477
ZRT2	Zinc transporter	54	872	80,8	0,0062296
GCN20	YEF3-subfamily ABC family protein, predicted not to be a transporter	73	441	45,2	0,0056319
KRE30	YEF3-subfamily ABC family protein	66	307	35,7	0,0049639
DOA1	WD repeat protein	52	533	62,4	0,0049306
YPT72	Vacuolar Rab small monomeric GTPase involved in vacuolar biogenesis	44	418	49,4	0,0048843
MLT1	Vacuolar membrane transporter	65	174	20,6	0,0048757
ABG1	Vacuolar membrane protein	55	592	70,3	0,0048609
VMA2	Vacuolar H(+)-ATPase	46	622	74,5	0,0048193
APR1	Vacuolar aspartic proteinase	58	346	41,7	0,0047895
UGP1	UTP-glucose-1-phosphaturidyl transferase	51	396	50	0,0045717
orf19.5369	Uroporphyrinogen decarboxylase	62	550	70	0,0045354
FUR1	Uracil phosphoribosyltransferase	61	203	27,3	0,0042923
UCF1	Upregulated by cAMP in filamentous growth	35	324	43,7	0,0042797
UAP1	UDP-N-acetylglucosamine pyrophosphorylase, catalyzes biosynthesis of UDP-N-acetylglucosamine from UTP and N-acetylglucosamine 1-phosphate	59	352	50	0,0040637
KRE5	UDP-glucose:glycoprotein glucosyltransferase	50	400	59,5	0,0038806
GAL10	UDP-glucose 4-epimerase	70	239	36,8	0,0037489
GSY1	UDP glucose/starch glucosyltransferase	71	179	29,5	0,0035026
orf19.6260	Ubiquitin-specific protease	65	237	39,2	0,0034899
RUB1	Ubiquitin-related protein with similarity to mammalian NEDD8	50	86	14,5	0,0034236
UBA1	Ubiquitin-activating enzyme	65	93	15,7	0,0034193
QCR2	Ubiquinol-cytochrome-c reductase	59	170	29	0,0033838
PEP1	Type I transmembrane sorting receptor for multiple vacuolar hydrolases	71	259	44,6	0,0033521
TSA1	TSA/alkyl hydroperoxide peroxidase C (AhPC) family protein	48	258	45,4	0,0032803
LIG1	tRNA ligase	52	258	45,6	0,0032659
TPI1	Triose-phosphate isomerase	59	307	55,4	0,0031988
orf19.6306	Trimethylaminobutyraldehyde dehydrogenase, the third enzyme of the carnitine biosynthesis pathway	52	109	19,8	0,0031777
TPS1	Trehalose-6-phosphate synthase	56	96	17,6	0,0031486
TPS2	Trehalose-6-phosphate (Tre6P) phosphatase	66	161	29,7	0,0031291
ANB1	Translation initiation factor eIF-5A	50	263	49	0,0030982
EIF4E	Translation initiation factor eIF4E	56	481	93,3	0,0029759
SUI2	Translation initiation factor eIF2, alpha chain	60	100	19,6	0,0029451
TIF11	Translation initiation factor eIF1a	61	103	20,2	0,0029433
TIF	Translation initiation factor	42	71	14	0,0029274
SUP35	Translation factor eRF3	57	147	29,4	0,0028862
TUF1	Translation elongation factor TU	69	388	78,5	0,0028531
CEF3	Translation elongation factor 3	55	86	17,6	0,0028206
TEF2	Translation elongation factor 1-alpha	65	141	28,9	0,0028163
TUP1	Transcriptional corepressor	70	262	53,9	0,0028059
SNF4	Transcription factor	46	189	39,4	0,002769
TAL1	Transaldolase	56	210	43,9	0,0027613
TRR1	Thioredoxin reductase	32	116	24,4	0,0027442
PRX1	Thioredoxin peroxidase	49	258	54,3	0,0027427
TRX1	Thioredoxin	51	80	16,9	0,0027325
CCT5	T-complex protein 1, epsilon subunit	45	72	15,5	0,0026814
GPD2	Surface protein similar to glycerol 3-P dehydrogenase	71	106	22,9	0,0026719
SMT3	SUMO, small ubiquitin-like protein	64	229	49,5	0,0026704

MET10	Sulfite reductase	69	247	53,4	0,00267
SDH2	Succinate dehydrogenase, Fe-S subunit	52	93	20,3	0,0026445
SDH12	Succinate dehydrogenase	56	124	27,4	0,0026123
TFP1	Subunit of vacuolar H+-ATPase	58	124	27,4	0,0026123
RPN7	Subunit of the proteasome regulatory particle	50	65	14,7	0,0025524
ARP4	Subunit of the NuA4 histone acetyltransferase complex	44	100	22,7	0,0025429
COX2	Subunit II of cytochrome c oxidase, which is the terminal member of the mitochondrial inner membrane electron transport chain	20	58	13,6	0,0024617
NAD5	Subunit 5 of NADH:ubiquinone oxidoreductase (NADH:ubiquinone dehydrogenase), a multisubunit enzyme complex (complex I) of the mitochondrial inner membrane that catalyzes the first step in mitochondrial respiration	58	49	11,5	0,0024595
KEX2	Subtilisin-like protease (proprotein convertase)	46	38	9	0,0024372
CLA4	Ste20p family Ser/Thr kinase required for wild-type filamentous growth, organ colonization and virulence in mouse systemic infection	59	253	60,1	0,00243
SNZ1	Stationary phase protein	52	357	85,6	0,0024074
orf19.6658	Stationary phase enriched protein	54	41	9,9	0,0023906
ERG1	Squalene epoxidase, epoxidation of squalene to 2,3(S)-oxidosqualene	40	104	25,2	0,0023822
MTS1	Sphingolipid C9-methyltransferase	51	274	66,4	0,002382
orf19.7193	Specificity factor required for ubiquitination	65	92	22,4	0,0023708
BMH1	Sole 14-3-3 protein in C. albicans	43	74	18,1	0,00236
KIS1	Snf1p complex scaffold protein	39	65	15,9	0,0023598
GSP1	Small RAN G-protein	62	89	21,8	0,0023566
HSP21	Small heat shock protein	43	235	57,9	0,0023428
RHO1	Small GTPase of Rho family	77	169	42,2	0,0023117
SEC4	Small GTPase of Rab family	54	400	99,9	0,0023113
RPS8A	Small 40S ribosomal subunit protein	49	176	44,1	0,0023037
orf19.2711	Similar to S. cerevisiae Elp2, an Elongator complex subunit required for modification of wobble nucleosides in tRNA	17	134	33,6	0,0023021
SBP1	Similar to RNA binding proteins	60	99	25,3	0,0022588
POL30	Similar to proliferating cell nuclear antigen (PCNA)	56	66	16,9	0,0022543
OBPA	Similar to oxysterol binding protein	57	112	28,7	0,0022526
orf19.2244	Similar to oxidoreductases and to S. cerevisiae Yjr096wp	60	85	21,8	0,0022507
KAR2	Similar to Hsp70 family chaperones	32	43	11,1	0,0022361
GPX2	Similar to glutathione peroxidase	41	103	26,9	0,0022102
SBA1	Similar to co-chaperones	36	103	27,1	0,0021939
ARF3	Similar to but not orthologous to S. cerevisae Arf3	42	82	21,7	0,0021813
DJP1	Similar to bacterial DnaJ	40	119	31,8	0,0021601
orf19.1946	Similar to an aldose 1-epimerase-related protein	43	79	21,3	0,0021409
orf19.5517	Similar to alcohol dehydrogenases	51	55	14,9	0,0021307
SPC2	Signal peptidase complex component	50	92	25,1	0,0021158
SES1	Seryl-tRNA synthetase	41	122	33,3	0,0021148
SEP7	Septin, required for wild-type invasive growth in vitro but not required for virulence in a mouse model of systemic infection	39	230	63	0,0021074
CDC10	Septin, required for wild-type cell, hyphal, or chlamydospore morphology	47	171	47,2	0,0020913
CDC3	Septin	50	48	13,3	0,0020833
CDC12	Septin	67	76	21,5	0,0020405
CDC11	Septin	56	189	53,9	0,0020241
SEC26	Secretory vesicles coatomer complex protein	59	76	21,7	0,0020217
DAG7	Secretory protein	59	137	39,2	0,0020174
COI1	Secreted protein	54	62	17,8	0,0020106
orf19.1766	Secreted protein	58	132	38,2	0,0019946
orf19.1765	Secreted protein	52	79	23	0,0019827
orf19.1239	Secreted protein	46	83	24,3	0,0019716
SAP5	Secreted aspartyl proteinase	50	201	58,9	0,0019699
SAP4	Secreted aspartyl proteinase	50	39	11,6	0,0019407

SAP6	Secreted aspartyl protease	39	148	44,2	0,0019328
SAP8	Secreted aspartyl protease	46	173	52	0,0019204
SAP9	Secreted aspartyl protease	28	130	39,2	0,0019143
SAP10	Secreted aspartyl protease	36	150	45,3	0,0019114
SAM2	S-adenosylmethionine synthetase	47	49	14,8	0,0019111
SAH1	S-adenosyl-L-homocysteine hydrolase	39	80	24,3	0,0019004
LYS1	Saccharopine dehydrogenase (biosynthetic)	50	97	29,5	0,001898
LYS9	Saccharopine dehydrogenase	52	244	74,3	0,0018956
RAD23	S. cerevisiae Rad23 ortholog	39	65	19,8	0,001895
orf19.3559	S. cerevisiae ortholog Mrps35p is a structural constituent of ribosome and localizes to mitochondrial small ribosomal subunit	34	74	22,6	0,0018901
MCS7	S. cerevisiae ortholog Mcs7 has a role in reciprocal meiotic recombination	49	34	10,4	0,0018871
orf19.5627	S. cerevisiae ortholog Hek2/Khd1 is a putative RNA binding protein involved in the asymmetric localization of ASH1 mRNA	79	45	13,8	0,0018823
orf19.4521	S. cerevisiae ortholog Env9 has similarity to oxidoreductases and is proposed to have vacuolar functions, found in lipid particles	73	113	34,7	0,0018798
orf19.6559	RNA polymerase III transcription initiation factor complex (TFIIIC) subunit	34	71	21,9	0,0018714
MED18	RNA polymerase II mediator complex subunit	31	54	16,7	0,0018665
MED1	RNA polymerase II mediator complex subunit	52	52	16,1	0,0018644
orf19.6065	RNA polymerase II holoenzyme/mediator subunit	38	372	116,9	0,0018369
orf19.5917.3	RNA binding protein required for export of poly(A)+ mRNA from the nucleus	39	49	15,5	0,0018248
SNL1	Ribosome-associated protein predicted to function in protein synthesis	54	41	13	0,0018205
YST1	Ribosome-associated protein	65	72	22,9	0,0018149
RPL3	Ribosomal protein, large subunit	43	68	22	0,0017842
RPS7A	Ribosomal protein S7	49	100	32,5	0,0017761
RPS5	Ribosomal protein S5	38	27	8,9	0,0017512
RPS3	Ribosomal protein S3	40	43	14,2	0,001748
RPS21B	Ribosomal protein S21	53	126	41,9	0,0017358
MRPL3	Ribosomal protein of the large subunit, mitochondrial	27	112	37,4	0,0017286
RPL9B	Ribosomal protein L9	28	51	17,1	0,0017216
RPL7	Ribosomal protein L7	44	205	69,7	0,0016978
RPL39	Ribosomal protein L39	63	114	38,8	0,001696
RPL37B	Ribosomal protein L37	63	153	52,1	0,0016951
RPL27A	Ribosomal protein L27	58	129	44,3	0,0016809
RPL20B	Ribosomal protein L20	18	46	15,8	0,0016806
RPL19A	Ribosomal protein L19	50	116	40	0,001674
RPL17B	Ribosomal protein L17	32	50	17,4	0,0016587
RPL14	Ribosomal protein L14	50	33	11,5	0,0016564
RPL12	Ribosomal protein L12, 60S ribosomal subunit	71	74	25,8	0,0016556
RPL10	Ribosomal protein L10	31	45	15,7	0,0016545
RPS6A	Ribosomal protein 6A	69	91	32,1	0,0016364
RPL4B	Ribosomal protein 4B	52	77	27,3	0,0016281
RPS17B	Ribosomal protein 17B	53	111	39,5	0,0016221
RPL23A	Ribosomal protein	32	122	43,5	0,0016189
RPS26A	Ribosomal protein	53	122	44,4	0,0015861
RPL11	Ribosomal protein	52	152	55,5	0,0015809
RPL16A	Ribosomal protein	49	107	39,2	0,0015756
RPS25B	Ribosomal protein	61	57	20,9	0,0015743
RPL35	Ribosomal protein	55	156	57,2	0,0015743
RPL5	Ribosomal protein	47	91	33,4	0,0015727
RRP9	Ribosomal protein	47	157	57,7	0,0015706
orf19.3572.3	Ribosomal 60S subunit protein L31B	47	352	129,7	0,0015666
RPL30	Ribosomal 60S subunit protein	43	50	18,5	0,0015601
orf19.3690.2	Ribosomal 60S subunit protein	51	178	66	0,0015568
orf19.6882.1	Ribosomal 60S subunit protein	54	103	38,4	0,0015483
orf19.6220.4	Ribosomal 60S subunit protein	43	124	47	0,0015229

RNR1	Ribonucleotide reductase large subunit	53	122	46,3	0,001521
RNR21	Ribonucleoside-diphosphate reductase	42	47	18	0,0015072
CDC42	Rho-type GTPase	52	103	39,7	0,0014976
CKB1	Regulatory subunit of protein kinase CK2 (casein kinase II), beta subunit	43	69	26,6	0,0014973
MNN4	Regulator of mannosylphosphorylation of N-linked mannans to cell wall proteins	52	90	34,7	0,0014972
orf19.6554	Regulator of calcineurin	23	30	11,6	0,0014929
END3	Regulated by Gcn4p	38	26	10,1	0,001486
RSR1	RAS-related protein	40	189	73,7	0,0014803
RAS1	RAS signal transduction GTPase	42	109	42,7	0,0014735
YPT52	Rab-family GTPase involved in vacuolar trafficking, colocolizes with Vps1p and Ypt53p in late endosome	29	119	46,7	0,0014709
CDC19	Pyruvate kinase at yeast cell surface	47	260	102,8	0,0014599
PDX1	Pyruvate dehydrogenase complex protein X	42	47	18,6	0,0014586
PDC11	Pyruvate decarboxylase	45	156	61,8	0,0014571
XKS1	Putative xylulokinase	51	53	21,2	0,0014431
USO6	Putative vesicular transport protein	46	68	27,4	0,0014326
orf19.1970	Putative vacuole biogenesis protein	44	91	36,8	0,0014274
orf19.1536	Putative vacuolar transporter	36	59	24,3	0,0014015
orf19.7196	Putative vacuolar protease	33	34	14,1	0,0013919
VMA8	Putative vacuolar H+-ATPase subunit	39	50	20,8	0,0013876
orf19.364	Putative vacuolar H+-ATPase subunit	39	120	50	0,0013854
VMA5	Putative vacuolar H(+)-ATPase	38	115	48	0,001383
APE3	Putative vacuolar aminopeptidase Y,	61	155	64,9	0,0013786
UBP6	Putative ubiquitin-specific protease of the 26S proteasome	49	37	15,7	0,0013604
NPL4	Putative ubiquitin-binding protein	49	269	114,2	0,0013597
orf19.6272	Putative ubiquitin C-terminal hydrolase	46	214	90,9	0,001359
UBA4	Putative ubiquitin activating protein	25	91	38,7	0,0013573
orf19.1625	Putative ubiquinone oxidoreductase	35	53	22,6	0,0013537
QCR7	Putative ubiquinol-cytochrome-c reductase, subunit 7	32	33	14,1	0,001351
orf19.4016	Putative ubiquinol-cytochrome-c reductase	48	111	47,5	0,0013489
RIP1	Putative ubiquinol cytochrome c-reductase	53	93	39,8	0,0013488
QCR8	Putative ubiquinol cytochrome c reductase	54	83	35,6	0,0013458
RCL1	Putative U3-containing 90S preribosome processome complex subunit	32	53	22,8	0,0013418
SIK1	Putative U3 snoRNP protein	38	64	27,6	0,0013385
UTP22	Putative U3 snoRNP protein	25	28	12,1	0,0013358
NAN1	Putative U3 snoRNP protein	38	67	29	0,0013336
UTP13	Putative U3 snoRNA-associated protein	36	113	49	0,0013312
UTP4	Putative U3 snoRNA-associated protein	37	223	97,6	0,0013189
UTP5	Putative U3 snoRNA-associated protein	45	15	6,6	0,0013119
orf19.6234	Putative U2 snRNP component	45	113	50	0,0013046
SIS1	Putative Type II HSP40 co-chaperone	46	87	38,7	0,0012977
BET2	Putative Type II geranylgeranyltransferase beta subunit	51	109	48,5	0,0012973
YDJ1	Putative type I HSP40 co-chaperone	48	48	21,4	0,0012947
TPM2	Putative tropomyosin isoform 2	43	111	49,8	0,0012866
VAS1	Putative tRNA-Val synthetase	41	32	14,4	0,0012827
TYS1	Putative tRNA-Tyr synthetase	37	68	30,7	0,0012786
WRS1	Putative tRNA-Trp synthetase	43	167	75,4	0,0012785
FRS2	Putative tRNA-Phe synthetase	54	129	58,3	0,0012772
KRS1	Putative tRNA-Lys synthetase	44	99	44,9	0,0012727
HTS1	Putative tRNA-His synthetase	52	39	17,7	0,0012719
GRS1	Putative tRNA-Gly synthetase	48	21	9,6	0,0012627
GLN4	Putative tRNA-Gln synthetase	49	223	102,1	0,0012608
orf19.4931	Putative tRNA-Cys synthetase	55	113	52,6	0,0012401
DPS1-1	Putative tRNA-Asp synthetase	43	45	21	0,0012369
DED81	Putative tRNA-Asn synthetase	47	37	17,3	0,0012346
orf19.3341	Putative tRNA-Arg synthetase	15	157	73,6	0,0012313
orf19.4811	Putative tricarboxylate carrier family protein	45	55	25,8	0,0012305

SUB2	Putative TREX complex component with a predicted role in nuclear mRNA export	46	105	49,4	0,0012269
PHM7	Putative transporter	35	73	34,5	0,0012214
ERF1	Putative translation release factor 1, which interacts with stop codons and promotes release of nascent peptides from ribosomes	48	97	46,1	0,0012146
GCN3	Putative translation initiator	45	479	227,7	0,0012143
TIF4631	Putative translation initiation factor eIF4G	58	41	19,5	0,0012137
TIF34	Putative translation initiation factor eIF3, p39 subunit	39	141	67,6	0,001204
PRT1	Putative translation initiation factor eIF3	48	102	49	0,0012016
NIP1	Putative translation initiation factor	44	81	39	0,0011989
TIF5	Putative translation initiation factor	49	27	13	0,0011989
SUI1	Putative translation initiation factor	41	153	73,7	0,0011983
SUI3	Putative translation initiation factor	48	104	50,1	0,0011983
RPG1A	Putative translation initiation factor	33	44	21,2	0,001198
TIF35	Putative translation initiation factor	47	129	62,2	0,0011972
GCD2	Putative translation initiation factor	36	103	49,7	0,0011963
GCD1	Putative translation initiation factor	41	104	50,3	0,0011935
CAM1	Putative translation elongation factor eEF1 gamma	42	143	69,6	0,001186
CAM1-1	Putative translation elongation factor	49	131	63,9	0,0011834
RIA1	Putative translation elongation factor	48	100	49,1	0,0011756
TKL1	Putative transketolase	40	82	40,3	0,0011745
TCC1	Putative transcription factor/corepressor	64	43	21,2	0,0011708
BRE1	Putative transcription factor with C3HC4 zinc finger DNA-binding motif	38	101	49,9	0,0011684
SPT6	Putative transcription elongation factor	43	419	207,2	0,0011673
orf19.6539	Putative trancription modulator	49	132	65,3	0,0011668
THS1	Putative threonyl-tRNA synthetase	34	61	30,3	0,0011621
THR4	Putative threonine synthase	59	47	23,4	0,0011594
ILV1	Putative threonine dehydratase	42	46	23	0,0011545
orf19.3319	Putative thioredoxin	42	114	57,1	0,0011525
AHP2	Putative thiol-specific peroxiredoxin	38	208	104,8	0,0011457
TAF60	Putative TFIID and SAGA complex subunit	49	97	49,1	0,0011404
TOA2	Putative TFIIA small subunit	29	80	40,6	0,0011374
SVF1	Putative survival factor	42	48	24,5	0,0011309
ECM17	Putative sulfite reductase beta subunit	38	83	42,4	0,00113
SFC1	Putative succinate-fumarate transporter	52	168	86	0,0011276
LSC1	Putative succinate-CoA ligase subunit	50	77	39,5	0,0011252
LSC2	Putative succinate-CoA ligase beta subunit	26	38	19,6	0,0011191
orf19.4468	Putative succinate dehydrogenase	44	74	38,2	0,0011182
orf19.1480	Putative succinate dehydrogenase	64	36	18,6	0,0011172
ATP7	Putative subunit of the F1F0-ATPase complex	31	117	60,8	0,0011108
SKP1	Putative subunit D of kinetochore protein complex CBF3	59	109	56,8	0,0011077
MID1	Putative stretch-activated Ca2+ channel of the high affinity calcium uptake system	36	92	48	0,0011064
HET1	Putative sphingolipid transfer protein	38	86	44,9	0,0011056
SPE3	Putative spermidine synthase	40	61	31,9	0,0011038
SNX4	Putative sorting nexin	40	104	54,4	0,0011035
ENA2	Putative sodium transporter	34	139	73,1	0,0010976
SGT2	Putative small tetratricopeptide repeat (TPR)-containing protein	65	36	19,2	0,0010823
DIP2	Putative small ribonucleoprotein complex	45	79	42,4	0,0010755
GBP2	Putative single-strand telomeric DNA-binding protein	51	123	66,3	0,0010709
PIN3	Putative SH3-domain-containing protein	42	192	104,2	0,0010636
orf19.3459	Putative serine/threonine/tyrosine (dual-specificity) kinase	44	115	62,5	0,0010621
orf19.399	Putative serine/threonine protein kinase	45	85	46,2	0,001062
AKL1	Putative serine/threonine protein kinase	28	141	77	0,001057
orf19.6239	Putative serine/threonine protein kinase	48	55	30,1	0,0010548
PPT1	Putative serine/threonine phosphatase	44	228	125,2	0,0010512
LCB2	Putative serine palmitoyltransferase component	43	197	108,2	0,001051
GLG2	Putative self-glucosylating initiator of glycogen synthesis	40	78	43	0,0010471

PMR1	Putative secretory pathway P-type Ca2+/Mn2+-ATPase	27	98	54,3	0,0010418
ASM3	Putative secreted acid sphingomyelin phosphodiesterase	50	109	60,4	0,0010417
ECM29	Putative scaffold protein, assists in association of the proteasome core particle with the regulatory particle; ortholog of S. cerevisiae Ecm29;	32	22	12,2	0,0010409
SPE2	Putative S-adenosylmethionine decarboxylase	52	66	36,8	0,0010353
orf19.7234	Putative RSC chromatin remodeling complex component	30	38	21,2	0,0010347
orf19.2473	Putative RSC chromatin remodeling complex component	37	121	67,7	0,0010317
RPL25	Putative rRNA-binding ribosomal protein component of the 60S ribosomal subunit	38	77	43,1	0,0010313
PWP1	Putative rRNA processing protein	42	239	133,9	0,0010303
VPS35	Putative role in vacuolar sorting	30	57	32,2	0,0010218
NAM7	Putative role in nonsense-mediated mRNA decay	41	87	49,2	0,0010207
NPL3	Putative RNA-binding protein	41	65	36,9	0,0010168
orf19.581	Putative RNA-binding protein	47	50	28,5	0,0010127
MPT5	Putative RNA-binding protein	34	47	26,8	0,0010123
RTF1	Putative RNA polymerase II-associated Paf1 complex subunit	51	102	58,2	0,0010117
orf19.1248	Putative RNA polymerase II subunit B44	33	73	41,7	0,0010105
orf19.3349	Putative RNA polymerase II subunit B150	58	63	36	0,0010102
RPA190	Putative RNA polymerase I subunit A190	39	219	125,3	0,0010089
RPA135	Putative RNA polymerase I subunit A135	41	30	17,2	0,0010068
RPC40	Putative RNA polymerase	46	59	33,9	0,0010046
DHH1	Putative RNA helicase	47	67	38,6	0,0010019
PBP2	Putative RNA binding protein	42	90	52	0,0009991
RPL13	Putative ribosomal subunit	35	37	21,5	0,0009934
orf19.828	Putative ribosomal protein, large subunit, mitochondrial precursor	37	58	33,8	0,0009905
RPL43A	Putative ribosomal protein, large subunit	38	82	47,9	0,0009882
RPS28B	Putative ribosomal protein S28B	40	100	58,5	0,0009867
RPS19A	Putative ribosomal protein S19	36	149	87,2	0,0009863
RPS13	Putative ribosomal protein of the small subunit	48	56	32,9	0,0009825
RPS1	Putative ribosomal protein 10 of the 40S subunit	31	85	50	0,0009813
RPS14B	Putative ribosomal protein	41	70	41,3	0,0009784
RPS27	Putative ribosomal protein	36	51	30,1	0,000978
RPS15	Putative ribosomal protein	53	64	37,8	0,0009773
RPP0	Putative ribosomal protein	40	83	49,1	0,0009758
RPS20	Putative ribosomal protein	51	49	29,1	0,000972
RPL28	Putative ribosomal protein	41	126	75,1	0,0009685
RPL21A	Putative ribosomal protein	31	118	70,4	0,0009675
RPL15A	Putative ribosomal protein	50	65	38,8	0,000967
RPS23A	Putative ribosomal protein	37	68	40,6	0,0009668
ARX1	Putative ribosomal large subunit biogenesis protein	39	60	35,9	0,0009647
RNR22	Putative ribonucleoside diphosphate reductase	37	118	70,8	0,0009621
RIB5	Putative riboflavin synthase	47	65	39	0,0009621
RDI1	Putative rho GDP dissociation inhibitor	41	64	38,6	0,0009571
RHO3	Putative Rho family GTPase	31	92	55,5	0,0009569
PHO8	Putative repressible vacuolar alkaline phosphatase	41	71	42,9	0,0009553
RPN8	Putative regulatory subunit of the 26S proteasome	45	17	10,3	0,0009527
ILV6	Putative regulatory subunit of acetolacetate synthase	41	99	60,1	0,0009509
orf19.4758	Putative reductase or dehydrogenase	46	89	54,1	0,0009496
orf19.5184	Putative Ran guanyl-nucleotide exchange factor	44	48	29,2	0,0009489
orf19.5340	Putative Rab GTPase activator	38	127	77,3	0,0009484
GDI1	Putative Rab GDP-dissociation inhibitor	39	90	54,9	0,0009463
orf19.5054	Putative quinolinate phosphoribosyl transferase, involved in NAD biosynthesis	22	20	12,2	0,0009463
PDA1	Putative pyruvate dehydrogenase alpha chain	38	49	29,9	0,000946
PDB1	Putative pyruvate dehydrogenase	43	61	37,6	0,0009365
PYC2	Putative pyruvate carboxylase	39	18	11,1	0,0009361
HEM14	Putative protoporphyrinogen oxidase	49	97	60	0,0009332
PRD1	Putative proteinase	35	124	76,9	0,0009308

MET18	Putative protein with a predicted role in nucleotide excision repair (NER) and RNA polymerase II (RNAP II) transcription	38	63	39,1	0,0009301
orf19.6476	Putative protein with a predicted role in exocytic transport from the Golgi	39	67	41,8	0,0009252
SOF1	Putative protein with a predicted role in 40S ribosomal subunit biogenesis	40	65	40,7	0,0009219
PTP3	Putative protein tyrosine phosphatase	37	105	65,9	0,0009197
SEC13	Putative protein transport factor	50	73	46	0,000916
orf19.4357	Putative protein similar to S. cerevisiae Mgr3p, a subunit of the i-AAA protease supercomplex that degrades misfolded mitochondrial proteins	27	38	24	0,000914
orf19.3689	Putative protein similar to 6-phosphofructo-2-kinase/fructose-2,6-bisphosphatase	44	75	47,5	0,0009114
LTP1	Putative protein phosphatase of the PTP family (tyrosine-specific), similar to <i>S. cerevisiae</i> Ltp1p	49	57	36,1	0,0009114
orf19.4898	Putative protein of unknown function	29	26	16,5	0,0009096
orf19.1355	Putative protein of unknown function	45	95	60,4	0,0009079
orf19.1394	Putative protein of unknown function	32	83	52,8	0,0009074
orf19.3679	Putative protein of unknown function	35	143	91,3	0,0009041
orf19.2047	Putative protein of unknown function	43	124	79,2	0,0009038
orf19.3508	Putative protein of unknown function	37	66	42,3	0,0009007
orf19.5194.1	Putative protein of unknown function	33	36	23,1	0,0008996
orf19.6872	Putative protein of unknown function	42	74	47,5	0,0008993
orf19.4013	Putative protein of unknown function	48	57	36,6	0,000899
YKT6	Putative protein of the vacuolar SNARE complex	42	94	60,6	0,0008954
IRE1	Putative protein kinase	28	85	54,9	0,0008937
PDI1	Putative protein disulfide-isomerase	46	63	40,8	0,0008913
PRE2	Putative proteasome beta-5 subunit	35	130	84,2	0,0008912
orf19.2769	Putative protease B inhibitor	51	92	59,6	0,000891
OFD1	Putative prolyl hydroxylase family member	52	50	32,5	0,0008881
MAS2	Putative processing peptidase, catalytic (alpha) subunit	46	42	27,4	0,0008848
orf19.7041	Putative pre-tRNA processing protein	36	42	27,6	0,0008784
RPF2	Putative pre-rRNA processing protein	61	107	70,5	0,0008761
CDC54	Putative pre-replication complex helicase subunit	46	39	25,7	0,000876
TYR1	Putative prepephenate dehydrogenase	34	44	29	0,0008758
HEM2	Putative porphobilinogen synthase	25	187	124	0,0008705
VTC3	Putative polyphosphate synthetase	44	19	12,6	0,0008704
VTC4	Putative polyphosphate synthetase	36	69	45,8	0,0008696
orf19.7269	Putative polyamine acetyltransferase	41	123	81,7	0,000869
orf19.3037	Putative poly(A)-binding protein	20	28	18,6	0,000869
orf19.6741	Putative plasma membrane protein	33	82	54,5	0,0008685
ADE4	Putative phosphoribosylpyrophosphate amidotransferase	26	34	22,6	0,0008684
orf19.6809	Putative phosphomutase-like protein	35	91	60,6	0,0008668
ERG8	Putative phosphomevalonate kinase	33	33	22	0,0008659
PLB2	Putative phospholipase B	31	24	16	0,0008659
HAL22	Putative phosphoadenosine-5'-phosphate or 3'-phosphoadenosine 5' phosphosulfate phosphatase	39	28	18,7	0,0008643
HAL21	Putative phosphoadenosine-5'-phosphate or 3'-phosphoadenosine 5' phosphosulfate phosphatase	39	100	66,8	0,0008641
CHO1	Putative phosphatidylserine synthase	40	149	99,8	0,0008618
SFH5	Putative phosphatidylinositol transporter	37	182	122,1	0,0008604
orf19.5711	Putative phosphatidylinositol transfer protein	33	89	59,8	0,0008591
CHO2	Putative phosphatidyl-ethanolamine N-methyltransferase	35	161	108,5	0,0008565
orf19.449	Putative phosphatidyl synthase	43	81	54,7	0,0008548
PEX11	Putative peroxisomal membrane protein	29	72	48,8	0,0008517
orf19.2092	Putative peroxisomal cystathionine beta-lyase	40	124	84,2	0,0008501
FOX3	Putative peroxisomal 3-oxoacyl CoA thiolase	36	95	64,6	0,0008489
orf19.5575	Putative peripheral peroxisomal membrane peroxin	34	71	48,3	0,0008485
CYP5	Putative peptidyl-prolyl cis-trans isomerase	31	100	68,1	0,0008476
CPR6	Putative peptidyl-prolyl cis-trans isomerase	30	121	82,5	0,0008466

CPR3	Putative peptidyl-prolyl cis-trans isomerase	35	66	45	0,0008466
orf19.1030	Putative peptidyl-prolyl cis-trans isomerase	32	62	42,3	0,0008461
orf19.2499	Putative peptidyl-prolyl cis-trans isomerase	39	177	121,2	0,000843
RRD1	Putative peptidyl-prolyl cis/trans-isomerase	37	118	81,6	0,0008347
PNG2	Putative peptide:N-glycanase	40	50	34,6	0,0008342
orf19.26	Putative peptide N-glycanase	39	83	57,6	0,0008318
ARO1	Putative pentafunctional arom enzyme	37	88	61,1	0,0008314
ADP1	Putative PDR-subfamily ABC transporter	31	78	54,4	0,0008277
orf19.5095	Putative oxysterol-binding protein	39	37	25,9	0,0008246
orf19.6883	Putative oxysterol binding protein family	27	56	39,2	0,0008246
AYR1	Putative oxidoreductase	47	121	84,9	0,0008227
orf19.5525	Putative oxidoreductase	49	38	26,7	0,0008215
orf19.7307	Putative oxidoreductase	30	35	24,6	0,0008213
orf19.4287	Putative oxidoreductase	47	68	47,9	0,0008195
orf19.3442	Putative oxidoreductase	26	12	8,5	0,0008149
FMA1	Putative oxidoreductase	38	69	48,9	0,0008145
orf19.6463	Putative ortholog of S. cerevisiae Npa3p	35	57	40,6	0,0008104
orf19.2150	Putative ortholog of mammalian electron transfer flavoprotein complex subunit ETF-alpha	35	56	39,9	0,0008102
orf19.3175	Putative ortholog of human electron transfer flavoprotein dehydrogenase (ETF-dH)	37	54	38,5	0,0008096
URA5	Putative orotate phosphoribosyltransferase	36	67	47,9	0,0008074
ARG3	Putative ornithine carbamoyltransferase	31	98	70,1	0,000807
WBP1	Putative oligosaccharyltransferase subunit	28	82	58,7	0,0008064
STT3	Putative oligosaccharyltransferase complex component	36	82	58,9	0,0008036
NOC4	Putative nucleolar protein	48	45	32,5	0,0007993
NOP14	Putative nucleolar protein	27	144	104,1	0,0007985
POM152	Putative nuclear pore membrane glycoprotein	31	78	56,4	0,0007983
GLE2	Putative nuclear pore complex	33	54	39,2	0,0007952
NAB3	Putative nuclear polyadenylated RNA-binding protein	23	30	21,8	0,0007944
NTF2	Putative nuclear envelope protein	34	151	109,9	0,0007931
NAT2	Putative N-terminal acetyltransferase	30	64	46,6	0,0007928
NMD3	Putative nonsense-mediated mRNA decay protein	35	80	58,5	0,0007894
NHP6A	Putative non-histone chromatin component	27	94	68,9	0,0007875
RPN3	Putative non-ATPase regulatory subunit of the 26S proteasome lid	36	52	38,2	0,0007858
NIT3	Putative nitrilase	49	33	24,3	0,0007839
NPT1	Putative nicotinate phosphoribosyltransferase, involved in NAD salvage pathway	22	45	33,2	0,0007824
RSP5	Putative NEDD4 family E3 ubiquitin ligase	45	73	53,9	0,0007818
GDH2	Putative NAD-specific glutamate dehydrogenase	43	34	25,2	0,0007788
orf19.6868	Putative NADPH-dependent methylglyoxal reductase	34	54	40,1	0,0007773
AYR2	Putative NADPH-dependent 1-acyl dihydroxyacetone phosphate reductase	12	22	16,4	0,0007743
orf19.287	Putative NADH-ubiquinone oxidoreductase subunit	49	13	9,7	0,0007736
ALI1	Putative NADH-ubiquinone oxidoreductase	34	34	25,4	0,0007727
orf19.7590	Putative NADH-ubiquinone oxidoreductase	31	44	33	0,0007696
NUC2	Putative NADH-ubiquinone oxidoreductase	36	63	47,3	0,0007688
orf19.2091	Putative NADH dehydrogenase	38	92	69,1	0,0007685
NDE1	Putative NADH dehydrogenase	29	41	30,8	0,0007684
orf19.7092	Putative NAD dependent epimerase/dehydratase family protein	37	70	52,6	0,0007682
TRM1	Putative N2,N2-dimethylguanine tRNA methyltransferase	39	31	23,3	0,000768
MRT4	Putative mRNA turnover protein	27	61	46	0,0007655
MSS51	Putative mRNA maturation factor	33	105	79,3	0,0007643
ELF1	Putative mRNA export protein	30	19	14,4	0,0007616
orf19.325	Putative mRNA cleavage and polyadenlylation factor	36	66	50,1	0,0007604
GLO1	Putative monomeric glyoxalase I	20	68	51,7	0,0007592
MDL1	Putative mitochondrial, half-size MDR-subfamily ABC transporter	44	55	41,9	0,0007577
orf19.5698	Putative mitochondrial ribosomal protein of the large subunit	35	71	54,2	0,0007562
MRPL40	Putative mitochondrial ribosomal protein	34	157	119,9	0,0007558

orf19.498	Putative mitochondrial ribosomal component of the small subunit	34	140	107,1	0,0007546
MRF1	Putative mitochondrial respiratory protein	29	43	33,1	0,0007499
orf19.7459	Putative mitochondrial protein with a predicted role in respiratory growth	29	42	32,4	0,0007483
MIR1	Putative mitochondrial phosphate transporter	32	58	44,8	0,0007473
IDH1	Putative mitochondrial NAD-isocitrate dehydrogenase subunit 1	40	55	42,5	0,000747
IDH2	Putative mitochondrial NAD-isocitrate dehydrogenase subunit	32	135	104,7	0,0007443
OSM2	Putative mitochondrial fumarate reductase	13	29	22,5	0,000744
orf19.2175	Putative mitochondrial cell death effector	44	42	32,6	0,0007437
YHM1	Putative mitochondrial carrier protein	33	326	253,3	0,0007429
AGC1	Putative mitochondrial carrier protein	25	70	54,4	0,0007428
ATP20	Putative mitochondrial ATP synthase	19	14	10,9	0,0007414
MAM33	Putative mitochondrial acidic matrix protein	31	75	58,5	0,00074
orf19.3859	Putative microsomal beta-keto-reductase	29	47	37	0,0007332
CDC48	Putative microsomal ATPase	34	107	84,3	0,0007327
HGT7	Putative MFS glucose transporter	23	80	63,4	0,0007284
MEU1	Putative methylthioadenosine phosphorylase	20	30	23,9	0,0007246
MXR1	Putative methionine sulfoxide reductase	19	33	26,3	0,0007243
MET13	Putative methionine biosynthesis protein	43	73	58,6	0,0007191
orf19.73	Putative metalloprotease	33	58	46,7	0,0007169
orf19.3915	Putative metallodipeptidase	39	69	55,6	0,0007164
orf19.4805	Putative membrane protein	31	57	46	0,0007153
MCM6	Putative MCM DNA replication initiation complex component	44	23	18,7	0,00071
ALG2	Putative mannosyltransferase involved in cell wall mannan biosynthesis	40	94	76,6	0,0007084
RMS1	Putative lysine methyltransferase	30	68	55,6	0,000706
IFA14	Putative LPF family protein	32	79	64,6	0,0007059
orf19.3003	Putative lipid-binding protein with a predicted role in calcium-dependent phospholipid-binding	34	87	71,4	0,0007034
orf19.6869	Putative lipid raft associated protein	25	42	34,5	0,0007027
LKH1	Putative leukotriene A(4) hydrolase	26	81	66,9	0,0006989
HOM3	Putative L-aspartate 4-P-transferase	36	68	56,2	0,0006984
BNA4	Putative kynurenine 3-monooxygenase, involved in NAD biosynthesis	35	39	32,3	0,000697
orf19.394	Putative kynureninase	42	21	17,4	0,0006967
orf19.2489	Putative karyopherin beta	27	45	37,5	0,0006927
ILS1	Putative isoleucyl-tRNA synthetase, the target of drugs including the cyclic beta-amino acid icofungipen/PLD-118/BAY-10-8888 and mupirocin	31	9	7,5	0,0006927
IDP1	Putative isocitrate dehydrogenase	34	29	24,2	0,0006917
orf19.2113	Putative integral peroxisomal membrane protein	30	94	78,6	0,0006903
PRP13	Putative integral inner mitochondrial membrane protein with similarity to exonucleases	37	68	56,9	0,0006898
IPK2	Putative inositol polyphosphate multikinase	49	38	32	0,0006855
ISN1	Putative inosine 5'-monophosphate 5'-nucleotidase	36	290	244,5	0,0006847
IPP1	Putative inorganic pyrophosphatase	29	23	19,4	0,0006844
MTR10	Putative importin	32	29	24,5	0,0006833
HIS7	Putative imidazole glycerol phosphate synthase	25	39	33	0,0006822
HYU1	Putative hydantoin utilization protein A	37	39	33	0,0006822
AHA1	Putative Hsp90p co-chaperone	40	41	34,7	0,000682
HSP70	Putative hsp70 chaperone	52	59	50,2	0,0006784
SSZ1	Putative HSP70 chaperone	39	76	64,9	0,000676
THR1	Putative homoserine kinase	29	23	19,7	0,0006739
HOM6	Putative homoserine dehydrogenase	24	36	30,9	0,0006725
LYS22	Putative homocitrate synthase	29	90	77,3	0,0006721
HGH1	Putative HMG1/2-related protein	25	50	43	0,0006712
HHF1	Putative histone H4	44	13	11,2	0,00067
HTA3	Putative histone H2A	33	50	43,1	0,0006696
HIS5	Putative histidinol-phosphate aminotransferase	39	53	45,7	0,0006694
MAL31	Putative high-affinity maltose transporter	33	104	89,7	0,0006693

CDC46	Putative hexameric MCM complex subunit	48	43	37,1	0,000669
RCF3	Putative heteropentameric replication factor C subunit	32	116	100,3	0,0006676
RFC4	Putative heteropentameric replication factor C subunit	38	63	54,5	0,0006673
HAT2	Putative Hat1-Hat2 histone acetyltransferase complex subunit	40	29	25,1	0,0006669
VCX1	Putative H+/Ca2+ antiporter	22	47	40,7	0,0006666
NHP2	Putative H/ACA snoRNP protein	41	55	47,7	0,0006656
orf19.2965	Putative guanyl-nucleotide exchange factor	35	48	41,8	0,0006629
SEC7	Putative guanine nucleotide exchange factor (GEF)	34	68	59,3	0,0006619
DCK1	Putative guanine nucleotide exchange factor	35	43	37,5	0,0006619
BEM3	Putative GTPase-activating protein (GAP) for Rho-type GTPase Cdc42p	41	62	54,3	0,0006591
RNA1	Putative GTPase-activating protein	45	18	15,8	0,0006576
SEC23	Putative GTPase-activating protein	22	61	53,9	0,0006533
orf19.7149	Putative GTPase inhibitor	30	170	150,4	0,0006525
SAC7	Putative GTPase activating protein (GAP) for Rho1	32	46	40,8	0,0006508
orf19.2917	Putative GTPase	45	28	24,9	0,0006491
orf19.3463	Putative GTPase	33	153	136,3	0,000648
NOG1	Putative GTPase	34	66	58,8	0,0006479
PLB5	Putative GPI-linked phospholipase B, fungal-specific (no mammalian homolog)	20	33	29,4	0,0006479
PGA45	Putative GPI-anchored cell wall protein	28	24	21,4	0,0006474
orf19.2677	Putative GPI transamidase component	43	64	57,1	0,000647
UTR2	Putative GPI anchored cell wall glycosidase	26	54	48,2	0,0006467
GUA1	Putative GMP synthase, involved in the final step of guanine biosynthesis	36	112	100	0,0006465
orf19.1796	Putative glyoxylate reductase	27	62	55,4	0,000646
orf19.338	Putative glycoside hydrolase	22	28	25,1	0,0006439
GPH1	Putative glycogen phosphorylase	23	118	106	0,0006426
GIT2	Putative glycerophosphoinositol permease	38	47	42,3	0,0006414
orf19.4066	Putative glycerol-3-phosphate acyltransferase	39	48	43,3	0,0006399
GUT1	Putative glycerol kinase	30	21	19	0,000638
GSH2	Putative glutathione synthase	38	57	51,8	0,0006352
orf19.86	Putative glutathione peroxidase	41	55	50	0,000635
orf19.4150	Putative glutaredoxin	14	136	123,7	0,0006346
GUS1	Putative glutamine-tRNA ligase	46	34	31	0,0006331
orf19.1460	Putative glutamine-dependent NAD synthetase, involved in NAD salvage pathway	26	32	29,3	0,0006304
GLN1	Putative glutamate synthase	34	76	69,9	0,0006276
GLT1	Putative glutamate synthase	25	90	83	0,0006259
GAD1	Putative glutamate decarboxylase	22	12	11,1	0,000624
GLK1	Putative glucokinase	24	30	27,8	0,0006229
SGA1	Putative glucoamylase	31	51	47,4	0,0006211
orf19.6783	Putative geranylgeranyltransferase regulatory component	31	25	23,3	0,0006194
PRO2	Putative gamma-glutamyl phosphate reductase with a predicted role in proline biosynthesis	26	68	63,4	0,0006191
PRO1	Putative gamma-glutamyl kinase	28	43	40,1	0,000619
GAL7	Putative galactose-1-phoshphate uridyl transferase	19	46	43	0,0006175
UGA1	Putative GABA transaminase	45	43	40,3	0,0006159
ARC1	Putative G4 nucleic acid binding protein	23	91	85,6	0,0006137
orf19.7244	Putative fumarylacetoacetate hydrolase	50	34	32	0,0006133
FUM12	Putative fumarate hydratase	24	36	33,9	0,000613
OSM1	Putative flavoprotein subunit of fumarate reductase	29	24	22,6	0,000613
PST3	Putative flavodoxin	41	56	52,8	0,0006122
YCP4	Putative flavodoxin	46	22	20,9	0,0006076
HEM15	Putative ferrochelatase involved in heme biosynthesis	29	20	19,1	0,0006044
SLC1	Putative fatty acyltransferase	32	29	27,7	0,0006043
ERG9	Putative farnesyl-diphosphate farnesyl transferase (squalene synthase)	27	91	87,1	0,0006031
ERG20	Putative farnesyl pyrophosphate synthetase involved in isoprenoid and sterol biosynthesis, based on similarity to S. cerevisiae Erg20p	31	117	112	0,000603

orf19.4597	Putative F-actin-capping protein subunit beta	18	73	70,1	0,0006011
orf19.3235	Putative F-actin capping protein subunit alpha	45	13	12,5	0,0006003
ATP4	Putative F0-ATP synthase subunit 4	29	61	58,8	0,0005988
ATP5	Putative F0-ATP synthase FO subunit B	42	49	47,3	0,000598
orf19.7153	Putative exportin, member of the Exportin-T family	38	40	38,7	0,0005966
orf19.6596	Putative esterase	20	108	104,8	0,0005949
VPS27	Putative ESCRT-0 complex protein with a role in multivesicular body (MVB) trafficking	24	34	33,2	0,0005911
ERV46	Putative ER-derived vesicle protein	29	71	69,6	0,0005888
ENT3	Putative epsin	27	13	12,8	0,0005863
CDC50	Putative endosomal protein	36	47	46,5	0,0005834
orf19.5486	Putative endoribonuclease	17	26	25,9	0,0005795
SEC62	Putative endoplasmic reticulum (ER) protein-translocation complex subunit	38	41	40,9	0,0005786
CaalfMp11.1	Putative endonuclease encoded by the first exon and part of the first intron of the mitochondrial COB gene	28	32	32	0,0005772
ACF2	Putative endo-1,3-beta-glucanase	40	30	30	0,0005772
orf19.3759	Putative elongator complex subunit	42	19	19	0,0005772
TOM1	Putative E3 ubiquitin ligase	34	21	21,1	0,0005745
DNM1	Putative dynamin-related GTPase	22	101	102	0,0005716
GRE3	Putative D-xylose reductase	43	49	49,5	0,0005714
DPM1	Putative dolichol phosphate mannose synthase	26	50	50,7	0,0005693
orf19.3027	Putative DNA translocase	26	14	14,2	0,0005691
MCM3	Putative DNA replication protein	37	19	19,3	0,0005683
RFA1	Putative DNA replication factor A	27	36	36,6	0,0005678
orf19.843	Putative DNA repair exonuclease	33	36	36,7	0,0005662
MSH2	Putative DNA mismatch repair factor	26	22	22,5	0,0005644
RAD50	Putative DNA double-strand break repair factor	32	21	21,5	0,0005638
DDI1	Putative DNA damage inducible v-SNARE binding protein	54	12	12,3	0,0005632
orf19.5773	Putative dipeptidyl-peptidase III	20	27	27,7	0,0005626
DAK2	Putative dihydroxyacetone kinase	17	11	11,3	0,0005619
KGD2	Putative dihydrolipoamide S-succinyltransferase	29	32	32,9	0,0005614
LPD1	Putative dihydrolipoamide dehydrogenase	35	61	62,8	0,0005607
LAT1	Putative dihydrolipoamide acetyltransferase component (E2) of pyruvate dehydrogenase complex	29	56	57,7	0,0005602
orf19.4609	Putative dienelactone hydrolase	30	62	64	0,0005592
HAM1	Putative deoxyribonucleoside triphosphate pyrophosphohydrolase	26	36	37,3	0,0005571
orf19.2286	Putative deoxyhypusine hydroxylase	38	29	30,1	0,0005561
PUT2	Putative delta-1-pyrroline-5-carboxylate dehydrogenase	34	31	32,2	0,0005557
DBP2	Putative DEAD-box family ATP-dependent RNA helicase	38	39	40,6	0,0005545
IFG3	Putative D-amino acid oxidase	22	119	124	0,000554
CCT3	Putative cytosolic chaperonin Cct ring complex subunit	27	56	58,8	0,0005497
CCT6	Putative cytosolic chaperonin Cct ring complex subunit	26	52	54,6	0,0005497
COX4	Putative cytochrome c oxidase subunit IV	17	63	66,4	0,0005477
COX6	Putative cytochrome c oxidase	14	48	50,6	0,0005476
CTM1	Putative cytochrome c lysine methyltransferase	33	16	16,9	0,0005465
CBR1	Putative cytochrome B5 reductase	29	36	38,1	0,0005454
orf19.5393	Putative cysteine sulfinate decarboxylase	26	44	46,6	0,000545
orf19.7297	Putative cystathionine gamma-synthase	27	32	33,9	0,0005449
orf19.6507	Putative curved DNA-binding protein orthologous to S. pombe Cdb4	27	16	17	0,0005433
SCP1	Putative cortical actin cytoskeleton protein	41	42	44,7	0,0005424
SCO1	Putative copper transporter	36	23	24,5	0,0005419
RPN5	Putative COP9 signalosome component	27	45	48,1	0,00054
COF1	Putative cofilin	33	38	40,7	0,0005389
SGT1	Putative co-chaperone protein with a predicted role in kinetochore assembly	28	119	127,5	0,0005388
CNS1	Putative co-chaperone	35	43	46,2	0,0005373
orf19.3129	Putative chromatin remodelling complex protein	32	42	45,2	0,0005364

ARO2	Putative chorismate synthase	28	41	44,3	0,0005342
ARO7	Putative chorismate mutase	27	58	62,8	0,0005331
PCT1	Putative choline-phosphate cytidylyl transferase, antigenic during human oral infection	38	70	76	0,0005317
CHS5	Putative chitin biosynthesis protein	42	43	46,7	0,0005315
LAG1	Putative ceramide synthase component	31	49	53,4	0,0005297
SUR2	Putative ceramide hydroxylase	26	109	119,1	0,0005283
SCS7	Putative ceramide hydroxylase	29	45	49,3	0,0005269
orf19.7296	Putative cation conductance protein	28	30	32,9	0,0005264
orf19.7140	Putative catechol o-methyltransferase	31	34	37,3	0,0005262
GLC7	Putative catalytic subunit of type 1 serine/threonine protein phosphatase	27	49	53,8	0,0005257
PRC3	Putative carboxypeptidase Y precursor	23	39	42,9	0,0005248
PRC2	Putative carboxypeptidase	31	25	27,5	0,0005248
CPA1	Putative carbamoyl-phosphate synthase subunit	26	15	16,5	0,0005248
CMK2	Putative calmodulin-dependent protein kinase	27	55	60,7	0,000523
ERG5	Putative C-22 sterol desaturase	29	51	56,3	0,0005229
BAT22	Putative branched chain amino acid aminotransferase	23	49	54,2	0,0005219
BAT21	Putative branched chain amino acid aminotransferase	37	31	34,3	0,0005217
TRP3	Putative bifunctional enzyme with predicted indole-3-glycerol-phosphate synthase and anthranilate synthase activities	41	55	60,9	0,0005213
URA2	Putative bifunctional carbamoylphosphate synthetase-aspartate transcarbamylase	34	23	25,5	0,0005206
BMT8	Putative beta-mannosyltransferase, member of a 9-gene family including characterized BMT genes with roles in beta-1,2-mannosylation of cell wall phosphopeptidomannan	21	87	96,5	0,0005204
PRE3	Putative beta-1 proteasome subunit	23	29	32,4	0,0005167
PRE1	Putative beta 4 subunit of the 20S proteasome	16	17	19	0,0005165
PUP3	Putative beta 3 subunit of the 20S proteasome	25	109	121,9	0,0005162
PUP1	Putative beta 2 subunit of the 20S proteasome	27	52	58,2	0,0005157
orf19.239	Putative ATP-dependent helicase	33	82	91,8	0,0005156
DBP3	Putative ATP-dependent DEAD-box RNA helicase	32	20	22,4	0,0005154
RPT6	Putative ATPase of the 19S regulatory particle of the 26S proteasome	36	63	70,6	0,0005151
RPT2	Putative ATPase of the 19S regulatory particle of the 26S proteasome	39	28	31,4	0,0005147
orf19.4953	Putative ATPase	23	73	81,9	0,0005145
APA2	Putative ATP adenylyltransferase II	30	9	10,1	0,0005144
orf19.2335	Putative aspartyl aminopeptidase	23	55	62,1	0,0005112
YPS7	Putative aspartic-type endopeptidase with limited ability to degrade alpha pheromone	23	147	166,1	0,0005109
AAT21	Putative aspartate aminotransferase	31	36	40,7	0,0005106
ASN1	Putative asparagine synthetase	25	19	21,5	0,0005101
orf19.5809	Putative arylformamidase, enzyme of the NAD biosynthesis pathway	29	12	13,6	0,0005093
ARC19	Putative ARP2/3 complex subunit	30	28	31,8	0,0005083
CPA2	Putative arginine-specific carbamoylphosphate synthetase	26	30	34,1	0,0005078
GLO3	Putative ARF GTPase activator	29	106	120,5	0,0005078
GEA2	Putative ARF GTP/GDP exchange factor	28	54	61,4	0,0005077
TRP2	Putative anthranilate synthase with a predicted role in tryptophan biosynthesis	35	34	38,7	0,0005071
orf19.891	Putative AMP deaminase	22	17	19,4	0,0005058
FRP3	Putative ammonium transporter	24	26	29,8	0,0005036
orf19.2397.3	Putative aminotransferase	24	75	86	0,0005034
LAP41	Putative aminopeptidase yscI precursor	27	17	19,5	0,0005032
LAP3	Putative aminopeptidase	26	63	72,4	0,0005023
GAP4	Putative amino acid permease	29	139	160	0,0005015
AMS1	Putative alpha-mannosidase	43	29	33,4	0,0005012
LEU42	Putative alpha-isopropylmalate synthase	28	27	31,1	0,0005011
PRE8	Putative alpha-2_sc subunit of proteasome	25	32	36,9	0,0005006

MNN26	Putative alpha-1,2-mannosyltransferase	28	68	78,9	0,0004975
CKA1	Putative alpha subunit (catalytic subunit) of protein kinase CK2	16	34	39,5	0,0004969
IFE2	Putative alcohol dehydrogenase	30	115	134,4	0,0004939
ALT1	Putative alanine transaminase	28	32	37,4	0,0004939
AGE3	Putative ADP-ribosylation factor GTPase activating protein, functional ortholog of S. cerevisiae GCS1	20	52	61	0,0004921
ARF2	Putative ADP-ribosylation factor	22	68	79,9	0,0004913
orf19.3594	Putative adhesin-like protein	22	32	37,7	0,00049
MET14	Putative adenylylsulfate kinase	27	57	67,2	0,0004896
ADK1	Putative adenylate kinase	19	64	75,5	0,0004893
EHT1	Putative acyl-coenzymeA:ethanol O-acyltransferase	20	16	18,9	0,0004887
AIP2	Putative actin interacting protein	23	34	40,2	0,0004882
orf19.4395	Putative actin cytoskeleton component	20	46	54,5	0,0004872
ACO2	Putative aconitate hydratase 2	23	80	95,1	0,0004856
ARG8	Putative acetylornithine aminotransferase	30	33	39,3	0,0004847
ACC1	Putative acetyl-coenzyme-A carboxylases	31	23	27,4	0,0004845
ILV2	Putative acetolactate synthase	23	24	28,6	0,0004844
PWP2	Putative 90S pre-ribosomal component	26	38	45,4	0,0004831
SOL3	Putative 6-phosphogluconolactonase	25	30	35,9	0,0004824
NSA1	Putative 66S pre-ribosomal particles conmponent	22	34	40,7	0,0004822
RPL42	Putative 60S ribosomal subunit protein	20	20	24	0,000481
RPL2	Putative 60S ribosomal protein L2	21	48	57,7	0,0004802
PRS5	Putative 5-phospho-ribosyl-1(alpha)-pyrophosphate synthetase	29	39	46,9	0,00048
RPS16A	Putative 40S ribosomal subunit	27	88	106	0,0004792
orf19.2269	Putative 3-phosphoserine phosphatase	28	16	19,3	0,0004785
SER1	Putative 3-phosphoserine aminotransferase	20	37	44,7	0,0004778
MET16	Putative 3'-phosphoadenylsulfate reductase	35	42	50,8	0,0004772
orf19.3515	Putative 3-hydroxyanthranilic acid dioxygenase, involved in NAD biosynthesis	21	20	24,2	0,0004771
KGD1	Putative 2-oxoglutarate dehydrogenase	26	59	71,4	0,000477
LEU4	Putative 2-isopropylmalate synthase	34	19	23,1	0,0004748
DOG1	Putative 2-deoxyglucose-6-phosphatase	14	48	58,5	0,0004736
orf19.1180	Putative 2-aminoadipate transaminase	33	57	69,5	0,0004734
RPN6	Putative 26S proteasome subunit	32	32	39,1	0,0004724
RPN2	Putative 26S proteasome subunit	21	45	55,3	0,0004697
RPT1	Putative 26S proteasome regulatory subunit 7	23	59	72,8	0,0004678
orf19.2278	Putative 20S proteasome assembly protein	25	73	90,2	0,0004672
RPN1	Putative 19S regulatory particle of the 26S proteasome	31	37	45,8	0,0004663
GLC3	Putative 1,4-glucan branching enzyme	23	25	31	0,0004655
PST1	Putative 1,4-benzoquinone reductase	24	22	27,3	0,0004652
PNP1	Purine nucleoside phosphorylase	20	11	13,7	0,0004635
SPF1	P-type calcium-transporting ATPase, involved in control of calcium homeostasis, response to ER stress, hyphal growth, biofilm formation and virulence	21	50	62,4	0,0004625
RBT4	Pry family protein	28	69	86,2	0,0004621
orf19.6200	Pry family pathogenesis-related protein	22	16	20	0,0004618
RBE1	Pry family cell wall protein	31	16	20,1	0,0004595
orf19.4246	Protein with similarity to S. cerevisiae Ykr070w	32	57	72,1	0,0004563
HNT1	Protein with similarity to protein kinase C inhibitor-I	22	87	110,2	0,0004557
PR26	Protein with similarity to proteasomal 26S regulatory subunit of S. cerevisiae, H. sapiens, Methanobacterium thermoautotrophicum (Archaebacterium)	16	10	12,7	0,0004545
PRN4	Protein with similarity to pirins	24	27	34,3	0,0004544
HOC1	Protein with similarity to mannosyltransferases	28	38	48,3	0,0004541
orf19.6701	Protein with similarity to amino acid-tRNA ligase	23	33	42	0,0004535
SSD1	Protein with role in resistance to host antimicrobial peptides	31	50	64,3	0,0004489
ZPR1	Protein with putative zinc finger	19	11	14,2	0,0004472
DCW1	Protein with predicted GPI modification	19	35	45,2	0,000447
ARP3	Protein with Myo5p-dependent localization to cortical actin patches at hyphal tip	25	71	91,9	0,000446

orf19.5669	Protein with B-cell receptor-associated protein 31-like domain	28	41	53,2	0,0004449
orf19.597	Protein with an aspartate aminotransferase domain	33	13	16,9	0,000444
orf19.3737	Protein with a Vps9 vacuolar protein sorting protein domain	18	47	61,2	0,0004433
orf19.7328	Protein with a Staphylococcal nuclease domain	26	40	52,2	0,0004423
orf19.3977	Protein with a role in translation	35	64	83,6	0,0004419
SSH1	Protein with a role in protein translocation across membranes	10	54	70,7	0,0004409
PHO88	Protein with a role in phosphate transport	24	25	32,8	0,00044
orf19.4228	Protein with a role in insertion of tail-anchored proteins into the ER membrane	13	117	154,3	0,0004377
orf19.3183	Protein with a role in insertion of tail-anchored proteins into the ER membrane	14	13	17,2	0,0004363
EMP70	Protein with a role in endosome-to-vacuole sorting	34	21	27,8	0,000436
orf19.7310	Protein with a role in directing meiotic recombination events to homologous chromatids	27	18	23,9	0,0004347
SKN1	Protein with a role in beta-1,6-glucan synthesis	24	25	33,3	0,0004334
orf19.3983	Protein with a predicted transcription factor BTF3 domain	17	38	50,7	0,0004326
orf19.3778	Protein with a predicted role in ribosome biogenesis	26	29	38,7	0,0004326
ERB1	Protein with a predicted role in ribosomal large subunit biogenesis	32	24	32,1	0,0004316
orf19.2533.1	Protein with a predicted role in protein translocation from the endoplasmic reticulum	17	18	24,2	0,0004293
SRO77	Protein with a predicted role in docking and fusion of post-Golgi vesicles with the plasma membrane	23	38	51,1	0,0004293
COQ3	Protein with a predicted role in coenzyme Q biosynthesis	26	42	56,7	0,0004276
RAX1	Protein with a predicted role in bud site selection	19	19	25,7	0,0004268
orf19.2487	Protein with a predicted phosphoribulokinase/uridine kinase domain	23	102	138,1	0,0004263
SEC24	Protein with a possible role in ER to Golgi transport	19	22	29,8	0,0004261
orf19.1785	Protein with a PI31 proteasome regulator domain	15	17	23,2	0,000423
orf19.4476	Protein with a NADP-dependent oxidoreductase domain	27	35	47,8	0,0004227
STI1	Protein that interacts with Cdc37 and Crk1 in two-hybrid	29	9	12,3	0,0004224
SLP2	Protein similar to stomatin mechanoreception protein	24	26	35,6	0,0004216
ERG4	Protein similar to sterol C-24 reductase	24	27	37	0,0004212
YTM1	Protein similar to S. cerevisiae Ytm1p, which is involved in biogenesis of the large ribosomal subunit	30	58	79,7	0,0004201
VID27	Protein similar to S. cerevisiae Vid27p	13	28	38,5	0,0004198
orf19.6411	Protein similar to S. cerevisiae Vac14p	31	47	64,7	0,0004193
SPT5	Protein similar to S. cerevisiae Spt5p transcription elongation factor	35	26	35,8	0,0004192
SPL1	Protein similar to S. cerevisiae Spl1p, which is involved in tRNA splicing	21	123	169,4	0,0004191
SIN3	Protein similar to S. cerevisiae Sin3p (transcriptional corepressor involved in histone deacetylase recruitment)	26	16	22,2	0,000416
SDS24	Protein similar to S. cerevisiae Sds24 involved in cell separation during budding	17	36	50,3	0,0004131
ROD1	Protein similar to S. cerevisiae Rod1	19	39	54,6	0,0004123
PEP8	Protein similar to S. cerevisiae Pep8p, which is involved in retrograde transport	33	15	21	0,0004123
orf19.7020	Protein similar to S. cerevisiae Kex1p, which is a pheromone-processing peptidase	26	28	39,3	0,0004113
LHS1	Protein similar to S. cerevisiae Hsp70p	22	28	39,6	0,0004081
GYP7	Protein similar to S. cerevisiae Gyp7p (GTPase-activating protein for Ypt1p)	21	66	93,4	0,0004079
orf19.2304	Protein similar to S. cerevisiae Gvp36p	17	33	46,7	0,0004079
EMP46	Protein similar to S. cerevisiae Emp46, an integral membrane component of ER-derived COPII-coated vesicles	25	110	155,7	0,0004078
DSL1	Protein similar to S. cerevisiae Dsl1p, which is a member of the t-SNARE complex of the endoplasmic reticulum	30	43	60,9	0,0004076
CDC39	Protein similar to S. cerevisiae Cdc39p, which is part of the CCR4-NOT transcription regulatory complex	30	32	45,7	0,0004042

BUB3	Protein similar to S. cerevisiae Bub3	28	21	30,1	0,0004027
ASF1	Protein similar to S. cerevisiae Asf1p, a chromatin assembly complex component	16	35	50,2	0,0004025
ARP9	Protein similar to S. cerevisiae Arp3p, a component of the Arp2/3 complex involved in actin-dependent processes	25	30	43,1	0,0004018
ARC40	Protein similar to S. cerevisiae Arc40	20	43	61,9	0,000401
orf19.2262	Protein similar to quinone oxidoreductases	27	47	67,9	0,0003996
YIM1	Protein similar to protease of mitochondrial inner membrane	30	17	24,8	0,0003957
TIP120	Protein similar to human CAND1 (Cullin-Associated Nedd8-Dissociated) protein involved in regulation of SCF complexes	17	40	58,4	0,0003954
GRP1	Protein similar to dihydroflavonol-4-reductases	27	23	33,6	0,0003951
orf19.7152	Protein similar to Aspergillus CYSK O-acetylserine sulfhydrylase, suggesting that C. albicans uses an O-acetyl-serine (OAS) pathway of sulfur assimilation	36	16	23,4	0,0003947
TOS1	Protein similar to alpha agglutinin anchor subunit	25	36	52,7	0,0003943
AMO2	Protein similar to A. niger predicted peroxisomal copper amino oxidase	19	14	20,5	0,0003942
CTA3	Protein similar to <i>S. cerevisiae</i> Ede1p, which is involved in endocytosis	32	36	52,8	0,0003936
BZZ1	Protein similar to <i>S. cerevisiae</i> Bzz1p, which is an SH3 domain protein involved in the regulation of actin polymerization	16	16	23,5	0,000393
MEA1	Protein similar to <i>A. nidulans</i> MesA, which is involved in localization of actin cables	34	13	19,1	0,0003929
NAG6	Protein required for wild-type mouse virulence and wild-type cycloheximide resistance	22	30	44,1	0,0003927
OSH3	Protein required for wild-type filamentation	11	20	29,4	0,0003927
BEM1	Protein required for wild-type budding, hyphal growth, and virulence in a mouse systemic infection	29	47	69,2	0,0003921
BUD6	Protein required for Spitzenkorper formation in hyphal cells (wild-type localization of Mlc1p to the Spitzenkorper)	23	55	81,2	0,000391
YPT31	Protein required for resistance to toxic ergosterol analog	24	35	51,8	0,00039
ENP1	Protein required for pre-rRNA processing and 40S ribosomal subunit synthesis	23	25	37,1	0,000389
SUR7	Protein required for normal cell wall, plasma membrane, cytoskeletal organization, endocytosis	25	60	89,1	0,0003887
RVS161	Protein required for endocytosis	20	25	37,2	0,0003879
SLA1	Protein required for assembly of the cortical actin cytoskeleton	33	70	104,3	0,0003874
PSP1	Protein repressed during the mating process	21	126	188,2	0,0003865
orf19.4639	Protein present in exponential and stationary growth phase yeast cultures	13	42	62,9	0,0003854
PPZ1	Protein phosphatase Z	35	27	40,6	0,0003839
PTC2	Protein phosphatase of the Type 2C-related family (serine/threonine-specific) with a potential role in DNA damage checkpoint control	27	15	22,6	0,0003831
orf19.3698	Protein of unknown function that may function in RNA processing	22	14	21,1	0,000383
orf19.3354	Protein of unknown function	26	80	120,8	0,0003823
orf19.7502	Protein of unknown function	24	16	24,2	0,0003816
orf19.5660.1	Protein of unknown function	20	33	50	0,000381
orf19.3799	Protein of unknown function	20	12	18,2	0,0003806
orf19.6415.1	Protein of unknown function	39	16	24,3	0,0003801
orf19.3226	Protein of unknown function	24	102	155,2	0,0003794
TOM70	Protein of unknown function	21	86	131,5	0,0003775
orf19.7202	Protein of unknown function	30	24	36,8	0,0003765
PBR1	Protein of unknown function	19	46	70,6	0,0003761
orf19.6810	Protein of unknown function	27	37	56,8	0,000376
SEC21	Protein of unknown function	18	22	33,8	0,0003757
orf19.7322	Protein of unknown function	17	38	58,7	0,0003737
orf19.4633	Protein of unknown function	12	24	37,1	0,0003734
orf19.1448.1	Protein of unknown function	13	21	32,5	0,000373
DBP5	Protein of unknown function	17	16	24,8	0,0003724

orf19.4952.1	Protein of unknown function	17	16	24,8	0,0003724
MDG1	Protein of unknown function	18	33	51,2	0,000372
orf19.4382	Protein of unknown function	29	37	57,5	0,0003714
orf19.1993	Protein of unknown function	18	30	46,8	0,00037
orf19.3558	Protein of unknown function	17	22	34,4	0,0003692
orf19.2757	Protein of unknown function	19	20	31,3	0,0003688
orf19.7531	Protein of unknown function	17	46	72,2	0,0003678
UBC4	Ortholog(s) have proteasome binding, protein-macromolecule adaptor activity, ubiquitin binding, ubiquitin conjugating enzyme activity, ubiquitin-protein transferase activity	21	27	42,5	0,0003667
orf19.1082.1	Protein of unknown function	13	31	48,9	0,0003659
orf19.357	Protein of unknown function	15	11	17,4	0,0003649
orf19.1267	Protein of unknown function	18	24	38	0,0003646
ECM14	Protein of unknown function	22	40	63,4	0,0003642
orf19.3173	Protein of unknown function	20	22	34,9	0,0003639
orf19.5342	Protein of unknown function	26	39	62,1	0,0003625
IDI1	Protein of unknown function	27	33	52,6	0,0003621
orf19.6264.3	Protein of unknown function	24	36	57,4	0,000362
orf19.2518	Protein of unknown function	29	45	71,8	0,0003618
orf19.3910	Protein of unknown function	18	34	54,3	0,0003614
RBK1	Protein of unknown function	21	30	48	0,0003608
LHP1	Protein of unknown function	22	21	33,6	0,0003608
orf19.7578	Protein of unknown function	23	19	30,4	0,0003608
SEC22	Protein of unknown function	25	16	25,6	0,0003608
SEC27	Protein of unknown function	16	24	38,5	0,0003598
orf19.7357	Protein of unknown function	26	36	57,8	0,0003595
orf19.1124.2	Protein of unknown function	17	37	59,6	0,0003584
orf19.2954	Protein of unknown function	22	64	103,1	0,0003583
orf19.933	Protein of unknown function	26	47	75,8	0,0003579
orf19.5006.1	Protein of unknown function	24	49	79,1	0,0003576
orf19.4517	Protein of unknown function	32	19	30,7	0,0003572
orf19.1372	Protein of unknown function	10	18	29,1	0,0003571
orf19.3518	Protein of unknown function	18	75	121,3	0,0003569
orf19.3286	Protein of unknown function	22	9	14,6	0,0003558
orf19.5553	Protein of unknown function	23	41	66,7	0,0003548
orf19.1229	Protein of unknown function	24	78	127	0,0003545
orf19.1940	Protein of unknown function	17	34	55,4	0,0003543
orf19.6484	Protein of unknown function	23	26	42,4	0,000354
orf19.3681	Protein of unknown function	22	104	169,8	0,0003535
orf19.4726	Protein of unknown function	22	56	91,5	0,0003533
orf19.3649	Protein of unknown function	26	29	47,5	0,0003524
orf19.5085	Protein of unknown function	19	18	29,5	0,0003522
orf19.3259	Protein of unknown function	37	23	37,7	0,0003522
orf19.7617	Protein of unknown function	10	32	52,5	0,0003518
ERG12	Protein of unknown function	20	22	36,1	0,0003518
orf19.2168.3	Protein of unknown function	24	41	67,3	0,0003517
orf19.374	Protein of unknown function	18	86	141,2	0,0003516
orf19.2755	Protein of unknown function	21	27	44,4	0,000351
BUD7	Protein of unknown function	20	19	31,3	0,0003504
orf19.131.2	Protein of unknown function	15	21	34,7	0,0003493
orf19.1376	Protein of unknown function	17	36	59,6	0,0003487
COQ6	Protein of unknown function	28	16	26,5	0,0003485
orf19.6607	Protein of unknown function	16	69	114,4	0,0003482
orf19.6602	Protein of unknown function	19	27	44,8	0,0003479
orf19.213	Protein of unknown function	18	33	54,8	0,0003476
orf19.4830	Protein of unknown function	25	21	34,9	0,0003473
orf19.2460	Protein of unknown function	28	25	41,6	0,0003469
orf19.5411	Protein of unknown function	22	27	45	0,0003463
orf19.51	Protein of unknown function	21	23	38,5	0,0003448
orf19.5682	Protein of unknown function	21	24	40,2	0,0003446

BET4	Protein of unknown function	19	15	25,2	0,0003436
orf19.518	Protein of unknown function	24	20	33,7	0,0003426
orf19.4283	Protein of unknown function	21	35	59	0,0003424
orf19.3516	Protein of unknown function	25	14	23,6	0,0003424
orf19.714	Protein of unknown function	12	32	54,1	0,0003414
VTI1	Protein of unknown function	19	89	150,7	0,0003409
orf19.3843	Protein of unknown function	11	18	30,5	0,0003407
orf19.3128	Protein of unknown function	28	25	42,4	0,0003404
orf19.6424	Protein of unknown function	23	32	54,3	0,0003402
orf19.1514	Protein of unknown function	23	41	69,7	0,0003396
orf19.5293	Protein of unknown function	11	28	47,6	0,0003396
orf19.2095	Protein of unknown function	16	16	27,3	0,0003383
HSM3	Ortholog(s) have role in mismatch repair, proteasome regulatory particle assembly and cytosol, nucleus localization	16	22	37,6	0,0003377
orf19.6082	Protein of unknown function	29	20	34,2	0,0003376
orf19.5834	Protein of unknown function	16	24	41,1	0,0003371
orf19.6822	Protein of unknown function	24	14	24	0,0003367
orf19.1054	Protein of unknown function	20	17	29,2	0,0003361
orf19.1833	Protein of unknown function	20	21	36,1	0,0003358
orf19.4751	Protein of unknown function	12	24	41,4	0,0003346
orf19.3810	Protein of unknown function	19	31	53,5	0,0003345
orf19.3312	Protein of unknown function	21	21	36,3	0,0003339
orf19.164	Protein of unknown function	21	31	53,7	0,0003332
orf19.3583	Protein of unknown function	24	17	29,5	0,0003326
orf19.7085	Protein of unknown function	10	36	62,5	0,0003325
orf19.2452	Protein of unknown function	22	30	52,1	0,0003324
orf19.4030	Protein of unknown function	28	30	52,2	0,0003317
orf19.3053	Protein of unknown function	14	41	71,4	0,0003315
SER2	Protein of unknown function	26	37	64,5	0,0003311
orf19.5925	Protein of unknown function	25	23	40,1	0,0003311
orf19.2265	Protein of unknown function	21	15	26,2	0,0003305
orf19.5833	Protein of unknown function	18	24	42	0,0003298
orf19.5961	Protein of unknown function	24	26	45,6	0,0003291
orf19.3482	Protein of unknown function	15	12	21,1	0,0003283
orf19.730	Protein of unknown function	27	25	44	0,000328
orf19.6748	Protein of unknown function	18	33	58,1	0,0003279
orf19.4865	Protein of unknown function	21	21	37	0,0003276
orf19.4131	Protein of unknown function	35	26	45,9	0,000327
orf19.4611	Protein of unknown function	11	31	55	0,0003254
orf19.5515	Protein of unknown function	15	16	28,4	0,0003252
orf19.6039	Protein of unknown function	19	54	96,2	0,000324
orf19.5278	Protein of unknown function	29	23	41,1	0,000323
IST2	Protein of unknown function	20	39	69,7	0,000323
orf19.1800	Protein of unknown function	31	24	43,1	0,0003214
orf19.5516	Protein of unknown function	16	27	48,5	0,0003213
URA6	Protein of unknown function	22	28	50,4	0,0003207
orf19.1338	Protein of unknown function	25	19	34,2	0,0003207
orf19.6804	Protein of unknown function	12	25	45,1	0,00032
orf19.5547	Protein of unknown function	32	13	23,5	0,0003193
orf19.4850	Protein of unknown function	27	16	29	0,0003185
orf19.2328	Protein of unknown function	16	25	45,4	0,0003179
orf19.4532	Protein of unknown function	15	55	100	0,0003175
NEP1	Protein of unknown function	16	32	58,3	0,0003168
orf19.1495	Protein of unknown function	15	37	67,5	0,0003164
orf19.7086	Protein of unknown function	12	24	43,8	0,0003163
orf19.4731	Protein of unknown function	28	33	60,5	0,0003149
orf19.1545	Protein of unknown function	28	27	49,5	0,0003149
orf19.6627	Protein of unknown function	18	27	49,6	0,0003142
orf19.7378	Protein of unknown function	25	33	60,9	0,0003128
orf19.3351	Protein of unknown function	24	11	20,3	0,0003128

orf19.1769	Protein of unknown function	15	38	70,2	0,0003125
orf19.1086	Protein of unknown function	19	40	73,9	0,0003124
orf19.3755	Protein of unknown function	17	27	49,9	0,0003123
orf19.6076	Protein of unknown function	35	18	33,3	0,000312
orf19.4516	Protein of unknown function	23	21	38,9	0,0003116
orf19.7210	Protein of unknown function	20	17	31,6	0,0003105
orf19.1991	Protein of unknown function	10	27	50,2	0,0003105
orf19.3615	Protein of unknown function	24	21	39,1	0,00031
orf19.6071	Protein of unknown function	26	38	70,8	0,0003098
orf19.4306	Protein of unknown function	22	16	29,9	0,0003089
orf19.7368	Protein of unknown function	20	36	67,3	0,0003088
orf19.2484	Protein of unknown function	18	28	52,4	0,0003084
orf19.5965	Protein of unknown function	28	30	56,4	0,000307
orf19.1359	Protein of unknown function	20	42	79	0,0003069
orf19.4537	Protein of unknown function	30	9	17	0,0003056
orf19.5619	Protein of unknown function	15	19	35,9	0,0003055
orf19.1412	Protein of unknown function	16	23	43,5	0,0003052
orf19.1179	Protein of unknown function	17	32	60,6	0,0003048
CDC55	Protein of unknown function	24	18	34,2	0,0003038
orf19.2520	Protein of unknown function	17	29	55,2	0,0003033
orf19.3920	Protein of unknown function	9	31	59,2	0,0003023
orf19.5857	Protein of unknown function	25	28	53,6	0,0003015
orf19.1272	Protein of unknown function	18	21	40,4	0,0003
orf19.2228	Protein of unknown function	21	27	52,4	0,0002974
orf19.4963	Protein of unknown function	31	22	42,8	0,0002967
MNN11	Protein of unknown function	16	14	27,3	0,000296
NIF3	Protein of unknown function	18	29	56,7	0,0002952
orf19.1527	Protein of unknown function	23	27	52,9	0,0002946
orf19.1012	Protein of unknown function	19	42	82,3	0,0002946
orf19.2664	Protein of unknown function	24	29	57	0,0002937
orf19.7185	Protein of unknown function	25	51	100,3	0,0002935
orf19.3185	Protein of unknown function	12	12	23,6	0,0002935
orf19.5671	Protein of unknown function	9	19	37,4	0,0002932
MNN21	Protein of unknown function	24	27	53,2	0,000293
orf19.1864	Protein of unknown function	14	36	71	0,0002927
orf19.6119	Protein of unknown function	16	49	97,1	0,0002913
orf19.5275	Protein of unknown function	17	35	69,6	0,0002903
orf19.6933	Protein of unknown function	12	18	35,8	0,0002902
orf19.4153	Protein of unknown function	19	28	55,8	0,0002897
orf19.792	Protein of unknown function	22	19	37,9	0,0002894
orf19.1124	Protein of unknown function	14	21	42	0,0002886
SEC10	Protein of unknown function	10	17	34,1	0,0002878
orf19.5322	Protein of unknown function	15	32	64,2	0,0002877
orf19.5321	Protein of unknown function	18	19	38,2	0,0002871
orf19.2835	Protein of unknown function	22	19	38,3	0,0002864
orf19.1697	Protein of unknown function	15	53	106,9	0,0002862
orf19.6739	Protein of unknown function	13	25	50,5	0,0002858
orf19.6693	Protein of unknown function	17	23	46,5	0,0002855
orf19.969	Protein of unknown function	13	18	36,4	0,0002854
orf19.5418	Protein of unknown function	21	7	14,2	0,0002846
orf19.5618	Protein of unknown function	15	8	16,3	0,0002833
orf19.3744	Protein of unknown function	17	35	71,6	0,0002822
orf19.7386	Protein of unknown function	22	15	30,8	0,0002811
orf19.585	Protein of unknown function	22	20	41,1	0,0002809
orf19.5921	Protein of unknown function	26	34	70	0,0002804
orf19.2516	Protein of unknown function	17	17	35	0,0002804
STR2	Protein of unknown function	13	22	45,4	0,0002797
orf19.757	Protein of unknown function	25	6	12,4	0,0002793
orf19.1037	Protein of unknown function	13	16	33,1	0,000279
orf19.1777	Protein of unknown function	26	30	62,2	0,0002784
orf19.7347	Protein of unknown function	22	34	70,7	0,0002776

orf19.1531	Protein of unknown function	19	15	31,2	0,0002775
orf19.4864	Protein of unknown function	17	47	97,8	0,0002774
orf19.1043	Protein of unknown function	13	45	93,9	0,0002766
orf19.3939	Protein of unknown function	25	41	85,9	0,0002755
orf19.3836	Protein of unknown function	14	43	90,2	0,0002752
orf19.6462	Protein of unknown function	17	19	39,9	0,0002749
orf19.3505	Protein of unknown function	24	14	29,4	0,0002749
orf19.6403	Protein of unknown function	28	5	10,5	0,0002749
orf19.185	Protein of unknown function	18	14	29,5	0,0002739
orf19.6752	Protein of unknown function	17	35	74,3	0,0002719
orf19.3367	Protein of unknown function	20	14	29,8	0,0002712
orf19.3135	Protein of unknown function	27	9	19,2	0,0002706
orf19.5817	Protein of unknown function	10	43	91,8	0,0002704
orf19.4621	Protein of unknown function	14	51	108,9	0,0002703
orf19.2301	Protein of unknown function	16	21	45	0,0002694
orf19.2847	Protein of unknown function	20	14	30	0,0002694
orf19.5442	Protein of unknown function	19	18	38,6	0,0002692
orf19.4293	Protein of unknown function	25	8	17,3	0,0002669
orf19.1764	Protein of unknown function	13	12	26	0,0002664
orf19.3686	Protein of unknown function	22	27	58,6	0,000266
orf19.5777	Protein of unknown function	17	39	84,8	0,0002655
orf19.2820	Protein of unknown function	18	29	63,1	0,0002653
orf19.501	Protein of unknown function	21	35	76,5	0,0002641
orf19.6477	Protein of unknown function	21	15	32,8	0,000264
orf19.4122	Protein of unknown function	15	14	30,7	0,0002632
orf19.4029	Protein of unknown function	15	14	30,7	0,0002632
orf19.1433	Protein of unknown function	17	15	32,9	0,0002632
VPS17	Protein of unknown function	14	18	39,5	0,000263
orf19.6245	Protein of unknown function	17	18	39,5	0,000263
orf19.7345	Protein of unknown function	14	10	22	0,0002624
orf19.7444	Protein of unknown function	24	15	33,3	0,00026
orf19.4932	Protein of unknown function	22	30	67	0,0002585
orf19.5564	Protein of unknown function	13	21	46,9	0,0002585
orf19.352	Protein of unknown function	20	32	71,5	0,0002583
orf19.273	Protein of unknown function	15	11	24,6	0,0002581
orf19.5209	Protein of unknown function	23	13	29,1	0,0002579
orf19.3352	Protein of unknown function	15	20	45	0,0002565
orf19.7478	Protein of unknown function	17	14	31,5	0,0002565
orf19.3136	Protein of unknown function	18	23	51,8	0,0002563
orf19.1305	Protein of unknown function	14	28	63,4	0,0002549
orf19.1400	Protein of unknown function	20	59	133,9	0,0002543
orf19.199	Protein of unknown function	19	40	91,4	0,0002526
GCN1	Protein of unknown function	10	18	41,2	0,0002522
orf19.4960	Protein of unknown function	15	11	25,2	0,000252
orf19.2002	Protein of unknown function	19	24	55,1	0,0002514
orf19.6160	Protein of unknown function	18	21	48,3	0,000251
orf19.7012	Protein of unknown function	20	12	27,7	0,0002501
orf19.4705	Protein of unknown function	19	12	27,7	0,0002501
orf19.5160	Protein of unknown function	21	25	57,8	0,0002497
orf19.4172	Protein of unknown function	22	14	32,5	0,0002487
orf19.5229	Protein of unknown function	17	6	14	0,0002474
orf19.7326	Protein of unknown function	24	14	32,7	0,0002471
orf19.4061	Protein of unknown function	16	16	37,4	0,0002469
orf19.6355	Protein of unknown function	8	28	65,5	0,0002468
orf19.1516	Protein of unknown function	24	10	23,4	0,0002467
orf19.2070	Protein of unknown function	24	12	28,1	0,0002465
orf19.3914	Protein of unknown function	22	24	56,4	0,0002456
orf19.1956	Protein of unknown function	23	22	51,7	0,0002456
orf19.2867	Protein of unknown function	19	37	87	0,0002455
orf19.4117	Protein of unknown function	16	100	235,2	0,0002454
UBA2	Protein of unknown function	23	13	30,6	0,0002452

orf19.6020	Protein of unknown function	9	24	56,6	0,0002448
orf19.445	Protein of unknown function	8	20	47,4	0,0002436
orf19.3447	Protein of unknown function	16	52	123,5	0,000243
SEC1	Protein of unknown function	11	29	68,9	0,000243
orf19.5003	Protein of unknown function	21	21	50	0,0002424
orf19.536	Protein of unknown function	15	11	26,2	0,0002424
orf19.7038	Protein of unknown function	18	18	42,9	0,0002422
MNN10	Protein of unknown function	11	20	47,8	0,0002415
orf19.7144	Protein of unknown function	15	36	86,2	0,0002411
orf19.5747	Protein of unknown function	17	28	67,6	0,0002391
orf19.764	Protein of unknown function	10	14	33,8	0,0002391
orf19.5783	Protein of unknown function	17	19	45,9	0,0002389
orf19.6855	Protein of unknown function	14	20	48,4	0,0002385
orf19.3333	Protein of unknown function	10	22	53,3	0,0002383
orf19.6843	Protein of unknown function	21	14	34	0,0002377
orf19.841	Protein of unknown function	10	35	85,1	0,0002374
orf19.4191.1	Protein of unknown function	11	33	80,4	0,0002369
orf19.504	Protein of unknown function	10	8	19,5	0,0002368
orf19.524	Protein of unknown function	12	10	24,4	0,0002366
orf19.851	Protein of unknown function	14	20	48,9	0,0002361
orf19.1994	Protein of unknown function	9	16	39,2	0,0002356
orf19.3260	Protein of unknown function	21	40	98,4	0,0002346
orf19.6418	Protein of unknown function	21	5	12,3	0,0002346
orf19.2796	Protein of unknown function	17	24	59,1	0,0002344
NUP84	Protein of unknown function	27	15	37	0,000234
YME1	Protein of unknown function	15	22	54,3	0,0002339
orf19.4018	Protein of unknown function	20	14	34,6	0,0002336
orf19.5533	Protein of unknown function	12	16	39,7	0,0002326
orf19.6966	Protein of unknown function	19	28	69,6	0,0002322
orf19.1395	Protein of unknown function	21	15	37,3	0,0002321
orf19.4437	Protein of unknown function	15	18	44,8	0,0002319
orf19.2964	Protein of unknown function	15	11	27,4	0,0002317
orf19.7497	Protein of unknown function	16	19	47,4	0,0002314
orf19.7067	Protein of unknown function	14	17	42,6	0,0002304
orf19.3004	Protein of unknown function	19	17	42,9	0,0002287
orf19.3706	Protein of unknown function	11	24	60,6	0,0002286
orf19.6610	Protein of unknown function	13	17	43	0,0002282
orf19.3792	Protein of unknown function	22	26	65,8	0,0002281
orf19.5238	Protein of unknown function	25	18	45,6	0,0002279
orf19.4368	Protein of unknown function	11	18	45,6	0,0002279
orf19.6579	Protein of unknown function	16	41	104,2	0,0002271
orf19.4749	Protein of unknown function	23	19	48,4	0,0002266
orf19.6438	Protein of unknown function	18	44	113	0,0002248
orf19.4127	Protein of unknown function	17	27	69,5	0,0002243
orf19.5799	Protein of unknown function	13	20	51,5	0,0002242
orf19.6233	Protein of unknown function	13	45	116,1	0,0002237
GYP2	Protein of unknown function	11	21	54,2	0,0002237
orf19.879	Protein of unknown function	13	16	41,3	0,0002236
orf19.2068	Protein of unknown function	16	27	69,7	0,0002236
orf19.2041	Protein of unknown function	12	12	31	0,0002234
orf19.2057	Protein of unknown function	10	16	41,4	0,0002231
orf19.4627	Protein of unknown function	20	8	20,7	0,0002231
orf19.1664	Protein of unknown function	9	34	88,3	0,0002223
PEX6	Protein of unknown function	11	7	18,2	0,000222
orf19.4312	Protein of unknown function	14	39	102,2	0,0002203
orf19.1608	Protein of unknown function	18	15	39,4	0,0002198
PCD1	Protein of unknown function	18	13	34,2	0,0002194
orf19.2485	Protein of unknown function	10	21	55,3	0,0002192
orf19.6665	Protein of unknown function	14	9	23,8	0,0002183
MLP1	Protein of unknown function	15	42	111,2	0,000218
orf19.4713	Protein of unknown function	22	34	90,1	0,0002178

orf19.4253	Protein of unknown function	22	34	90,1	0,0002178
orf19.6980	Protein of unknown function	15	35	92,8	0,0002177
orf19.4795	Protein of unknown function	12	29	76,9	0,0002177
orf19.457	Protein of unknown function	10	31	82,9	0,0002159
orf19.52	Protein of unknown function	11	26	69,6	0,0002156
orf19.3552	Protein of unknown function	15	14	37,5	0,0002155
orf19.5009	Protein of unknown function	9	15	40,2	0,0002154
orf19.1191	Protein of unknown function	14	17	45,6	0,0002152
orf19.748	Protein of unknown function	7	28	75,4	0,0002144
orf19.4959	Protein of unknown function	15	30	81,1	0,0002135
orf19.7011	Protein of unknown function	30	17	46,1	0,0002129
orf19.3237	Protein of unknown function	13	15	40,7	0,0002127
HYM1	Protein of RAM cell wall integrity signaling network	21	22	59,9	0,000212
MNN9	Protein of N-linked outer-chain mannan biosynthesis	13	25	68,5	0,0002107
KRE9	Protein of beta-1,6-glucan biosynthesis	16	29	79,6	0,0002103
PMT2	Protein mannosyltransferase (PMT) with roles in hyphal growth and drug sensitivity	11	23	63,4	0,0002094
PMT1	Protein mannosyltransferase	12	17	47	0,0002088
PMT4	Protein mannosyltransferase	18	22	60,9	0,0002085
FGR44	Protein lacking an ortholog in S. cerevisiae	11	12	33,3	0,000208
BCY1	Protein kinase A regulatory subunit	14	21	58,4	0,0002076
LMO1	Protein involved together with Dck1p and Rac1p in invasive filamentous growth and cell wall integrity	9	16	44,5	0,0002075
VAC8	Protein involved in vacuolar inheritance	15	18	50,1	0,0002074
TIM44	Protein involved in transport across membranes	17	21	58,5	0,0002072
RIM20	Protein involved in the pH response pathway	21	32	89,2	0,0002071
BFR1	Protein involved in the maintenance of normal ploidy	15	30	83,7	0,0002069
SSF1	Protein involved in ribosome biogenesis	12	29	81,1	0,0002064
YBP1	Protein involved in response to oxidative stress, binds and stabilizes Cap1p transcription factor in response to H2O2	16	8	22,4	0,0002062
TOM40	Protein involved in mitochondrial protein import	6	12	33,7	0,0002055
GGA2	Protein involved in Golgi trafficking	18	29	81,8	0,0002046
ERP5	Protein involved in ER to Golgi transport	16	25	70,6	0,0002044
orf19.7615	Protein involved in endoplasmic reticulum (ER) to Golgi vesicle-mediated transport	17	23	65	0,0002043
POB3	Protein involved in chromatin assembly and disassembly	19	26	73,5	0,0002042
SPA2	Protein involved in cell polarity, Spitzenkorper formation	10	32	90,5	0,0002041
TIP20	Protein interacting with Sec20p, possibly involved in retrograde transport between the Golgi and the endoplasmic reticulum	14	34	96,2	0,000204
GIG1	Protein induced by N-acetylglucosamine (GlcNAc)	10	10	28,3	0,000204
HRT2	Protein described as having a role in Ty3 transposition	17	26	74,1	0,0002025
RPS21	Protein component of the small (40S) subunit	20	10	28,5	0,0002025
orf19.4149.1	Protein component of the small (40S) ribosomal subunit	11	49	139,8	0,0002023
SCL1	Proteasome subunit YC7alpha	18	22	62,8	0,0002022
PHB2	Prohibitin 2	15	25	71,4	0,0002021
PFY1	Profilin	9	10	28,7	0,0002011
CWH41	Processing alpha glucosidase I, involved in N-linked protein glycosylation and assembly of cell wall beta 1,6 glucan	8	15	43,2	0,0002004
orf19.109	Probable mitochondrial tyrosyl-tRNA synthetase, based on conservation in other fungi	18	33	95,2	0,0002001
STE24	Prenyl-dependent protease	12	14	40,4	0,0002
orf19.6707	Predicted vacuolar protein with a calcineurin-like phosphoesterase domain	12	24	69,3	0,0001999
orf19.2114	Predicted uricase	12	14	40,6	0,000199
UBC8	Predicted ubiquitin-conjugating enzyme that negatively regulates gluconeogenesis by mediating the glucose-induced ubiquitination of fructose-1,6-bisphosphatase	6	15	43,6	0,0001986
TRP5	Predicted tryptophan synthase	11	10	29,1	0,0001984
TPS3	Predicted trehalose-phosphate synthase regulatory subunit	13	19	55,3	0,0001983
orf19.2930	Predicted translation initiation factor	11	45	131,4	0,0001977

UGA2	Predicted succinate semialdehyde dehydrogenase	14	10	29,2	0,0001977
SEC6	Predicted subunit of the exocyst complex, involved in exocytosis	19	9	26,3	0,0001975
EXO70	Predicted subunit of the exocyst complex, involved in exocytosis	11	9	26,3	0,0001975
SEC8	Predicted subunit of the exocyst complex, involved in exocytosis	14	11	32,2	0,0001972
EXO84	Predicted subunit of the exocyst complex, involved in exocytosis	19	9	26,4	0,0001968
STV1	Predicted subunit a of vacuolar proton-translocating ATPase V0 domain, Golgi isoform	17	19	55,8	0,0001965
orf19.320	Predicted short chain dehydrogenase	16	18	53	0,000196
orf19.3932	Predicted RNA binding protein	12	15	44,4	0,000195
RPS18	Predicted ribosomal protein	11	13	38,5	0,0001949
RPL10A	Predicted ribosomal protein	16	19	56,3	0,0001948
RPS9B	Predicted ribosomal protein	13	17	50,5	0,0001943
RPS22A	Predicted ribosomal protein	16	14	41,6	0,0001943
RPS24	Predicted ribosomal protein	19	25	74,8	0,0001929
RPL24A	Predicted ribosomal protein	12	28	84,1	0,0001922
RPL18	Predicted ribosomal protein	11	11	33,1	0,0001918
ENA21	Predicted P-type ATPase sodium pump	16	11	33,2	0,0001913
VMA13	Predicted proton-transporting ATPase	13	21	63,5	0,0001909
orf19.577	Predicted protein tyrosine phosphatase	11	25	75,8	0,0001904
HRR25	Predicted protein serine/threonine kinase	15	8	24,3	0,00019
orf19.1953	Predicted protein of unknown function	13	13	39,5	0,00019
orf19.4488	Predicted ortholog of S. cerevisiae Swi3, subunit of the SWI/SNF chromatin remodeling complex	10	9	27,4	0,0001896
orf19.1761	Predicted olichyl-diphosphooligosaccharide-protein glycotransferase	18	15	45,7	0,0001895
orf19.1049	Predicted NUDIX hydrolase domain	17	22	67,1	0,0001893
MIS11	Predicted mitochondrial C1-tetrahydrofolate synthase precursor	18	15	45,8	0,0001891
MDH1-1	Predicted malate dehydrogenase precursor	13	42	128,6	0,0001885
orf19.1840	Predicted lipid-binding ER protein	13	32	98	0,0001885
MSN5	Predicted karyopherin involved in nuclear import and export of proteins	8	29	89	0,0001881
orf19.1052	Predicted histone H2B	10	23	71,3	0,0001862
orf19.2426	Predicted HD domain metal dependent phosphohydrolase	9	13	40,5	0,0001853
APL4	Predicted gamma-adaptin, large subunit of the clathrin-associated protein (AP-1) complex	23	11	34,3	0,0001851
SEC15	Predicted exocyst subunit	14	12	37,5	0,0001847
SEC5	Predicted exocyst component	12	22	68,8	0,0001846
orf19.5281	Predicted essential RNA-binding G protein	15	14	43,8	0,0001845
orf19.2257	Predicted ER protein involved in ER-nucleus signaling	14	51	159,7	0,0001843
SER33	Predicted enzyme of amino acid biosynthesis	20	9	28,2	0,0001842
TRP4	Predicted enzyme of amino acid biosynthesis	11	18	56,6	0,0001836
orf19.2036	Predicted dihydrodiol dehydrogenase	8	10	31,5	0,0001832
TIM50	Predicted component of the Translocase of the Inner Mitochondrial membrane (TIM23 complex)	15	29	91,5	0,0001829
PAM17	Predicted component of the presequence translocase-associated import motor (PAM complex) involved in protein import into mitochondrial matrix	14	30	94,7	0,0001829
YHM2	Predicted carrier protein	9	7	22,1	0,0001828
DED1	Predicted ATP-dependent RNA helicase	16	11	34,8	0,0001825
orf19.2988	Predicted aminotransferase based on S. pombe ortholog SPBC660.12c	12	9	28,5	0,0001823
orf19.3045	Predicted aldehyde dehydrogenase domain	8	11	34,9	0,0001819
orf19.2124	Predicted alcohol dehydrogenase	20	7	22,3	0,0001812
orf19.5239	Predicted alanine-tRNA ligase	12	56	178,6	0,000181
SSU81	Predicted adaptor protein involved in activation of MAP kinase-dependent signaling pathways	16	14	44,8	0,0001804
POX1-3	Predicted acyl-CoA oxidase	20	8	25,7	0,0001797
FAA4	Predicted acyl CoA synthase	16	46	148,3	0,000179
orf19.1533	Possible vacuolar protein	10	11	35,5	0,0001789
CIP1	Possible oxidoreductase	16	13	42	0,0001787

YBN5	P-loop ATPase with similarity to human OLA1 and bacterial YchF	16	23	74,8	0,0001775
orf19.1564	Plasma membrane-localized protein of unknown function	18	9	29,4	0,0001767
orf19.1549	Plasma membrane-associated protein identified in detergent-resistant membrane fraction (possible lipid raft component)	16	21	68,9	0,0001759
orf19.3430	Plasma membrane-associated protein	14	36	118,4	0,0001755
YCK2	Plasma membrane protein similar to S. cerevisiae casein kinase I, Yck2p	13	8	26,4	0,0001749
orf19.3335	Plasma membrane protein of unknown function	22	31	102,4	0,0001747
RAX2	Plasma membrane protein involved in establishment of bud sites and linear direction of hyphal growth	10	14	46,3	0,0001745
PMA1	Plasma membrane H(+)-ATPase	14	9	29,9	0,0001738
APL2	Phosphorylated protein of unknown function	17	27	90,2	0,0001728
MCM2	Phosphorylated protein of unknown function	11	14	47	0,0001719
orf19.1204	Phosphorylated protein of unknown function	13	15	50,8	0,0001704
CDC47	Phosphorylated protein described as having role in control of cell division	11	24	81,3	0,0001704
PRS1	Phosphoribosylpyrophosphate synthetase	12	7	23,8	0,0001698
ADE1	Phosphoribosylaminoimadazole succinocarboxamide synthetase, enzyme of adenine biosynthesis	12	20	68,2	0,0001693
ADE2	Phosphoribosylaminoimadazole carboxylase	13	12	41	0,0001689
ADE5,7	Phosphoribosylamine-glycine ligase and phosphoribosylformylglycinamidine cyclo-ligase	15	8	27,4	0,0001685
PMI1	Phosphomannose isomerase	14	8	27,4	0,0001685
PMM1	Phosphomannomutase	12	10	34,3	0,0001683
PLB4.5	Phospholipase B	14	22	75,8	0,0001675
GPM1	Phosphoglycerate mutase	5	16	55,3	0,000167
PGK1	Phosphoglycerate kinase	9	20	69,3	0,0001666
PFK2	Phosphofructokinase beta subunit	10	14	48,6	0,0001663
PFK1	Phosphofructokinase alpha subinit	9	9	31,4	0,0001654
PCK1	Phosphoenolpyruvate carboxykinase	18	29	101,2	0,0001654
AGM1	Phosphoacetylglucosamine mutase (N-acetylglucosamine-phosphate mutase)	13	8	28	0,0001649
MSS4	Phosphatidylinositol-4-phosphate 5-kinase	13	20	70,2	0,0001645
PDR16	Phosphatidylinositol transfer protein	16	10	35,1	0,0001645
PLC2	Phosphatidylinositol (PtdIns)-specific phospholipase C (PI-PLC)	18	26	91,5	0,000164
FRS1	Phenylalanyl-tRNA synthetase	8	14	49,3	0,0001639
CYP1	Peptidyl-prolyl cis-trans isomerase	7	12	42,5	0,000163
SAP7	Pepstatin A-insensitive secreted aspartyl protease	12	22	78,2	0,0001624
PUF3	Ortholog os S. cerevisiae Puf3	10	11	39,2	0,000162
orf19.5368	Ortholog of Vms1	11	15	53,5	0,0001618
IPK1	Ortholog of S. cerevisiae/S. pombe Ipk1	14	33	118,2	0,0001612
APM1	Ortholog of S. cerevisiae/S. pombe Apm1	15	6	21,5	0,0001611
ZUO1	Ortholog of S. cerevisiae Zuo1	21	22	79,3	0,0001601
orf19.592	Ortholog of S. cerevisiae YNL092W and S. pombe SPBC1778.07	6	39	141	0,0001597
STE23	Ortholog of S. cerevisiae Ste23 metalloprotease	9	11	39,9	0,0001591
orf19.3219	Ortholog of S. cerevisiae Sia1	11	32	116,1	0,0001591
orf19.3854	Ortholog of S. cerevisiae Sat4	12	6	21,8	0,0001589
RPL6	Ortholog of S. cerevisiae ribosomal subunit, Rpl6B	11	17	61,9	0,0001585
orf19.1687	Ortholog of S. cerevisiae Prp43, an RNA helicase in the DEAH-box family that functions in both RNA polymerase I and polymerase II transcript metabolism	10	12	43,8	0,0001581
PGM2	Ortholog of S. cerevisiae Pgm2	8	22	80,4	0,000158
orf19.6604	Ortholog of S. cerevisiae Pba1 that is involved in 20S proteasome assembly	14	35	128	0,0001578
NUP85	Ortholog of S. cerevisiae Nup85	15	32	117,8	0,0001568
NOP5	Ortholog of S. cerevisiae Nop58	7	36	132,8	0,0001565
orf19.2414	Ortholog of S. cerevisiae Mpm1	18	8	29,7	0,0001555
orf19.512	Ortholog of S. cerevisiae Kre33	12	13	48,6	0,0001544
HRT1	Ortholog of S. cerevisiae Hrt1. Component of a nuclear ubiquitin-protein ligase complex involved in cell cycle control	14	12	45,1	0,0001536

HCH1	Ortholog of S. cerevisiae Hch1, a regulator of heat shock protein Hsp90	12	7	26,4	0,0001531
GIR2	Ortholog of S. cerevisiae Gir2	11	26	98,4	0,0001525
orf19.5131	Ortholog of S. cerevisiae Gid7, a GID complex protein	12	24	90,9	0,0001524
GCD6	Ortholog of S. cerevisiae Gcd6	8	18	68,2	0,0001523
ERO1	Ortholog of S. cerevisiae Ero1	11	35	133,8	0,000151
AXL2	Ortholog of S. cerevisiae Axl2	17	15	57,7	0,0001501
orf19.409	Ortholog of S. cerevisiae Aim38/Rcf2, cytochrome c oxidase subunit	16	22	84,9	0,0001496
ABP1	Ortholog of S. cerevisiae Abp1	16	8	30,9	0,0001494
orf19.1815	Ortholog of S. cerevisae/S. pombe Tif6	7	18	69,7	0,0001491
orf19.1578	Ortholog of S. cereviaie Rrp5, an RNA binding protein involved in synthesis of 18S and 5.8S rRNAs	11	15	58,2	0,0001488
orf19.6852	Ortholog of Rmd6 involved in S. cerevisiae sporulation	10	11	42,7	0,0001487
NDT80	Ortholog of Ndt80	12	8	31,1	0,0001485
URA3	Orotidine-5'-phosphate decarboxylase	9	17	66,2	0,0001482
CAR2	Ornithine aminotransferase	8	21	81,8	0,0001482
orf19.3060	OPutative dolichyl-diphosphooligosaccharide-protein glycotransferase	13	18	70,4	0,0001476
MET15	O-acetylhomoserine O-acetylserine sulfhydrylase	16	12	47,3	0,0001464
NAP1	Nucleosome assembly protein	16	21	82,8	0,0001464
YNK1	Nucleoside diphosphate kinase (NDP kinase)	9	7	27,7	0,0001459
NOP1	Nucleolar protein	11	23	92,1	0,0001442
orf19.7215	Nucleolar protein	15	19	76,1	0,0001441
MEX67	Nuclear export protein	6	13	52,1	0,000144
DFG5	N-linked mannoprotein of cell wall and membrane	7	12	48,1	0,000144
YHB1	Nitric oxide dioxygenase	8	7	28,2	0,0001433
NDH51	Nicotinamide adenine dinucleotide dehydrogenase complex I subunit of the mitochondrial electron transport chain	14	12	48,5	0,0001428
NTH1	Neutral trehalase	10	15	60,9	0,0001422
APE2	Neutral arginine, alanine, leucine specific metallo-aminopeptidase	9	17	69,3	0,0001416
EGD2	Nascent polypeptide associated complex protein alpha subunit	7	12	49,1	0,0001411
NCP1	NADPH-cytochrome P450 reductase, acts with Erg11p in sterol 14 alpha-demethylation in ergosterol biosynthesis	9	22	90,3	0,0001406
EBP1	NADPH oxidoreductase	6	12	49,4	0,0001402
GDH3	NADP-glutamate dehydrogenase	6	9	37,4	0,0001389
TDH3	NAD-linked glyceraldehyde-3-phosphate dehydrogenase	11	11	45,8	0,0001386
MCR1	NADH-cytochrome-b5 reductase	18	21	87,6	0,0001384
ALD5	NAD-aldehyde dehydrogenase	11	14	58,4	0,0001384
OYE32	NAD(P)H oxidoreductase family protein	12	19	79,5	0,000138
DAC1	N-acetylglucosamine-6-phosphate (GlcNAcP) deacetylase	11	15	63,3	0,0001368
NGT1	N-acetylglucosamine (GlcNAc)-specific transporter	14	25	105,8	0,0001364
HXK1	N-acetylglucosamine (GlcNAc) kinase	16	15	63,5	0,0001364
NMT1	Myristoyl-CoA:protein N-myristoyltransferase	11	11	46,6	0,0001363
HIS4	Multifunctional enzyme that catalyzes three steps of histidine biosynthesis, with phosphoribosyl-AMP cyclohydrolase, phosphoribosyl-ATP diphosphatase, and histidinol dehydrogenase activities	12	11	46,7	0,000136
CDR1	Multidrug transporter of ABC superfamily	10	11	46,8	0,0001357
FET34	Multicopper ferroxidase	10	14	59,9	0,0001349
MSB2	Mucin family adhesin-like protein	10	10	42,8	0,0001349
CAK1	Monomeric CDK-activating kinase	13	10	42,8	0,0001349
SHM1	Mitochondrial serine hydroxymethyltransferase	14	15	64,4	0,0001344
MRP7	Mitochondrial ribosomal protein of the large subunit	10	7	30,3	0,0001334
MRP8	Mitochondrial ribosomal protein	6	9	39	0,0001332
PTC5	Mitochondrial protein phosphatase of the Type 2C-related family (serine/threonine-specific), involved in drug response and cadmium tolerance	4	14	60,8	0,0001329
MMD1	Mitochondrial protein	7	23	99,9	0,0001329

POR1	Mitochondrial outer membrane porin	7	9	39,1	0,0001329
orf19.4947	Mitochondrial membrane protein of unknown function	7	9	39,2	0,0001325
MDH1	Mitochondrial malate dehydrogenase	7	29	126,8	0,000132
orf19.4396	Mitochondrial inner membrane protein	12	11	48,2	0,0001317
CRC1	Mitochondrial carnitine carrier protein	13	11	48,3	0,0001315
MIS12	Mitochondrial C1-tetrahydrofolate synthase precursor	5	12	53,2	0,0001302
ATP17	Mitochondrial ATPase complex subunit	12	19	84,3	0,0001301
PET9	Mitochondrial ADP/ATP carrier protein involved in ATP biosynthesis	13	32	142,4	0,0001297
MRPL8	Mitochondrial 60S ribosomal protein subunit	6	9	40,2	0,0001292
MVD	Mevalonate diphosphate decarboxylase	13	11	49,2	0,0001291
CRG1	Methyltransferase involved in sphingolipid homeostasis, methylates a drug cantharidin	15	11	49,2	0,0001291
GRP2	Methylglyoxal reductase	9	13	58,2	0,0001289
orf19.7264 (RP	Metalloprotease subunit of the 19S regulatory particle of the 26S proteasome lid	7	16	71,8	0,0001286
orf19.6553	Membrane-localized protein of unknown function	8	10	44,9	0,0001286
orf19.1546	Membrane-localized protein of unknown function	15	10	45	0,0001283
orf19.1682	Membrane protein	13	20	90,1	0,0001281
MRS7	Member of the LETM1-like protein family, mitochondrial membrane protein	10	26	117,3	0,0001279
DRG1	Member of the DRG family of GTP-binding proteins	11	6	27,1	0,0001278
RLI1	Member of RNase L inhibitor (RLI) subfamily of ABC family	10	14	63,3	0,0001277
VAN1	Member of Mnn9 family of mannosyltransferases	7	8	36,2	0,0001276
PBS2	MAPK kinase (MAPKK)	12	31	140,4	0,0001275
HOG1	MAP kinase of osmotic-, heavy metal-, and core stress response	8	10	45,3	0,0001274
MKC1	MAP kinase	6	12	54,6	0,0001269
KTR4	Mannosyltransferase	3	19	86,6	0,0001266
PSA2	Mannose-1-phosphate guanyltransferase	14	13	59,3	0,0001265
CCC1	Manganese transporter	9	14	64,1	0,0001261
orf19.3982	Maltase	9	22	100,9	0,0001259
MAE1	Malic enzyme, mitochondrial	12	10	45,9	0,0001258
MLS1	Malate synthase	9	8	36,8	0,0001255
HMT1	Major type I protein arginine methyltransferase (PRMT)	9	10	46,1	0,0001252
SAP2	Major secreted aspartyl proteinase	14	16	74,4	0,0001241
orf19.967	Major mitochondrial nuclease	9	19	88,4	0,0001241
CHS3	Major chitin synthase of yeast and hyphae	10	8	37,3	0,0001238
CAT2	Major carnitine acetyl transferase	10	10	46,7	0,0001236
RIB4	Lumazine synthase (6,7-dimethyl-8-ribityllumazine synthase, DMRL synthase)	19	12	56,1	0,0001235
orf19.2639.1	Lsm (Like Sm) protein	12	12	56,2	0,0001233
NUP82	Linker nucleoporin of the nuclear pore complex	10	11	51,6	0,0001231
VPS21	Late endosomal Rab small monomeric GTPase involved in transport of endocytosed proteins to the vacuole	6	6	28,2	0,0001228
POL3	Large subunit of DNA polymerase III	8	11	51,8	0,0001226
ERG11	Lanosterol 14-alpha-demethylase	6	8	37,8	0,0001222
ILV5	Ketol-acid reductoisomerase	17	18	85,3	0,0001218
KEL1	Kelch repeat domain-containing protein	11	20	94,9	0,0001217
NMD5	Karyopherin	17	24	113,9	0,0001216
LEU2	Isopropyl malate dehydrogenase	10	17	81,1	0,000121
ICL1	Isocitrate lyase	8	12	57,3	0,0001209
IDP2	Isocitrate dehydrogenase	14	8	38,2	0,0001209
INO1	Inositol-1-phosphate synthase	3	18	86,6	0,00012
IMH3	Inosine monophosphate (IMP) dehydrogenase	6	7	33,7	0,0001199
ECE1	Hypha-specific protein	13	13	63	0,0001191
CSP37	Hyphal cell wall protein	5	8	38,8	0,000119
SSB1	HSP70 family heat shock protein	13	12	58,2	0,000119
SSA2	HSP70 family chaperone	5	10	48,7	0,0001185
MET2	Homoserine acetyltransferase	14	18	87,9	0,0001182
orf19.6148	Homolog of nuclear distribution factor NudE, NUDEL	8	16	78,7	0,0001174
LYS12	Homoisocitrate dehydrogenase	12	54	265,7	0,0001173

LYS21	Homocitrate synthase	9	8	39,4	0,0001172
LYS4	Homoaconitase	6	12	59,2	0,000117
HMG1	HMG-CoA reductase	9	22	108,6	0,0001169
GCF1	HMG box mitochondrial protein	7	15	74,5	0,0001162
HDA1	Histone deacetylase	17	18	89,6	0,000116
PHO84	High-affinity phosphate transporter	6	11	54,9	0,0001157
HGT1	High-affinity MFS glucose transporter	11	17	85	0,0001154
FTR1	High-affinity iron permease	15	7	35	0,0001154
FTR2	High-affinity iron permease	4	25	125,6	0,0001149
PDE2	High affinity cyclic nucleotide phosphodiesterase	16	4	20,1	0,0001149
HXK2	Hexokinase II	7	27	135,8	0,0001148
orf19.6066	Hexadecenal dehydrogenase	5	17	85,8	0,0001144
LYS2	Heterodimeric alpha-aminoadipate reductase large subunit	8	18	90,9	0,0001143
HMX1	Heme oxygenase	14	12	60,6	0,0001143
HSP104	Heat-shock protein	12	13	65,7	0,0001142
HSP78	Heat-shock protein	9	12	60,9	0,0001137
HSP60	Heat shock protein	9	22	112,3	0,0001131
SSC1	Heat shock protein	10	31	158,3	0,000113
HAT1	Hat1-Hat2 histone acetyltransferase complex subunit	8	20	102,4	0,0001127
VMA4	H+ transporting ATPase E chain	8	19	97,7	0,0001123
SEC2	Guanyl-nucleotide exchange factor for the small G-protein Sec4	13	14	72,2	0,0001119
RGD1	GTPase activator protein	7	7	36,1	0,0001119
RAC1	G-protein of RAC subfamily	10	18	92,9	0,0001118
GPA2	G-protein alpha subunit	6	22	113,7	0,0001117
CHT2	GPI-linked chitinase	9	13	67,2	0,0001117
RBT5	GPI-linked cell wall protein	8	20	103,4	0,0001117
RHD3	GPI-anchored yeast-associated cell wall protein	8	7	36,2	0,0001116
ECM331	GPI-anchored protein	7	19	98,3	0,0001116
HYR1	GPI-anchored hyphal cell wall protein	10	6	31,1	0,0001114
CRH11	GPI-anchored cell wall transglycosylase, putative ortholog of S. cerevisiae Crh1p	5	14	72,6	0,0001113
EXG2	GPI-anchored cell wall protein, similar to S. cerevisiae exo-1,3-beta-glucosidase Exg2p	9	9	46,7	0,0001112
ECM33	GPI-anchored cell wall protein	10	15	78,4	0,0001104
PGA52	GPI-anchored cell surface protein of unknown function	7	15	78,5	0,0001103
PGA4	GPI-anchored cell surface protein	11	11	57,7	0,00011
PLB3	GPI-anchored cell surface phospholipase B	18	11	57,7	0,00011
GDA1	Golgi membrane GDPase, required for wild-type O-mannosylation, not N-glycosylation	8	9	47,3	0,0001098
PHR2	Glycosidase	7	6	31,6	0,0001096
GDE1	Glycerophosphocholine phosphodiesterase	11	17	89,6	0,0001095
GUT2	Glycerol-3-phosphate dehydrogenase	14	12	63,5	0,0001091
GPD1	Glycerol-3-phosphate dehydrogenase	9	21	111,8	0,0001084
RHR2	Glycerol 3-phosphatase	12	12	63,9	0,0001084
GLX3	Glutathione-independent glyoxalase	6	17	90,7	0,0001082
FDH3	Glutathione-dependent formaldehyde dehydrogenase	6	19	101,8	0,0001077
GTT11	Glutathione S-transferase, localized to ER	16	9	48,4	0,0001073
GST2	Glutathione S transferase	9	14	75,4	0,0001072
GLR1	Glutathione reductase	9	16	86,3	0,000107
PGI1	Glucose-6-phosphate isomerase	11	13	70,2	0,0001069
ZWF1	Glucose-6-phosphate dehydrogenase	9	11	59,6	0,0001065
GFA1	Glucosamine-6-phosphate synthase, homotetrameric enzyme of chitin/hexosamine biosynthesis	9	20	108,6	0,0001063
NAG1	Glucosamine-6-phosphate deaminase	13	14	76,1	0,0001062
GNA1	Glucosamine-6-phosphate acetyltransferase	6	10	54,4	0,0001061
orf19.7214	Glucan 1,3-beta-glucosidase	12	13	70,8	0,000106
orf19.4839	GET complex subunit	7	8	43,6	0,0001059
GCD11	Gamma subunit of translation initiation factor eIF2	7	19	104	0,0001055
GAL1	Galactokinase	10	8	43,8	0,0001054
UBI3	Fusion of ubiquitin with the S34 protein of the small ribosomal subunit	8	7	38,6	0,0001047

YRB1	Functional homolog of S. cerevisiae Yrb1p	8	8	44,2	0,0001045
YPT1	Functional homolog of S. cerevisiae Ypt1p, which is an essential small Ras-type GTPase involved in protein secretion at ER-to-Golgi	13	6	33,2	0,0001043
URE2	Functional homolog of S. cerevisiae Ure2p, which is a regulator of nitrogen utilization, and which also has an infectious prion form called [URE3]	5	10	55,5	0,000104
CSH3	Functional homolog of S. cerevisiae Shr3p, which is a chaperone specific for amino acid permeases	4	10	55,6	0,0001038
SEC18	Functional homolog of S. cerevisiae Sec18p, which acts in protein transport	7	9	50,1	0,0001037
SAR1	Functional homolog of S. cerevisiae Sar1	9	16	89,6	0,0001031
RPB7	Functional homolog of S. cerevisiae Rpb7	7	9	50,4	0,0001031
PHO85	Functional homolog of S. cerevisiae Pho85p, a cyclin-dependent kinase that regulates transcription of PHO genes involved in phosphate metabolism	14	16	89,9	0,0001027
JEM1	Functional homolog of S. cerevisiae Jem1p, which acts with Scj1p and Kar2p (BiP) in protein folding and ER-associated degradation of misfolded proteins, and also has a role in karyogamy	18	8	45	0,0001026
HAS1	Functional homolog of S. cerevisiae Has1p, which is a nucleolar protein of the DEAD-box ATP-dependent RNA helicase family that is involved in biogenesis of the ribosome, particularly the small (40S) subunit	9	10	56,4	0,0001023
GCR3	Functional homolog of S. cerevisiae Gcr3, which acts in regulation of glycolytic genes	11	12	67,8	0,0001022
FUN12	Functional homolog of S. cerevisiae Fun12 translation initiation factor eIF5B	4	10	56,5	0,0001022
SSN6	Functional homolog of S. cerevisiae Cyc8/Ssn6	11	15	84,8	0,0001021
CRM1	Functional homolog of S. cerevisiae Crm1, which acts in protein nuclear export	12	11	62,4	0,0001018
CDC68	Functional homolog of S. cerevisiae Cdc68, a transcription elongation factor	7	38	215,9	0,0001016
FUM11	Fumarate hydratase	12	23	131,1	0,0001013
FBA1	Fructose-bisphosphate aldolase	16	7	39,9	0,0001013
FBP1	Fructose-1,6-bisphosphatase	11	7	40	0,000101
FDH1	Formate dehydrogenase	9	9	51,5	0,0001009
RCT1	Fluconazole-induced protein	9	11	63,2	0,0001005
YHB5	Flavohemoglobin-related protein	9	7	40,4	0,0001
orf19.6552	Flavin-linked sulfhydryl oxidase	6	9	52	9,991E-05
orf19.3307	Flavin-containing monooxygenase	11	11	64,1	9,906E-05
SAC6	Fimbrin	7	9	52,5	9,895E-05
NUP159	FG-nucleoporin component of central core of the nuclear pore complex (NPC)	6	13	76,3	9,835E-05
CDC4	F-box subunit of SCF(CDC4) ubiquitin ligase	10	17	100,1	9,803E-05
OLE1	Fatty acid desaturase, essential protein involved in oleic acid synthesis	17	4	23,6	9,784E-05
ATP3	F1-ATP synthase complex subunit	10	15	88,7	9,762E-05
ATP2	F1 beta subunit of F1F0 ATPase complex	5	11	65,2	9,739E-05
GCA1	Extracellular/plasma membrane-associated glucoamylase	9	6	35,8	9,674E-05
XOG1	Exo-1,3-beta-glucanase	18	6	35,8	9,674E-05
HBR1	Essential protein involved in regulation of MTL gene expression	7	8	48	9,621E-05
SEC14	Essential protein	8	7	42,1	9,598E-05
SNF1	Essential protein	9	20	120,8	9,557E-05
UTP8	Essential nucleolar protein	7	5	30,2	9,557E-05
MSI3	Essential HSP70 family protein	12	16	97,3	9,492E-05
SRB1	Essential GDP-mannose pyrophosphorylase	6	13	79,4	9,451E-05
HSP90	Essential chaperone, regulates several signal transduction pathways and temperature-induced morphogenesis	8	26	159,9	9,386E-05
KRE6	Essential beta-1,6-glucan synthase subunit	8	18	113,1	9,187E-05
GSC1	Essential beta-1,3-glucan synthase subunit	6	13	81,9	9,162E-05

MET6	Essential 5-methyltetrahydropteroyltriglutamate-homocysteine methyltransferase (cobalamin-independent methionine synthase)	7	13	82,1	9,14E-05
HSE1	ESCRT-0 complex subunit	10	7	44,5	9,08E-05
CEK1	ERK-family protein kinase	10	14	89,1	9,07E-05
SEC61	ER protein-translocation complex subunit	3	17	109	9,003E-05
SEC72	ER protein-translocation complex component	7	8	51,4	8,984E-05
orf19.5689	Epsilon-COP subunit of the coatomer	10	15	97,1	8,917E-05
ENO1	Enolase	8	11	71,3	8,905E-05
PRB1	Endoprotease B	10	6	38,9	8,903E-05
orf19.491	Endoplasmic reticulum (ER) protein-translocation complex subunit	7	10	64,9	8,894E-05
ENG1	Endo-1,3-beta-glucanase	8	6	39	8,881E-05
EFT2	Elongation Factor 2 (eEF2)	6	12	78,3	8,847E-05
LSP1	Eisosome component with a predicted role in endocytosis	12	9	59,3	8,761E-05
PIL1	Eisosome component	5	9	59,4	8,746E-05
VPS1	Dynamin-family GTPase-related protein	8	6	39,9	8,68E-05
XYL2	D-xylulose reductase	6	8	53,4	8,648E-05
DUT1	dUTP pyrophosphatase	8	12	80,3	8,626E-05
orf19.3994	Dolichyl-diphosphooligosaccharide-protein glycotransferase	6	7	47,1	8,579E-05
ILV3	Dihydroxyacid dehydratase	5	24	162	8,552E-05
URA1	Dihydroorotate dehydrogenase	8	8	54,2	8,52E-05
URA4	Dihydroorotase	6	11	74,6	8,512E-05
orf19.3475	Described as a Gag-related protein	8	8	54,4	8,489E-05
orf19.1626	Deoxyhypusine synthase	9	14	95,5	8,462E-05
FAD2	Delta-12 fatty acid desaturase, involved in production of linoleic acid, which is a major component of membranes	11	14	96,3	8,392E-05
ERG6	Delta(24)-sterol C-methyltransferase, converts zymosterol to fecosterol, ergosterol biosynthesis	7	17	117,1	8,38E-05
RET2	Delta subunit of the coatomer complex (COPI)	12	11	75,8	8,377E-05
PRO3	Delta 1-pyrroline-5-carboxylate reductase	10	5	34,9	8,27E-05
ARD	D-arabitol dehydrogenase, NAD-dependent (ArDH)	7	11	77,8	8,161E-05
ARA1	D-Arabinose dehydrogenase	5	10	70,9	8,142E-05
ALO1	D-Arabinono-1,4-lactone oxidase involved in biosynthesis of dehydro-D-arabinono-1,4-lactone, which has a protective role against oxidative damage	8	4	28,5	8,102E-05
CDC60	Cytosolic leucyl tRNA synthetase	7	19	136,1	8,058E-05
orf19.2720	Cytosolic chaperonin Cct ring complex subunit	6	12	86	8,054E-05
CCT7	Cytosolic chaperonin Cct ring complex	8	16	114,9	8,038E-05
SHM2	Cytoplasmic serine hydroxymethyltransferase	9	29	208,9	8,013E-05
MES1	Cytoplasmic methionyl-tRNA synthetase	5	10	72,3	7,984E-05
ECM4	Cytoplasmic glutathione S-transferase	6	5	36,2	7,973E-05
CCP1	Cytochrome-c peroxidase N terminus	5	16	117,7	7,847E-05
COX5	Cytochrome oxidase subunit V	6	6	44,3	7,818E-05
CYT1	Cytochrome c1	5	6	44,4	7,8E-05
CYC1	Cytochrome c	10	16	118,6	7,787E-05
CYB5	Cytochrome b(5)	6	11	81,7	7,772E-05
CYS3	Cystathionine gamma-lyase	7	11	81,8	7,762E-05
CYS4	Cystathionine beta-synthase	5	12	89,8	7,714E-05
CDC28	Cyclin-dependent protein kinase	7	16	119,9	7,703E-05
CDC53	Cullin, a scaffold subunit of the SCF ubiquitin-ligase complexes	5	13	97,5	7,696E-05
SOD5	Cu and Zn-containing superoxide dismutase	4	9	67,8	7,662E-05
URA7	CTP synthase 1	6	12	90,5	7,654E-05
CBP1	Corticosteroid binding protein	7	5	37,8	7,635E-05
CRN1	Coronin	8	7	53	7,624E-05
HEM13	Coproporphyrinogen III oxidase	11	13	98,9	7,588E-05
CTR1	Copper transporter	7	13	99,2	7,565E-05
CCS1	Copper chaperone involved in activation and protection of superoxide dismutase Sod1p	7	5	38,6	7,477E-05
EMP24	COPII-coated vesicle component	4	16	123,7	7,466E-05
RPP2B	Conserved acidic ribosomal protein	14	6	46,4	7,464E-05

SAM51	Component of the SAM complex involved in mitochondrial protein import, involved in beta-barrel protein assembly	8	6	46,5	7,448E-05
RPL32	Component of the large (60S) ribosomal subunit	5	9	69,8	7,443E-05
ARP2	Component of the Arp2/3 complex	7	7	54,4	7,428E-05
ERV25	Component of COPII-coated vesicles	7	13	101,2	7,415E-05
TSR1	Component of 20S pre-rRNA processing unit	6	16	125,5	7,359E-05
PGA63	Component COPII vesicle coat	11	8	63,1	7,318E-05
CHC1	Clathrin heavy chain	4	10	79,1	7,298E-05
MYO2	Class V myosin	8	10	79,9	7,224E-05
BRO1	Class E vacuolar protein sorting factor	4	11	88,2	7,199E-05
CIT1	Citrate synthase	4	6	48,5	7,141E-05
CHT1	Chitinase	11	6	48,8	7,097E-05
TCP1	Chaperonin-containing T-complex subunit, induced by alpha pheromone in SpiderM medium	8	12	97,8	7,083E-05
CCT8	Chaperonin-containing T-complex subunit	5	10	82,2	7,022E-05
CCT2	Chaperonin of the cytosolic TCP1 ring complex	7	8	66,2	6,976E-05
CDC37	Chaperone for Crk1p	6	7	58,2	6,943E-05
orf19.6189	Chalcone related protein family	8	10	83,4	6,921E-05
ALS1	Cell-surface adhesin	8	14	118,1	6,843E-05
TMA19	Cell wall protein, ortholog of S. cerevisiae Tma19p (Ykl065cp)	8	6	50,7	6,831E-05
RBT1	Cell wall protein with similarity to Hwp1	8	4	33,9	6,811E-05
SCW11	Cell wall protein	7	6	50,9	6,804E-05
SUN41	Cell wall glycosidase	6	4	34	6,791E-05
SMI1	Cell wall biosynthesis protein	4	12	102,4	6,764E-05
ALS3	Cell wall adhesin	6	24	208,6	6,641E-05
BGL2	Cell wall 1,3-beta-glucosyltransferase	5	9	79,1	6,568E-05
PRA1	Cell surface protein that sequesters zinc from host tissue	6	13	114,6	6,548E-05
MP65	Cell surface mannoprotein	4	7	62,4	6,475E-05
PHR1	Cell surface glycosidase	5	7	62,5	6,465E-05
CMP1	Catalytic subunit of calcineurin (Ca[2+]-calmodulin-regulated S/T protein phosphatase)	9	7	63,3	6,383E-05
CKA2	Catalytic alpha-subunit of protein kinase CK2	3	15	136,9	6,325E-05
CAT1	Catalase	7	10	91,6	6,302E-05
orf19.7404	Catabolic L-serine (L-threonine) deaminase	3	11	100,8	6,299E-05
CTN1	Carnitine acetyl transferase	6	8	74,1	6,232E-05
CPY1	Carboxypeptidase Y	4	11	102,2	6,213E-05
NCE103	Carbonic anhydrase	15	7	65,8	6,141E-05
orf19.2737	Carbohydrate kinase domain-containing protein	4	5	47	6,141E-05
TPK2	cAMP-dependent protein kinase catalytic subunit	8	8	75,6	6,108E-05
TPK1	cAMP-dependent protein kinase catalytic subunit	10	15	141,9	6,102E-05
orf19.5300	Calnexin	2	11	104,2	6,094E-05
ERG3	C-5 sterol desaturase	8	8	76	6,076E-05
ERG26	C-3 sterol dehydrogenase, catalyzes the 2nd of 3 steps required to remove 2 C-4 methyl groups from an intermediate in ergosterol biosynthesis	5	14	133,6	6,049E-05
BDF1	Bromodomain transcription factor	6	8	76,5	6,036E-05
orf19.7645	Biotin protein ligase	4	6	57,9	5,982E-05
TUB2	Beta-tubulin	9	5	48,3	5,976E-05
HEX1	Beta-N-acetylhexosaminidase/chitobiase, highly glycosylated enzyme that is secreted to the periplasm and culture medium	6	12	116	5,971E-05
BMT1	Beta-mannosyltransferase, required for addition of the 1st beta-mannose residue to acid-stable fraction of cell wall phosphopeptidomannan	6	11	106,4	5,968E-05
BMT3	Beta-mannosyltransferase	7	11	108,2	5,868E-05
BMT6	Beta-mannosyltransferase	6	8	78,8	5,86E-05
SSR1	Beta-glucan associated ser/thr rich cell-wall protein with a role in cell wall structure	3	9	88,7	5,857E-05
CDC43	Beta subunit of heterodimeric protein geranylgeranyltransferase type I	4	10	99,3	5,813E-05
FAS1	Beta subunit of fatty-acid synthase	3	6	60,7	5,706E-05

GVP36	BAR domain protein	8	5	51,2	5,637E-05
PIM1	ATP-dependent Lon protease	3	6	61,5	5,632E-05
ATP1	ATP synthase alpha subunit	4	7	72,1	5,604E-05
MET3	ATP sulfurlyase	6	8	82,4	5,604E-05
HIS1	ATP phosphoribosyl transferase	9	18	186,2	5,58E-05
AAT1	Aspartate aminotransferase	3	8	82,8	5,577E-05
AAT22	Aspartate aminotransferase	7	6	62,3	5,559E-05
ARO8	Aromatic transaminase of the Ehrlich fusel oil pathway of aromatic alcohol biosynthesis	8	9	95,5	5,44E-05
ARO9	Aromatic transaminase	4	12	127,9	5,416E-05
ARG1	Argininosuccinate synthase	6	9	97,3	5,339E-05
ARG4	Argininosuccinate lyase, catalyzes the final step in the arginine biosynthesis pathway	3	12	130,3	5,316E-05
ARG5,6	Arginine biosynthetic enzyme	6	8	87,1	5,302E-05
TUB1	Alpha-tubulin	7	8	87,3	5,29E-05
ROT2	Alpha-glucosidase II, catalytic subunit, required for N-linked protein glycosylation and normal cell wall synthesis	8	6	65,5	5,288E-05
MAL2	Alpha-glucosidase	7	6	66	5,248E-05
PRE5	Alpha6 subunit of the 20S proteasome	6	4	44,5	5,189E-05
PUP2	Alpha5 subunit of the 20S proteasome	4	8	90,3	5,114E-05
PRE9	Alpha3 (C9) subunit of the 20S proteasome	5	7	80,5	5,019E-05
OCH1	Alpha-1,6-mannosyltransferase	5	14	161,7	4,998E-05
MNN2	Alpha-1,2-mannosyltransferase	5	12	139,4	4,969E-05
MNN24	Alpha-1,2-mannosyltransferase	3	6	69,7	4,969E-05
MNN23	Alpha-1,2-mannosyltransferase	4	7	84,3	4,793E-05
ALG11	Alpha-1,2-mannosyltransferase	3	18	217,9	4,768E-05
MNT1	Alpha-1,2-mannosyl transferase	2	13	157,4	4,768E-05
MNT2	Alpha-1,2-mannosyl transferase	6	17	207,6	4,727E-05
MNS1	Alpha-1,2-mannosidase	5	6	73,5	4,712E-05
orf19.100	Alpha/beta hydrolase and lipase domain protein	7	4	49,4	4,674E-05
OST1	Alpha subunit of the oligosaccharyltransferase complex of the ER lumen	4	8	98,9	4,669E-05
FAS2	Alpha subunit of fatty-acid synthase	6	12	149,8	4,624E-05
orf19.1672	Alpha subunit of COPI vesicle coatomer complex	6	5	62,6	4,611E-05
AHP1	Alkyl hydroperoxide reductase	7	7	87,9	4,597E-05
SLK19	Alkaline-induced protein of plasma membrane	3	6	75,9	4,563E-05
CSH1	Aldo-keto reductase	4	9	116,3	4,467E-05
orf19.7306	Aldo-keto reductase	5	5	65,2	4,427E-05
IFD6	Aldo-keto reductase	3	10	130,8	4,413E-05
ADH1	Alcohol dehydrogenase	7	5	66,3	4,353E-05
ADH2	Alcohol dehydrogenase	4	8	107,1	4,312E-05
ALA1	Alanyl-tRNA synthetase	3	7	94	4,299E-05
OP4	Ala- Leu- and Ser-rich protein	4	3	40,5	4,276E-05
ARF1	ADP-ribosylation factor	4	10	135,1	4,273E-05
SIM1	Adhesin-like protein	8	5	68,2	4,232E-05
ASR3	Adenylyl cyclase and stress responsive protein	6	11	150,3	4,225E-05
ADE12	Adenylosuccinate synthase	2	7	99,4	4,065E-05
ADE13	Adenylosuccinate lyase	13	4	57,4	4,023E-05
SRV2	Adenylate cyclase-associated protein	4	14	204,2	3,958E-05
ADO1	Adenosine kinase	3	8	117,9	3,917E-05
APT1	Adenine phosphoribosyltransferase	5	5	73,8	3,911E-05
LRO1	Acyltransferase that catalyzes diacylglycerol esterification of phospholipids	4	6	89,6	3,865E-05
CHS4	Activator of Chs3p chitin synthase	3	7	105,6	3,826E-05
SLA2	Actin binding protein with roles in growth control and morphogenesis	5	10	152	3,798E-05
ACT1	Actin	4	9	138,8	3,743E-05
ACO1	Aconitase	3	24	375,3	3,691E-05
RPS12	Acidic ribosomal protein S12	3	8	127	3,636E-05
RPP2A	Acidic ribosomal protein	4	11	180,6	3,516E-05
PHO114	Acid phosphatase	5	5	82,2	3,511E-05

ACS2	Acetyl-CoA synthetase	4	4	66,5	3,472E-05
ACS1	Acetyl-CoA synthetase	5	7	118,8	3,401E-05
ACH1	Acetyl-coA hydrolase	4	6	104,5	3,314E-05
ERG10	Acetyl-CoA acetyltransferase	3	13	228,4	3,285E-05
VPS4	AAA-ATPase involved in transport from MVB to the vacuole and ESCRT-III complex disassembly	5	6	107,6	3,219E-05
GND1	6-phosphogluconate dehydrogenase	5	6	109,3	3,169E-05
RPL38	60S ribosomal ribosomal protein subunit	3	6	110,1	3,146E-05
orf19.2478.1	60S ribosomal protein L7	3	5	92,3	3,127E-05
ADE6	5-Phosphoribosylformyl glycinamidine synthetase	3	5	94,4	3,057E-05
ADE17	5-Aminoimidazole-4-carboxamide ribotide transformylase, enzyme of adenine biosynthesis	4	5	97,3	2,966E-05
PHO15	4-nitrophenyl phosphatase, possible histone H2A phosphatase	4	8	156,3	2,955E-05
ASC1	40S ribosomal subunit similar to G-beta subunits	5	5	98,3	2,936E-05
ERG27	3-Keto sterol reductase of ergosterol biosynthesis	2	5	104	2,775E-05
LEU1	3-isopropylmalate dehydratase	3	3	64	2,706E-05
FOX2	3-hydroxyacyl-CoA epimerase	2	9	193,2	2,689E-05
ERG13	3-hydroxy-3-methylglutaryl coenzyme A synthase	4	6	135,8	2,55E-05
ARO4	3-deoxy-D-arabinoheptulosonate-7-phosphate synthase	4	3	69,7	2,485E-05
ARO3	3-deoxy-D-arabinoheptulosonate-7-phosphate synthase	2	6	144,2	2,402E-05
RPT5	26S proteasome regulatory subunit	2	6	164,3	2,108E-05
RPT4	26S proteasome regulatory subunit	3	7	193,1	2,093E-05
orf19.4230 (PR	20S proteasome subunit (beta7)	3	4	111,1	2,078E-05

Table S2 a. Proteins identified in at least 2 replicates of YWCL (yeast whole cell extract) with at least 2 peptides in a replicate

	Description	# AAs	MW [kDa]	Unique Peptides
AAT1	Aspartate aminotransferase	437	48,9	24
AAT21	Putative aspartate aminotransferase	416	46	10
AAT22	Aspartate aminotransferase	409	46,8	3
ABP1	Ortholog of S. cerevisiae Abp1	648	70,6	32
ACB1	Protein similar to a region of acyl-coenzyme-A-binding protein	86	9,9	5
ACC1	Putative acetyl-coenzyme-A carboxylases	2271	253,3	48
ACH1	Acetyl-coA hydrolase	524	57,9	24
ACO1	Aconitase	777	84,2	30
ACO2	Putative aconitate hydratase 2	791	85,9	8
ACS1	Acetyl-CoA synthetase	675	75,1	13
ACS2	Acetyl-CoA synthetase	676	74,3	27
ACT1	Actin	376	41,7	10
ADE1	Phosphoribosylaminoimadazole succinocarboxamide synthetase, enzyme of adenine biosynthesis	291	32,9	10
ADE12	Adenylosuccinate synthase	428	47,9	9
ADE13	Adenylosuccinate lyase	482	54,4	17
ADE17	5-Aminoimidazole-4-carboxamide ribotide transformylase, enzyme of adenine biosynthesis	592	64,9	23
ADE2	Phosphoribosylaminoimadazole carboxylase	568	62,4	7
ADE5,7	Phosphoribosylamine-glycine ligase and phosphoribosylformylglycinamidine cyclo-ligase	802	86	16
ADE6	5-Phosphoribosylformyl glycinamidine synthetase	1370	150,7	15
ADH1	Alcohol dehydrogenase	349	36,8	19
ADH2	Alcohol dehydrogenase	348	36,8	13
ADH5	Putative alcohol dehydrogenase	336	35,7	6
ADK1	Putative adenylate kinase	249	27,6	14
ADO1	Adenosine kinase	347	38,2	11
AFG3	Similar to S. cerevisiae Afg3p, a subunit of the mitochondrial inner membrane m-AAA protease	795	88,7	4
AGC1	Putative mitochondrial carrier protein	731	81,9	5
AGM1	Phosphoacetylglucosamine mutase (N-acetylglucosamine-phosphate mutase)	544	60,4	10
AHA1	Putative Hsp90p co-chaperone	346	38,7	10
AHP1	Alkyl hydroperoxide reductase	176	19,2	5
ALA1	Alanyl-tRNA synthetase	969	108,2	26
ALD5	NAD-aldehyde dehydrogenase	499	53,9	29
ALD6	Putative aldehyde dehydrogenase	541	58,3	9
ALO1	D-Arabinono-1,4-lactone oxidase involved in biosynthesis of dehydro-D-arabinono-1,4-lactone, which has a protective role against oxidative damage	557	63,4	2
ALT1	Putative alanine transaminase	520	58,4	4
AMO2	Protein similar to A. niger predicted peroxisomal copper amino oxidase	671	76	10
AMS1	Putative alpha-mannosidase	1155	131,5	34
ANB1	Translation initiation factor eIF-5A	158	17,1	9
APE2	Neutral arginine, alanine, leucine specific metallo-aminopeptidase	924	104,3	31
APE3	Putative vacuolar aminopeptidase Y,	550	60,8	6
APR1	Vacuolar aspartic proteinase	419	45,4	6
APT1	Adenine phosphoribosyltransferase	188	20,9	5
ARA1	D-Arabinose dehydrogenase	326	37,4	9
ARC1	Putative G4 nucleic acid binding protein	369	40,9	14
ARC15	Putative ARP2/3 complex subunit	158	17,7	5

ARC19	Putative ARP2/3 complex subunit	173	20,1	4
ARC40	Protein similar to S. cerevisiae Arc40	384	42,3	4
ARD	D-arabitol dehydrogenase, NAD-dependent (ArDH)	281	30,7	6
ARF2	Putative ADP-ribosylation factor	181	20,6	9
ARG1	Argininosuccinate synthase	416	46,1	10
ARG3	Putative ornithine carbamoyltransferase	345	38,6	5
ARG4	Argininosuccinate lyase, catalyzes the final step in the arginine biosynthesis pathway	468	52,8	7
ARG5,6	Arginine biosynthetic enzyme	857	95,1	13
ARG8	Putative acetylornithine aminotransferase	455	50,1	4
ARO1	Putative pentafunctional arom enzyme	1551	169,4	15
ARO2	Putative chorismate synthase	413	44,8	6
ARO3	3-deoxy-D-arabinoheptulosonate-7-phosphate synthase	371	40,6	8
ARO4	3-deoxy-D-arabinoheptulosonate-7-phosphate synthase	370	40,3	14
ARO8	Aromatic transaminase of the Ehrlich fusel oil pathway of aromatic alcohol biosynthesis	491	54,7	9
ARP3	Protein with Myo5p-dependent localization to cortical actin patches at hyphal tip	419	46,6	6
ARP9	Protein similar to S. cerevisiae Arp3p, a component of the Arp2/3 complex involved in actin-dependent processes	484	54,6	4
ARX1	Putative ribosomal large subunit biogenesis protein	564	62,2	6
ASC1	40S ribosomal subunit similar to G-beta subunits	317	34,5	13
ASG1	Gal4p family zinc-finger transcription factor with similarity to S. cerevisiae Asg1p	990	111,4	2
ASN1	Putative asparagine synthetase	573	64,6	13
ASR1	Heat shock protein	385	37,2	7
ASR2	Adenylyl cyclase and stress responsive protein	258	27,8	9
ASR3	Adenylyl cyclase and stress responsive protein	114	13	3
ATP1	ATP synthase alpha subunit	546	58,9	26
ATP14	Putative mitochondrial F1F0 ATP synthase subunit	108	12	2
ATP16	Subunit of the mitochondrial F1F0 ATP synthase	161	17,6	3
ATP18	F1F0 ATP synthase complex subunit	64	7,3	2
ATP2	F1 beta subunit of F1F0 ATPase complex	504	53,9	22
ATP20	Putative mitochondrial ATP synthase	114	12,6	4
ATP3	F1-ATP synthase complex subunit	294	32	8
ATP4	Putative F0-ATP synthase subunit 4	233	25,8	12
ATP5	Putative F0-ATP synthase FO subunit B	205	22,1	9
ATP7	Putative subunit of the F1F0-ATPase complex	174	19,4	5
AYR1	Putative oxidoreductase	293	32,4	2
BAT21	Putative branched chain amino acid aminotransferase	393	43,8	5
BAT22	Putative branched chain amino acid aminotransferase	369	40,8	12
BBC1	Putative SH3-domain-containing protein	954	104,5	3
BCY1	Protein kinase A regulatory subunit	458	50,2	13
BFR1	Protein involved in the maintenance of normal ploidy	462	53,5	17
BLP1	Protein of unknown function, serum-induced	77	9	6
BMH1	Sole 14-3-3 protein in C. albicans	264	29,5	19
BRE1	Putative transcription factor with C3HC4 zinc finger DNA-binding motif	681	78,5	5
BRO1	Class E vacuolar protein sorting factor	945	108,6	2
BUB3	Protein similar to S. cerevisiae Bub3	373	42,8	3
BUD7	Protein of unknown function	764	86,2	9
CAM1	Putative translation elongation factor eEF1 gamma	412	47	12
CAM1-1	Putative translation elongation factor	419	47,5	9
CAP1	AP-1 bZIP transcription factor	499	54,9	4
CAR2	Ornithine aminotransferase	436	47,3	12

CAT1	Catalase	485	54,8	9
CAT2	Major carnitine acetyl transferase	632	71,4	21
CBF1	Transcription factor	251	29,1	3
CCS1	Copper chaperone involved in activation and protection of superoxide dismutase Sod1p	248	26,5	3
CCT2	Chaperonin of the cytosolic TCP1 ring complex	526	56,8	7
CCT3	Putative cytosolic chaperonin Cct ring complex subunit	529	58,6	10
CCT5	T-complex protein 1, epsilon subunit	548	59,8	8
CCT6	Putative cytosolic chaperonin Cct ring complex subunit	559	60,7	5
CCT7	Cytosolic chaperonin Cct ring complex	549	60,4	4
CCT8	Chaperonin-containing T-complex subunit	540	58,2	8
CDC10	Septin, required for wild-type cell, hyphal, or chlamydospore morphology	357	40,6	10
CDC11	Septin	402	46,7	5
CDC12	Septin	397	45,8	5
CDC19	Pyruvate kinase at yeast cell surface	504	55,4	30
CDC3	Septin	428	49	9
CDC37	Chaperone for Crk1p	508	58,5	4
CDC39	Protein similar to S. cerevisiae Cdc39p, which is part of the CCR4-NOT transcription regulatory complex	2016	228,4	4
CDC48	Putative microsomal ATPase	826	90,9	29
CDC60	Cytosolic leucyl tRNA synthetase	1097	125,3	27
CEF3	Translation elongation factor 3	1050	116,9	30
CGR1	Negative regulator of yeast-form growth	340	38,4	4
CHC1	Clathrin heavy chain	1659	188,2	29
CHS5	Putative chitin biosynthesis protein	562	62,1	5
CIP1	Possible oxidoreductase	299	33	8
CIT1	Citrate synthase	467	52	26
CLC1	Clathrin light chain	225	26	9
CMD1	Calmodulin	149	16,5	8
CMK2	Putative calmodulin-dependent protein kinase	477	53,7	8
CMP1	Catalytic subunit of calcineurin (Ca[2+]-calmodulin-regulated S/T protein phosphatase)	609	69,6	6
COF1	Putative cofilin	141	15,8	7
COQ5	Putative methyltransferase of ubiquinone biosynthesis	306	34,2	3
COX13	Cytochrome c oxidase	134	15,9	5
COX15	Cytochrome oxidase assembly protein	479	53,4	2
COX2	Subunit II of cytochrome c oxidase, which is the terminal member of the mitochondrial inner membrane electron transport chain	262	29,8	2
COX5	Cytochrome oxidase subunit V	165	18,6	7
COX6	Putative cytochrome c oxidase	150	17,4	4
COX9	Putative subunit VIIa of cytochrome c oxidase	61	6,9	2
CPA1	Putative carbamoyl-phosphate synthase subunit	430	46,9	4
CPA2	Putative arginine-specific carbamoylphosphate synthetase	1149	127	11
CPR3	Putative peptidyl-prolyl cis-trans isomerase	191	21	5
CPR6	Putative peptidyl-prolyl cis-trans isomerase	369	40,7	9
CPY1	Carboxypeptidase Y	542	61	3
CRC1	Mitochondrial carnitine carrier protein	284	30,1	5
CRG1	Methyltransferase involved in sphingolipid homeostasis, methylates a drug cantharidin	303	33,8	4
CRM1	Functional homolog of S. cerevisiae Crm1, which acts in protein nuclear export	1079	124	17
CRN1	Coronin	633	70,6	11
CSH1	Aldo-keto reductase	337	38,2	10
CSP37	Hyphal cell wall protein	321	37	15
CTA3	Protein similar to <i>S. cerevisiae</i> Ede1p, which is involved in endocytosis	1296	140,4	16

CTM1	Putative cytochrome c lysine methyltransferase	686	79	4
CTN1	Carnitine acetyl transferase	795	90,3	17
CTN3	Peroxisomal carnitine acetyl transferase	892	101	8
CUE5	Predicted ubiquitin-binding protein	426	48	7
CYB2	Putative cytochrome b2 precursor	560	62,9	2
CYC1	Cytochrome c	110	12,2	7
CYM1	Putative metalloprotease of the mitochondrial intermembrane space	1034	117,5	3
CYP1	Peptidyl-prolyl cis-trans isomerase	162	17,6	11
CYP5	Putative peptidyl-prolyl cis-trans isomerase	203	22,4	7
CYS4	Cystathionine beta-synthase	496	53,9	22
CYT1	Cytochrome c1	288	32,2	3
DAK2	Putative dihydroxyacetone kinase	595	64,9	21
DAP2	Putative dipeptidyl aminopeptidase	838	96,2	7
DBP2	Putative DEAD-box family ATP-dependent RNA helicase	562	61,2	2
DBP5	Protein of unknown function	540	60,1	6
DDI1	Putative DNA damage inducible v-SNARE binding protein	323	35,9	8
DDR48	Immunogenic stress-associated protein	212	22,6	20
DED1	Predicted ATP-dependent RNA helicase	672	72,8	2
DED81	Putative tRNA-Asn synthetase	552	62,2	17
DFG5	N-linked mannoprotein of cell wall and membrane	451	50	2
DHH1	Putative RNA helicase	549	62,1	3
DLD1	Putative D-lactate dehydrogenase	584	64,4	2
DNM1	Putative dynamin-related GTPase	866	97,8	3
DOA1	WD repeat protein	761	84,3	9
DOT5	Putative nuclear thiol peroxidase	263	29,1	15
DPS1-1	Putative tRNA-Asp synthetase	578	65,3	16
DQD1	Putative 3-dehydroquinate dehydratase	146	16	3
DTD2	Protein of unknown function	163	17,9	3
DUR1,2	Urea amidolyase	1813	200,3	23
DUT1	dUTP pyrophosphatase	159	16,9	4
ECM17	Putative sulfite reductase beta subunit	1437	160	9
ECM29	Putative scaffold protein	1859	208,6	6
ECM33	GPI-anchored cell wall protein	423	43,5	10
ECM4	Cytoplasmic glutathione S-transferase	334	39	15
EFB1	Translation elongation factor EF-1 beta	213	23,5	8
EFG1	bHLH transcription factor	525	57,1	2
EFT2	Elongation Factor 2 (eEF2)	842	93,3	39
EGD1	Putative GAL4 DNA-binding enhancer protein	157	17	7
EGD2	Nascent polypeptide associated complex protein alpha subunit	178	19,5	9
EHD3	Predicted 3-hydroxyisobutyryl-CoA hydrolase	502	56,2	3
EIF4E	Translation initiation factor eIF4E	209	24,2	11
ELF1	Putative mRNA export protein	1195	133,8	2
ENO1	Enolase	440	47,2	35
ENT3	Putative epsin	453	48,5	4
ERF1	Putative translation release factor 1, which interacts with stop codons and promotes release of nascent peptides from ribosomes	436	49,1	4
ERG10	Acetyl-CoA acetyltransferase	402	41,9	22
ERG11	Lanosterol 14-alpha-demethylase	528	60,6	2
ERG13	3-hydroxy-3-methylglutaryl coenzyme A synthase	451	49,7	14
ERG20	Putative farnesyl pyrophosphate synthetase involved in isoprenoid and sterol biosynthesis, based on similarity to S. cerevisiae Erg20p	351	40,7	10

ERO1	Ortholog of S. cerevisiae Ero1	580	66,9	3
ETR1	Putative 2-enoyl thioester reductase	364	39,9	7
FAA21	Predicted acyl CoA synthetase	718	80,3	2
FAA4	Predicted acyl CoA synthase	696	77,3	9
FAS1	Beta subunit of fatty-acid synthase	2037	227,7	67
FAS2	Alpha subunit of fatty-acid synthase	1884	207,2	66
FBA1	Fructose-bisphosphate aldolase	359	39,2	20
FBP1	Fructose-1,6-bisphosphatase	331	35,9	7
FDH1	Formate dehydrogenase	379	41,8	14
FDH3	Glutathione-dependent formaldehyde dehydrogenase	381	40,6	9
FESUR1	Putative ubiquinone reductase	223	25,1	3
FGR44	Protein lacking an ortholog in S. cerevisiae	410	46,8	4
FKH2	Forkhead transcription factor	687	76,3	2
FMA1	Putative oxidoreductase	253	27,4	6
FMP45	Predicted membrane protein induced during mating	285	31,2	4
FOX2	3-hydroxyacyl-CoA epimerase	906	99,3	23
FRS1	Phenylalanyl-tRNA synthetase	592	66,8	14
FRS2	Putative tRNA-Phe synthetase	496	56,4	15
FUM11	Fumarate hydratase	462	50	10
FUM12	Putative fumarate hydratase	510	55,4	14
FUN12	Functional homolog of S. cerevisiae Fun12 translation initiation factor eIF5B	1017	113	4
FUR1	Uracil phosphoribosyltransferase	218	24,5	4
GAD1	Putative glutamate decarboxylase	568	63,9	17
GAL1	Galactokinase	515	57,1	13
GAL10	UDP-glucose 4-epimerase	675	75,4	26
GAL7	Putative galactose-1-phoshphate uridyl transferase	386	44,7	12
GBP2	Putative single-strand telomeric DNA-binding protein	436	49,2	8
GCD11	Gamma subunit of translation initiation factor eIF2	534	58,5	6
GCF1	HMG box mitochondrial protein	245	28,5	3
GCN1	Protein of unknown function	2415	265,7	2
GCR3	Functional homolog of S. cerevisiae Gcr3, which acts in regulation of glycolytic genes	886	103,1	3
GCV1	Putative T subunit of glycine decarboxylase	394	43,7	5
GCV2	Glycine decarboxylase P subunit	999	109,8	8
GCV3	Glycine decarboxylase, subunit H	177	20	7
GCY1	Aldo/keto reductase	295	33	9
GDB1	Putative glycogen debranching enzyme	1527	173,8	14
GDH2	Putative NAD-specific glutamate dehydrogenase	1056	119,9	18
GDH3	NADP-glutamate dehydrogenase	456	49,5	26
GDI1	Putative Rab GDP-dissociation inhibitor	449	50,1	8
GFA1	Glucosamine-6-phosphate synthase, homotetrameric enzyme of chitin/hexosamine biosynthesis	713	79,2	5
GGA2	Protein involved in Golgi trafficking	584	64,7	4
GLC3	Putative 1,4-glucan branching enzyme	684	78,6	10
GLE2	Putative nuclear pore complex	383	42	5
GLG2	Putative self-glucosylating initiator of glycogen synthesis	361	42	5
GLK1	Putative glucokinase	472	52,1	18
GLN1	Putative glutamate synthase	373	41,7	8
GLN4	Putative tRNA-Gln synthetase	799	91,3	20
GLO1	Putative monomeric glyoxalase I	342	39,5	5
GLO2	Protein of unknown function	268	30,7	4

GLO3	Putative ARF GTPase activator	451	49,3	6
GLR1	Glutathione reductase	516	56,4	13
GLT1	Putative glutamate synthase	2126	235,2	44
GLX3	Glutathione-independent glyoxalase	236	25,8	9
GND1	6-phosphogluconate dehydrogenase	495	54,3	30
GPA2	G-protein alpha subunit	502	56,8	4
GPD2	Surface protein similar to glycerol 3-P dehydrogenase	371	40,8	7
GPH1	Putative glycogen phosphorylase	900	102,8	31
GPM1	Phosphoglycerate mutase	248	27,4	19
GRE3	Putative D-xylose reductase	371	42,4	12
GRP2	Methylglyoxal reductase	341	37,6	20
GRS1	Putative tRNA-Gly synthetase	652	73,7	25
GRX3	Putative glutaredoxin	253	28,4	4
GSC1	Essential beta-1,3-glucan synthase subunit	1897	217,9	6
GSP1	Small RAN G-protein	214	24,3	8
GST2	Glutathione S transferase	219	25,2	7
GTT11	Glutathione S-transferase, localized to ER	249	28,6	6
GUA1	Putative GMP synthase, involved in the final step of guanine biosynthesis	530	58,8	7
GUK1	Putative guanylate kinase	190	21,3	6
GUS1	Putative glutamine-tRNA ligase	725	82,5	15
GVP36	BAR domain protein	321	36,1	8
HAL21	Putative phosphoadenosine-5'-phosphate or 3'-phosphoadenosine 5'-phosphosulfate phosphatase	342	36,8	3
HAL22	Putative phosphoadenosine-5'-phosphate or 3'-phosphoadenosine 5'-phosphosulfate phosphatase	358	38,7	4
HAT1	Hat1-Hat2 histone acetyltransferase complex subunit	413	48,3	4
HAT2	Putative Hat1-Hat2 histone acetyltransferase complex subunit	382	42,8	3
HBR1	Essential protein involved in regulation of MTL gene expression	248	28,7	4
HBR2	Putative alanine glyoxylate aminotransferase	425	46,4	4
HCH1	Ortholog of S. cerevisiae Hch1, a regulator of heat shock protein Hsp90	152	17,7	3
HCR1	Putative translation initiation factor	285	32	3
HEM13	Coproporphyrinogen III oxidase	323	36,9	11
HEM2	Putative porphobilinogen synthase	341	37,1	6
HET1	Putative sphingolipid transfer protein	197	21,8	6
HHF1	Putative histone H4	105	11,6	4
HHO1	Putative histone H1	180	18,5	4
HIS1	ATP phosphoribosyl transferase	298	32,6	10
HIS4	Multifunctional enzyme that catalyzes three steps of histidine biosynthesis, with phosphoribosyl-AMP cyclohydrolase, phosphoribosyl-ATP diphosphatase, and histidinol dehydrogenase activities	838	91,9	12
HIS5	Putative histidinol-phosphate aminotransferase	389	43,1	5
HMG1	HMG-CoA reductase	1073	116,3	3
HMO1	HMG-box transcription factor	223	24,8	4
HNT1	Protein with similarity to protein kinase C inhibitor-I	152	17	2
HOM2	Aspartate-semialdehyde dehydrogenase	365	39,2	11
HOM3	Putative L-aspartate 4-P-transferase	544	59,6	9
HOM6	Putative homoserine dehydrogenase	359	38,8	16
HPD1	3-hydroxypropionate dehydrogenase	354	39	4
HPT1	Putative hypoxanthine-guanine phosphoribosyltransferase	213	24,2	5
HRT2	Protein described as having a role in Ty3 transposition	452	52,7	6
HSP104	Heat-shock protein	899	99,9	34

HSP12	Heat-shock protein	127	13,3	1
HSP21	Small heat shock protein	189	21,5	12
HSP60	Heat shock protein	566	60,1	37
HSP70	Putative hsp70 chaperone	656	70,3	23
HSP78	Heat-shock protein	812	91,8	22
HSP90	Essential chaperone, regulates several signal transduction pathways and temperature-induced morphogenesis	707	80,8	54
HTA1	Histone H2A	132	14	1
HTA2	Putative histone H2A	131	13,8	1
HTA3	Putative histone H2A	133	14,4	1
HTS1	Putative tRNA-His synthetase	501	55,6	9
HXK2	Hexokinase II	484	53,4	32
ICL1	Isocitrate lyase	550	61,4	24
IDH1	Putative mitochondrial NAD-isocitrate dehydrogenase subunit 1	364	39,2	17
IDH2	Putative mitochondrial NAD-isocitrate dehydrogenase subunit	369	39,8	17
IDI1	Protein of unknown function	284	32,3	6
IDP1	Putative isocitrate dehydrogenase	433	48,5	20
IDP2	Isocitrate dehydrogenase	412	46,3	20
IFD6	Aldo-keto reductase	344	39,2	1
IFE2	Putative alcohol dehydrogenase	435	48,5	5
IFG3	Putative D-amino acid oxidase	347	38,7	9
IFR2	Zinc-binding dehydrogenase	358	38,5	10
ILS1	Putative isoleucyl-tRNA synthetase, the target of drugs including the cyclic beta-amino acid icofungipen/PLD-118/BAY-10-8888 and mupirocin	1088	125,2	30
ILV2	Putative acetolactate synthase	683	74,3	14
ILV3	Dihydroxyacid dehydratase	589	62,8	8
ILV5	Ketol-acid reductoisomerase	400	44,9	13
IMH3	Inosine monophosphate (IMP) dehydrogenase	521	56,2	14
INO1	Inositol-1-phosphate synthase	520	57,7	32
IPP1	Putative inorganic pyrophosphatase	288	32,1	14
ISA1	Putative mitochondrial iron-sulfur protein	269	29,8	2
IST2	Protein of unknown function	952	106,9	10
KAR2	Similar to Hsp70 family chaperones	687	74,5	38
KEL1	Kelch repeat domain-containing protein	1018	113,9	4
KGD1	Putative 2-oxoglutarate dehydrogenase	996	112	34
KGD2	Putative dihydrolipoamide S-succinyltransferase	441	48	11
KIS1	Snf1p complex scaffold protein	412	46,7	3
KRE30	YEF3-subfamily ABC family protein	609	68,1	6
KRE9	Protein of beta-1,6-glucan biosynthesis	271	29,1	3
KRS1	Putative tRNA-Lys synthetase	594	67,7	17
LAG1	Putative ceramide synthase component	412	48,6	3
LAP3	Putative aminopeptidase	509	58,2	7
LAP41	Putative aminopeptidase yscI precursor	517	56,7	13
LAT1	Putative dihydrolipoamide acetyltransferase component (E2) of pyruvate dehydrogenase complex	477	50	14
LEU1	3-isopropylmalate dehydratase	776	86	19
LEU2	Isopropyl malate dehydrogenase	373	40,1	11
LEU4	Putative 2-isopropylmalate synthase	579	64,2	8
LEU42	Putative alpha-isopropylmalate synthase	571	63,4	10
LHP1	Protein of unknown function	324	37,5	10
LHS1	Protein similar to S. cerevisiae Hsp70p	932	104,8	11

LIG1	tRNA ligase	832	95,5	5
LKH1	Putative leukotriene A(4) hydrolase	623	71,4	7
LPD1	Putative dihydrolipoamide dehydrogenase	491	52,8	10
LPG20	Aldo-keto reductase family protein	348	39,9	2
LSC1	Putative succinate-CoA ligase subunit	323	33,7	10
LSC2	Putative succinate-CoA ligase beta subunit	415	44,4	21
LSP1	Eisosome component with a predicted role in endocytosis	317	35,6	15
LYS1	Saccharopine dehydrogenase (biosynthetic)	382	42,4	10
LYS12	Homoisocitrate dehydrogenase	376	40,6	17
LYS2	Heterodimeric alpha-aminoadipate reductase large subunit	1404	155,2	17
LYS21	Homocitrate synthase	428	47,5	5
LYS22	Putative homocitrate synthase	417	46,2	6
LYS4	Homoaconitase	684	74,4	10
LYS9	Saccharopine dehydrogenase	444	49,2	11
MAE1	Malic enzyme, mitochondrial	630	69,7	8
MAM33	Putative mitochondrial acidic matrix protein	264	29,9	6
MBF1	Putative transcriptional coactivator	151	16,3	10
MCI4	Putative NADH-ubiquinone dehydrogenase	139	15,6	2
MCR1	NADH-cytochrome-b5 reductase	301	33,4	12
MDG1	Protein of unknown function	660	70,5	21
MDH1	Mitochondrial malate dehydrogenase	337	36	14
MDH1-1	Predicted malate dehydrogenase precursor	332	34,7	18
MDH1-3	Predicted malate dehydrogenase	342	35,8	5
MDJ1	Putative member of the HSP40 (DnaJ) family of chaperones	488	52,9	3
MED1	RNA polymerase II mediator complex subunit	618	69,7	2
MED17	Putative RNA polymerase II mediator complex subunit	565	65,6	2
MES1	Cytoplasmic methionyl-tRNA synthetase	748	84,9	12
MET10	Sulfite reductase	1094	121,9	7
MET13	Putative methionine biosynthesis protein	614	69,9	3
MET14	Putative adenylylsulfate kinase	200	22,5	5
MET15	O-acetylhomoserine O-acetylserine sulfhydrylase	440	48	23
MET2	Homoserine acetyltransferase	409	45,4	2
MET6	Essential 5-methyltetrahydropteroyltriglutamate-homocysteine methyltransferase (cobalamin-independent methionine synthase)	767	85,6	48
MEU1	Putative methylthioadenosine phosphorylase	344	37,6	6
MIA40	Predicted component of the mitochondrial intermembrane space import machinery	252	27,9	3
MIR1	Putative mitochondrial phosphate transporter	307	32,5	11
MIS11	Predicted mitochondrial C1-tetrahydrofolate synthase precursor	946	102,1	26
MIS12	Mitochondrial C1-tetrahydrofolate synthase precursor	1025	111,2	8
MLC1	Microtubule-dependent localized protein	143	16	6
MLP1	Protein of unknown function	1832	207,6	13
MLS1	Malate synthase	551	62,5	29
MLT1	Vacuolar membrane transporter	1606	180,6	9
MMD1	Mitochondrial protein	156	17	5
MP65	Cell surface mannoprotein	378	39,2	4
MRF1	Putative mitochondrial respiratory protein	359	38,6	8
MRP2	Protein similar to S. cerevisiae Mrp2p, which is a component of the small subunit of the mitochondrial ribosome	115	13,2	2
MRP20	Component of mitochondrial ribosome	293	34,3	4
MRP7	Mitochondrial ribosomal protein of the large subunit	389	44,5	5
MRP8	Mitochondrial ribosomal protein	205	23,4	3

MRPL33	Putative mitochondrial ribosomal protein of the large subunit	96	11,2	2
MRPL40	Putative mitochondrial ribosomal protein	338	38,9	3
MRPL8	Mitochondrial 60S ribosomal protein subunit	255	29,4	3
MRS7	Member of the LETM1-like protein family, mitochondrial membrane protein	508	58,1	7
MSI3	Essential HSP70 family protein	701	78,5	33
MSN5	Predicted karyopherin involved in nuclear import and export of proteins	1245	142,4	7
MSO1	Putative secretory protein involved in S. cerevisiae sporulation	169	19,3	2
MTR10	Putative importin	959	110,2	4
MVD	Mevalonate diphosphate decarboxylase	362	39,5	13
MXR1	Putative methionine sulfoxide reductase	185	21,1	6
MYO5	Class I myosin	1316	146,8	2
NAB3	Putative nuclear polyadenylated RNA-binding protein	836	92,1	4
NAD1	Subunit 6 of NADH:ubiquinone oxidoreductase (NADH:ubiquinone dehydrogenase), a multisubunit enzyme complex (complex I) of the mitochondrial inner membrane that catalyzes the first step in mitochondrial respiration	317	34,8	2
NAP1	Nucleosome assembly protein	435	49,5	4
NAT2	Putative N-terminal acetyltransferase	212	23,8	2
NCE103	Carbonic anhydrase	281	31,6	3
NCP1	NADPH-cytochrome P450 reductase, acts with Erg11p in sterol 14 alpha-demethylation in ergosterol biosynthesis	680	76,6	8
NDH51	Nicotinamide adenine dinucleotide dehydrogenase complex I subunit of the mitochondrial electron transport chain	490	53,8	4
NHP6A	Putative non-histone chromatin component	92	10,5	4
NIF3	Protein of unknown function	280	31	5
NIP1	Putative translation initiation factor	874	99,8	13
NIT3	Putative nitrilase	301	32,9	6
NMD3	Putative nonsense-mediated mRNA decay protein	520	59,2	5
NMD5	Karyopherin	1017	116,1	3
NMT1	Myristoyl-CoA:protein N-myristoyltransferase	451	51,8	4
NOT5	Protein with similarity to S. cerevisiae Not5p, a member of the transcription regulatory CCR4-NOT complex	662	74,7	5
NPT1	Putative nicotinate phosphoribosyltransferase, involved in NAD salvage pathway	421	48,3	6
NSP1	Essential component of the nuclear pore complex	718	74,7	10
NTF2	Putative nuclear envelope protein	124	14,2	4
NUC2	Putative NADH-ubiquinone oxidoreductase	478	53,9	4
OBPA	Similar to oxysterol binding protein	433	49,5	2
OFD1	Putative prolyl hydroxylase family member	617	70,8	6
OFR1	Protein of unknown function	135	15,4	4
orf19.105	Predicted histone H2B	130	14,1	6
orf19.105	Protein of unknown function	269	30,5	3
orf19.105	Protein of unknown function	277	31,4	3
orf19.108	Protein of unknown function	83	9,7	5
orf19.108	Protein of unknown function	622	67	9
orf19.115	Protein of unknown function	265	30,6	2
orf19.117	Protein of unknown function	530	58,7	4
orf19.121	Protein of unknown function	447	50,3	3
orf19.122	Protein of unknown function	1045	119,1	7
orf19.126	Protein of unknown function	461	52,6	2
orf19.130	Protein of unknown function	304	34	2
orf19.133	Protein of unknown function	364	41,1	4
orf19.134	Putative aldose reductase	309	35,2	5
orf19.135	Putative protein of unknown function	347	38,6	11

orf19.135	Protein of unknown function	583	67,6	3
orf19.137	Protein of unknown function	190	21,4	6
orf19.138	Protein of unknown function	163	18,5	6
orf19.139	Putative protein of unknown function	166	18,7	4
orf19.139	Protein of unknown function	338	36,2	4
orf19.140	Ribosomal 60S subunit protein L22B	124	14	3
orf19.144	Protein of unknown function	522	60	6
orf19.146	Putative glutamine-dependent NAD synthetase, involved in NAD salvage pathway	714	81,1	7
orf19.154	Putative cis-golgi localized protein involved in ER to Golgi transport	265	29,8	6
orf19.154	Membrane-localized protein of unknown function	292	34,7	3
orf19.1549	Plasma membrane-associated protein identified in detergent-resistant membrane fraction (possible lipid raft component)	248	29,2	8
orf19.156	Plasma membrane-localized protein of unknown function	189	21,4	5
orf19.156	Protein of unknown function	613	69,5	2
orf19.1578	Ortholog of S. cereviaie Rrp5, an RNA binding protein involved in synthesis of 18S and 5.8S rRNAs	1722	193,2	2
orf19.1642	Ortholog of S. cerevisiae Loc1, a nuclear protein involved in asymmetric localization of ASH1 mRNA in S. cerevisiae	206	23,8	2
orf19.166	Protein of unknown function	351	39,9	2
orf19.167	Alpha subunit of COPI vesicle coatomer complex	1221	138,1	9
orf19.168	Membrane protein	386	43,3	7
orf19.169	Protein of unknown function	353	40,4	6
orf19.170	Sterol carrier domain protein	127	13,8	4
orf19.172	Protein of unknown function	222	24,2	2
orf19.173	HMG-box protein	311	34,6	3
orf19.177	Protein of unknown function	1382	159,7	4
orf19.178	Protein with a PI31 proteasome regulator domain	326	35,9	5
orf19.185	Protein of unknown function	368	41	6
orf19.186	Possible stress protein	121	13,1	4
orf19.189	Protein of unknown function	488	55,3	2
orf19.194	Protein of unknown function	246	27,5	3
orf19.194	Similar to an aldose 1-epimerase-related protein	299	33,8	9
orf19.195	Predicted protein of unknown function	370	41,3	3
orf19.196	Protein of unknown function	180	21	2
orf19.197	Putative vacuole biogenesis protein	234	24,9	2
orf19.199	Protein of unknown function	413	48,3	5
orf19.200	Putative nuclear RNA-binding protein	193	22	9
orf19.204	Protein of unknown function	636	72,1	2
orf19.205	Protein of unknown function	846	95,5	2
orf19.206	Protein of unknown function	342	38,2	2
orf19.211	Putative integral peroxisomal membrane protein	462	51,8	2
orf19.212	Predicted alcohol dehydrogenase	356	38,5	7
orf19.212	Protein of unknown function	151	17,4	4
orf19.213	Protein of unknown function	277	32,1	5
orf19.2150	Putative ortholog of mammalian electron transfer flavoprotein complex subunit ETF-alpha	329	34,3	5
orf19.216	Protein of unknown function	173	19,3	4
orf19.221	Protein of unknown function	287	32,8	3
orf19.224	Similar to oxidoreductases and to S. cerevisiae Yjr096wp	319	36,3	3
orf19.225	Protein of unknown function	230	26,1	2
orf19.225	Predicted ER protein involved in ER-nucleus signaling	367	42,5	2

orf19.226	Protein similar to quinone oxidoreductases	384	42,9	4
orf19.226	Protein of unknown function	469	53,6	2
orf19.226	Putative 3-phosphoserine phosphatase	245	27,7	6
orf19.227	Putative mitochondrial ribosomal protein	174	18,7	6
orf19.229	Predicted mucin-like protein	2551	264,3	55
orf19.230	Protein similar to S. cerevisiae Gvp36p	342	38,4	10
orf19.230	Protein of unknown function	124	14,3	4
orf19.233	Putative aspartyl aminopeptidase	496	55,1	4
orf19.239	Putative ATP-dependent helicase	1303	149,8	3
orf19.241	Ortholog of S. cerevisiae Mpm1	322	37,1	10
orf19.247	Putative RSC chromatin remodeling complex component	634	71,8	2
orf19.247	60S ribosomal protein L7	241	27,4	14
orf19.248	Putative karyopherin beta	1109	122,1	11
orf19.25	Protein of unknown function	323	37,7	2
orf19.253	Protein with a predicted role in protein translocation from the endoplasmic reticulum	95	9,9	4
orf19.263	Protein of unknown function	173	20,6	3
orf19.266	Protein of unknown function	899	102,2	2
orf19.269	Putative mitochondrial GTPase	897	99,9	3
orf19.271	Protein of unknown function	214	24,6	3
orf19.2711	Similar to S. cerevisiae Elp2, an Elongator complex subunit required for modification of wobble nucleosides in tRNA	797	89	2
orf19.272	Cytosolic chaperonin Cct ring complex subunit	544	59,3	7
orf19.273	Carbohydrate kinase domain-containing protein	742	81,1	4
orf19.275	Protein of unknown function	245	27,3	8
orf19.276	Putative protease B inhibitor	127	13,6	5
orf19.282	Protein of unknown function	175	19,9	6
orf19.285	Ortholog of S. cerevisiae Igo2	129	14,4	4
orf19.287	Putative NADH-ubiquinone oxidoreductase subunit	194	21,5	4
orf19.293	Predicted translation initiation factor	648	71,5	9
orf19.295	Protein of unknown function	200	22,6	3
orf19.296	Protein of unknown function	795	89,8	3
orf19.302	Protein of unknown function	310	35,8	3
orf19.303	Putative poly(A)-binding protein	629	70,4	22
orf19.305	Protein of unknown function	181	20,3	8
orf19.312	Putative chromatin remodelling complex protein	458	50	4
orf19.313	Putative NADP-dependent oxidoreductase	372	40,4	3
orf19.314	Protein of unknown function	906	97,8	5
orf19.317	Putative ortholog of human electron transfer flavoprotein dehydrogenase (ETF-dH)	627	69,6	3
orf19.323	Putative F-actin capping protein subunit alpha	280	32	4
orf19.326	Protein of unknown function	731	80,3	2
orf19.329	Plasma membrane-localized protein	156	17,8	3
orf19.331	Protein of unknown function	250	27,3	2
orf19.331	Protein of unknown function	624	71,4	9
orf19.331	Putative thioredoxin	327	36,9	4
orf19.333	Plasma membrane protein of unknown function	200	21,8	12
orf19.334	Putative tRNA-Arg synthetase	622	70,8	18
orf19.334	Putative RNA polymerase II subunit B150	1234	139,8	8
orf19.335	Protein of unknown function	262	29,4	18
orf19.336	Protein of unknown function	152	17,8	3
orf19.338	Putative glycoside hydrolase	1047	121,2	18

orf19.344	Putative oxidoreductase	413	47	2
orf19.346	Putative GTPase	684	77,8	2
orf19.347	Described as a Gag-related protein	186	19,6	14
orf19.348	Protein of unknown function	483	54,6	5
orf19.348	Protein of unknown function	463	52,9	4
orf19.350	Putative protein of unknown function	377	42	6
orf19.351	Protein of unknown function	286	32	5
orf19.355	Protein of unknown function	213	24,6	2
orf19.355 9	S. cerevisiae ortholog Mrps35p is a structural constituent of ribosome and localizes to mitochondrial small ribosomal subunit	314	36,2	9
orf19.357	Protein of unknown function	283	30,8	4
orf19.357	Ribosomal 60S subunit protein L31B	112	13	11
orf19.358	Protein of unknown function	385	43,1	6
orf19.36.	Protein of unknown function	68	7,6	6
orf19.361	Protein of unknown function	157	17,8	4
orf19.364	Putative vacuolar H+-ATPase subunit	348	40,1	3
orf19.366	Putative deubiquitinating enzyme	824	94,6	2
orf19.368	Protein of unknown function	875	96,5	5
orf19.368	Putative oxidoreductase	292	30,6	2
orf19.369	Ribosomal 60S subunit protein	127	14,2	9
orf19.379	Protein of unknown function	258	29,1	2
orf19.379	Protein of unknown function	366	38,7	8
orf19.385	Putative microsomal beta-keto-reductase	408	45,2	4
orf19.391	Putative metallodipeptidase	434	47,9	10
orf19.393	Predicted RNA binding protein	275	30,3	13
orf19.394	Protein of unknown function	1314	148,4	2
orf19.397	Protein of unknown function	147	17,2	4
orf19.399	Putative serine/threonine protein kinase	712	79,3	2
orf19.401	Putative ubiquinol-cytochrome-c reductase	439	47,9	9
orf19.409	Ortholog of S. cerevisiae Aim38/Rcf2, cytochrome c oxidase subunit	213	24,3	3
orf19.410	Exopolyphosphatase, hydrolyzes inorganic polyphosphate (poly P) into Pi residues	401	45,9	4
orf19.412	Putative THO complex subunit	1608	183,6	3
orf19.414	Protein component of the small (40S) ribosomal subunit	155	17,6	9
orf19.417	Protein of unknown function	246	28,9	4
orf19.420	Protein of unknown function	301	35,2	6
orf19.421	Putative heat shock protein	127	13,3	1
orf19.424	Protein with similarity to S. cerevisiae Ykr070w	597	66,3	13
orf19.424	Ortholog of S. cerevisiae YLR118C (alias Apt1)	301	32,7	4
orf19.425	Protein of unknown function	781	87,9	3
orf19.426	Protein of unknown function	684	79,4	5
orf19.427	Protein of unknown function	669	77,4	5
orf19.428	Protein of unknown function	335	37,2	2
orf19.430	Protein of unknown function	270	30,6	3
orf19.434	Protein of unknown function	127	14,7	3
orf19.434	Protein of unknown function	2091	225,9	4
orf19.439	Putative actin cytoskeleton component	613	67,2	11
orf19.439	Mitochondrial inner membrane protein	567	62,8	7
orf19.445	Protein conserved among the CTG-clade	68	7,9	3
orf19.447	Protein with a NADP-dependent oxidoreductase domain	344	39,1	2

orf19.4488	Predicted ortholog of S. cerevisiae Swi3, subunit of the SWI/SNF chromatin remodeling complex	971	109,3	5
orf19.449	Putative phosphatidyl synthase	398	44,8	5
orf19.451	Protein of unknown function	410	46,5	3
orf19.453	Protein of unknown function	305	34	2
orf19.454	Protein of unknown function	239	26,9	2
orf19.457	Protein of unknown function	146	16,8	3
orf19.460	Putative dienelactone hydrolase	243	26,7	9
orf19.462	Protein of unknown function	385	41,2	5
orf19.463	Protein of unknown function	268	29,1	6
orf19.475	Protein of unknown function	295	34,2	5
orf19.475	Putative reductase or dehydrogenase	246	27,7	2
orf19.479	Putative eIF-4E-binding repressor of CAP-dependent translation	176	20	5
orf19.487	Protein of unknown function	848	93,3	6
orf19.489	Putative protein of unknown function	229	24,3	6
orf19.493	Putative tRNA-Cys synthetase	781	89,7	17
orf19.493	Protein of unknown function	761	84,3	9
orf19.494	Mitochondrial membrane protein of unknown function	169	18,6	3
orf19.495	Putative ATPase	298	34,3	5
orf19.495	Protein of unknown function	573	64	4
orf19.498	Putative mitochondrial ribosomal component of the small subunit	461	53,4	5
orf19.502	C2H2 transcription factor	1177	133,1	3
orf19.505	Putative quinolinate phosphoribosyl transferase, involved in NAD biosynthesis	300	32,4	8
orf19.507	Protein of unknown function	166	18,9	2
orf19.509	Putative oxysterol-binding protein	1318	148,3	6
orf19.51	Protein of unknown function	1363	155,7	16
orf19.511	Protein of unknown function	63	7,2	2
orf19.512	Putative adhesin-like protein	397	43,2	7
orf19.518	Protein of unknown function	713	81,2	3
orf19.518	Putative Ran guanyl-nucleotide exchange factor	493	54,1	4
orf19.519	Putative protein of unknown function	253	27,4	4
orf19.520	Protein of unknown function	83	9,6	8
orf19.523	Putative mitochondrial ribosomal protein of the large subunit	232	27,7	4
orf19.523	Predicted alanine-tRNA ligase	471	52,2	3
orf19.525	Protein of unknown function	162	17,9	3
orf19.528	Predicted essential RNA-binding G protein	1220	133,9	33
orf19.529	Protein of unknown function	507	57,8	14
orf19.532	Protein of unknown function	640	71,4	4
orf19.536	Uroporphyrinogen decarboxylase	361	40,6	5
orf19.551	Protein of unknown function	326	37,9	2
orf19.551	Protein of unknown function	635	71,6	5
orf19.555	Protein of unknown function	175	19,3	3
orf19.559	Protein of unknown function	63	6,7	3
orf19.560	Protein of unknown function	313	36,7	2
orf19.560	RNA polymerase III subunit	330	38,4	2
orf19.561	Protein of unknown function	652	74,8	2
orf19.562	Stationary phase enriched protein	309	33,8	6
orf19.5627	S. cerevisiae ortholog Hek2/Khd1 is a putative RNA binding protein involved in the asymmetric localization of ASH1 mRNA	368	40,6	5
orf19.566	Protein of unknown function	93	10,4	4
orf19.566	Protein of unknown function	146	16,6	5

orf19.566	Protein with B-cell receptor-associated protein 31-like domain	186	21,4	2
orf19.567	Protein of unknown function	811	90,1	7
orf19.568	Protein of unknown function	200	22,9	2
orf19.568	Epsilon-COP subunit of the coatomer	311	35,6	3
orf19.571	Nucleoporin component of central core of the nuclear pore complex	1161	123	2
orf19.574	Protein of unknown function	460	52	7
orf19.577	Putative dipeptidyl-peptidase III	700	79,7	19
orf19.581	Putative RNA-binding protein	698	75,4	6
orf19.582	Protein of unknown function	717	81,9	3
orf19.583	Protein of unknown function	363	40,4	3
orf19.583	Protein of unknown function	1002	114,4	3
orf19.585	Protein of unknown function	283	33,2	3
orf19.591	RNA binding protein required for export of poly(A)+ mRNA from the nucleus	227	24	5
orf19.592	Protein of unknown function	363	39,5	3
orf19.594	Protein of unknown function	267	29,1	16
orf19.596	Protein of unknown function	249	27,3	5
orf19.603	Protein of unknown function	121	14,7	3
orf19.603	Protein of unknown function	337	38,2	2
orf19.606	RNA polymerase II holoenzyme/mediator subunit	323	37,3	3
orf19.606	Hexadecenal dehydrogenase	542	60,6	6
orf19.6090	Putative nucleolar protein with a predicted role in pre-rRNA processing and ribosome biogenesis	400	43,5	3
orf19.613	Protein of unknown function	127	14,8	2
orf19.616	Protein of unknown function	816	89,6	26
orf19.617	Protein of unknown function	303	34,2	4
orf19.622	Ribosomal 60S subunit protein	122	13,7	3
orf19.631	Protein of unknown function	101	11,4	3
orf19.635	Protein of unknown function	137	15,6	3
orf19.641	Protein of unknown function	56	6,6	4
orf19.645	Protein of unknown function	1313	151,4	2
orf19.647	Protein of unknown function	426	49,1	2
orf19.650	Protein of unknown function	153	17,8	3
orf19.650	Putative curved DNA-binding protein orthologous to S. pombe Cdb4	387	43,1	12
orf19.655	Regulator of calcineurin	275	31,5	2
orf19.655	RNA polymerase III transcription initiation factor complex (TFIIIC) subunit	485	54,3	11
orf19.656	Conserved mitochondrial inner membrane insertase	374	41,3	2
orf19.659	Putative esterase	303	34,2	9
orf19.660	Protein of unknown function	204	23,9	2
orf19.661	Putative mitochondrial protein	266	29,6	6
orf19.662	Protein of unknown function	275	32,4	2
orf19.666	Protein of unknown function	792	84,3	4
orf19.670	Protein with similarity to amino acid-tRNA ligase	691	77,3	22
orf19.673	Protein of unknown function	89	9,6	5
orf19.673	Protein of unknown function	616	69,3	4
orf19.674	Protein of unknown function	328	37,4	2
orf19.675	Protein of unknown function	234	27,4	3
orf19.680	Protein of unknown function	606	70	2
orf19.680	Putative phosphomutase-like protein	322	37,3	11
orf19.681	Protein of unknown function	643	73,1	10
orf19.682	Protein of unknown function	296	34,9	2

orf19.6838	Putative protein of unknown function, transcript upregulated in clinical isolates from HIV+ patients with oral candidiasis	269	28,8	2
orf19.685	Protein of unknown function	345	39,3	6
orf19.686	Protein with a predicted cytochrome b5-like heme/steroid binding domain	129	14,9	4
orf19.688	Ribosomal 60S subunit protein	107	12,1	2
orf19.688	Putative oxysterol binding protein family	430	48,2	4
orf19.688	Predicted ORF from Assembly 19	110	13,1	3
orf19.689	Protein of unknown function	453	51,6	4
orf19.689	Protein of unknown function	92	10,4	2
orf19.701	Protein of unknown function	1217	135,8	3
orf19.703	Putative eIF4E-associated protein	768	84,4	2
orf19.706	Protein of unknown function	1091	123,7	3
orf19.708	Protein of unknown function	439	50,4	5
orf19.709	Putative cytoplasmic RNA-binding protein	187	20,5	4
orf19.711	Protein of unknown function	317	37,2	4
orf19.713	Protein of unknown function	138	15,3	4
orf19.714	Putative catechol o-methyltransferase	248	28,2	5
orf19.7152	Protein similar to Aspergillus CYSK O-acetylserine sulfhydrylase, suggesting that C. albicans uses an O-acetyl-serine (OAS) pathway of sulfur assimilation	365	39,3	7
orf19.716	Protein of unknown function	212	23,8	2
orf19.717	Protein of unknown function	638	70,5	6
orf19.719	Putative vacuolar protease	493	52,1	6
orf19.721	Nucleolar protein	1818	204,2	2
orf19.721	Protein of unknown function	106	11,5	7
orf19.723	Putative RSC chromatin remodeling complex component	561	64,3	5
orf19.724	Putative fumarylacetoacetate hydrolase	231	25,7	4
orf19.725	Protein of unknown function	147	16,8	4
orf19.726	Metalloprotease subunit of the 19S regulatory particle of the 26S proteasome lid	312	34,9	4
orf19.726	Putative polyamine acetyltransferase	220	24,8	7
orf19.728	Protein with predicted oxidoreductase and dehydrogenase domains	440	49,3	7
orf19.729	Putative cystathionine gamma-synthase	383	42,4	9
orf19.730	Aldo-keto reductase	349	39	13
orf19.731	Protein with a role in directing meiotic recombination events to homologous chromatids	788	91,5	4
orf19.732	Protein of unknown function	147	17,2	4
orf19.732	Protein with a Staphylococcal nuclease domain	901	100	11
orf19.735	Protein of unknown function	348	40,1	6
orf19.736	Protein of unknown function	510	56,6	2
orf19.740	Catabolic L-serine (L-threonine) deaminase	332	36,1	2
orf19.748	Putative mitochondrial ribosomal protein of the large subunit	293	32	3
orf19.750	Protein of unknown function	258	29,5	9
orf19.753	Protein of unknown function	229	25,2	8
orf19.759	Putative NADH-ubiquinone oxidoreductase	720	79,3	13
orf19.767	Mitochondrial ribosomal protein of the large subunit	158	18,5	2
orf19.804	Protein of unknown function	76	8,7	2
orf19.86	Putative glutathione peroxidase	161	18,1	7
orf19.863	Protein of unknown function	378	43,9	4
orf19.909	Protein of unknown function	74	8	2
orf19.92	Protein with a predicted thioredoxin-like domain	896	99,9	28
orf19.94	Protein of unknown function	244	26,4	6
orf19.969	Protein of unknown function	404	44,4	5
ORM1	Putative endoplasmic reticulum membrane protein	268	29,6	2

OSM1	Putative flavoprotein subunit of fumarate reductase	503	54,3	18
OSM2	Putative mitochondrial fumarate reductase	648	70,4	8
OYE23	Putative NAPDH dehydrogenase	406	46	1
OYE32	NAD(P)H oxidoreductase family protein	432	47,5	8
PAN1	Essential protein involved in endocytosis and polarized growth	1397	152,4	11
PBP2	Putative RNA binding protein	529	58,4	4
PCK1	Phosphoenolpyruvate carboxykinase	553	60,9	9
PDA1	Putative pyruvate dehydrogenase alpha chain	401	44,1	21
PDB1	Putative pyruvate dehydrogenase	379	41,3	13
PDC11	Pyruvate decarboxylase	567	62,4	24
PDI1	Putative protein disulfide-isomerase	560	63	28
PDK2	Putative pyruvate dehydrogenase kinase	511	58	3
PDX1	Pyruvate dehydrogenase complex protein X	417	46	12
PDX3	Pyridoxamine-phosphate oxidase	269	31,3	3
PEP1	Type I transmembrane sorting receptor for multiple vacuolar hydrolases	1339	150,4	4
PEP8	Protein similar to S. cerevisiae Pep8p, which is involved in retrograde transport	347	39,4	2
PET9	Mitochondrial ADP/ATP carrier protein involved in ATP biosynthesis	301	32,7	15
PEX11	Putative peroxisomal membrane protein	245	28,4	3
PEX14	Protein of unknown function	452	49,6	3
PFK1	Phosphofructokinase alpha subinit	987	108,5	32
PFK2	Phosphofructokinase beta subunit	946	104,1	17
PFY1	Profilin	126	13,8	5
PGA4	GPI-anchored cell surface protein	451	49	6
PGA63	Component COPII vesicle coat	1265	136,3	23
PGI1	Glucose-6-phosphate isomerase	550	61,1	28
PGK1	Phosphoglycerate kinase	417	45,2	40
PGM2	Ortholog of S. cerevisiae Pgm2	560	61,8	14
PHB2	Prohibitin 2	303	34,1	4
PHO15	4-nitrophenyl phosphatase, possible histone H2A phosphatase	308	34,2	4
PHO85	Functional homolog of S. cerevisiae Pho85p, a cyclin-dependent kinase that regulates	332	38,1	3
PHO88	Protein with a role in phosphate transport	191	20,9	4
PHR2	Glycosidase	544	58,7	5
PIL1	Eisosome component	308	34,6	14
PIM1	ATP-dependent Lon protease	1078	120,8	4
PIN3	Putative SH3-domain-containing protein	285	31	5
PKC1	Protein kinase C	1097	125,2	2
PMA1	Plasma membrane H(+)-ATPase	895	97,6	23
PMI1	Phosphomannose isomerase	441	48,8	13
PMM1	Phosphomannomutase	252	29	19
PNG2	Putative peptide:N-glycanase	983	111,8	11
PNP1	Purine nucleoside phosphorylase	307	33,4	5
POB3	Protein involved in chromatin assembly and disassembly	538	60,9	5
POL30	Similar to proliferating cell nuclear antigen (PCNA)	259	29	4
POR1	Mitochondrial outer membrane porin	282	29,7	16
POX1-3	Predicted acyl-CoA oxidase	709	78,9	16
PR26	Protein with similarity to proteasomal 26S regulatory subunit of S. cerevisiae, H. sapiens, Methanobacterium thermoautotrophicum (Archaebacterium)	411	45,9	5
PRD1	Putative proteinase	702	81,7	11
PRE1	Putative beta 4 subunit of the 20S proteasome	196	22	7
PRE2	Putative proteasome beta-5 subunit	285	31,3	5

PRE3	Putative beta-1 proteasome subunit	214	23,3	6
PRE5	Alpha6 subunit of the 20S proteasome	283	31,4	11
PRE6	Putative alpha-4 subunit of the proteasome	250	27,4	8
PRE8	Putative alpha-2_sc subunit of proteasome	251	27,7	6
PRE9	Alpha3 (C9) subunit of the 20S proteasome	251	27,8	10
PRO2	Putative gamma-glutamyl phosphate reductase with a predicted role in proline biosynthesis	446	49,1	8
PRO3	Delta 1-pyrroline-5-carboxylate reductase	274	29	3
PRT1	Putative translation initiation factor eIF3	739	84,2	7
PRX1	Thioredoxin peroxidase	243	27,4	18
PSA2	Mannose-1-phosphate guanyltransferase	458	50,8	9
PST1	Putative 1,4-benzoquinone reductase	198	21,1	3
PST2	Putative NADH:quinone oxidoreductase	201	21,7	2
PST3	Putative flavodoxin	199	21,2	6
PTC1	Putative protein phosphatase of the Type 2C-related family (serine/threonine-specific), similar to S. cerevisiae Ptc1p	375	40,7	2
PTC2	Protein phosphatase of the Type 2C-related family (serine/threonine-specific) with a potential role in DNA damage checkpoint control	583	64,5	6
PTC5	Mitochondrial protein phosphatase of the Type 2C-related family (serine/threonine-specific), involved in drug response and cadmium tolerance	580	64,9	3
PTC7	Protein phosphatase, type 2C	350	38	3
PUP1	Putative beta 2 subunit of the 20S proteasome	272	29,4	3
PUP2	Alpha5 subunit of the 20S proteasome	255	27,7	7
PUP3	Putative beta 3 subunit of the 20S proteasome	206	22,6	5
PUT2	Putative delta-1-pyrroline-5-carboxylate dehydrogenase	595	65,7	11
PWP1	Putative rRNA processing protein	613	67,9	3
PYC2	Putative pyruvate carboxylase	1177	129,7	46
QCR2	Ubiquinol-cytochrome-c reductase	374	39,5	15
QCR7	Putative ubiquinol-cytochrome-c reductase, subunit 7	127	14,4	7
QCR8	Putative ubiquinol cytochrome c reductase	95	11,1	2
QCR9	Putative ubiquinol cytochrome c reductase	65	7,5	2
RAC1	G-protein of RAC subfamily	236	25,9	3
RAD23	S. cerevisiae Rad23 ortholog	417	43,8	2
RAS1	RAS signal transduction GTPase	291	32,5	6
RAT1	5'-->3' exoribonuclease	968	111,7	5
RBF1	Transcription factor	534	60,2	2
RBP1	Peptidyl-prolyl cis-trans isomerase	124	13,3	3
RCT1	Fluconazole-induced protein	191	20,5	9
RDI1	Putative rho GDP dissociation inhibitor	203	22,9	15
REG1	Putative protein phosphatase regulatory subunit	1011	110,7	4
RET2	Delta subunit of the coatomer complex (COPI)	573	62,8	5
RFA2	Putative DNA replication factor A	272	29,4	3
RGD1	GTPase activator protein	677	74,8	2
RHO1	Small GTPase of Rho family	198	22	4
RIA1	Putative translation elongation factor	1044	117,8	6
RIB4	Lumazine synthase (6,7-dimethyl-8-ribityllumazine synthase, DMRL synthase)	206	23,1	8
RIB5	Putative riboflavin synthase	237	25,7	8
RIM1	Putative single-stranded DNA-binding protein	143	16,2	9
RIP1	Putative ubiquinol cytochrome c-reductase	213	23,2	3
RNA1	Putative GTPase-activating protein	414	46	10
RNR21	Ribonucleoside-diphosphate reductase	413	47,4	7

RNR22	Putative ribonucleoside diphosphate reductase	394	45,7	2
RPA135	Putative RNA polymerase I subunit A135	1166	131,1	3
RPA190	Putative RNA polymerase I subunit A190	1665	186,2	3
RPG1A	Putative translation initiation factor	930	106	9
RPL10	Ribosomal protein L10	220	25,2	12
RPL10A	Predicted ribosomal protein	217	24,4	11
RPL11	Ribosomal protein	174	19,8	8
RPL12	Ribosomal protein L12, 60S ribosomal subunit	165	17,8	7
RPL13	Putative ribosomal subunit	202	23	12
RPL14	Ribosomal protein L14	131	14,7	10
RPL15A	Putative ribosomal protein	204	24,3	7
RPL16A	Ribosomal protein	200	22,6	12
RPL17B	Ribosomal protein L17	185	21	8
RPL18	Predicted ribosomal protein	186	20,8	9
RPL19A	Ribosomal protein L19	190	21,9	12
RPL2	Putative 60S ribosomal protein L2	254	27,3	11
RPL20B	Ribosomal protein L20	172	20,3	14
RPL21A	Putative ribosomal protein	160	18	7
RPL23A	Ribosomal protein	137	14,5	7
RPL24A	Predicted ribosomal protein	155	17,4	10
RPL25	Putative rRNA-binding ribosomal protein component of the 60S ribosomal subunit	142	15,8	7
RPL27A	Ribosomal protein L27	136	15,5	8
RPL28	Putative ribosomal protein	149	16,7	9
RPL3	Ribosomal protein, large subunit	389	43,9	22
RPL30	Ribosomal 60S subunit protein	106	11,5	5
RPL32	Component of the large (60S) ribosomal subunit	131	14,9	8
RPL35	Ribosomal protein	120	14,1	10
RPL37B	Ribosomal protein L37	90	10,1	4
RPL38	60S ribosomal ribosomal protein subunit	78	8,9	4
RPL39	Ribosomal protein L39	99	11,1	4
RPL42	Putative 60S ribosomal subunit protein	106	12,2	3
RPL43A	Putative ribosomal protein, large subunit	92	10,1	2
RPL4B	Ribosomal protein 4B	363	39,2	14
RPL5	Ribosomal protein	298	34,5	7
RPL6	Ortholog of S. cerevisiae ribosomal subunit, Rpl6B	176	19,8	13
RPL7	Ribosomal protein L7	295	33,9	2
RPL82	Predicted ribosomal protein	260	28,2	1
RPL8B	Predicted ribosomal protein	262	28,5	4
RPL9B	Ribosomal protein L9	191	21,7	10
RPN1	Putative 19S regulatory particle of the 26S proteasome	994	109,9	18
RPN10	Putative 19S regulatory particle of the 26S proteasome	279	30,4	4
RPN2	Putative 26S proteasome subunit	952	104,7	13
RPN3	Putative non-ATPase regulatory subunit of the 26S proteasome lid	481	54,9	7
RPN6	Putative 26S proteasome subunit	435	49,4	7
RPN7	Subunit of the proteasome regulatory particle	400	46,2	4
RPN8	Putative regulatory subunit of the 26S proteasome	330	36,7	6
RPO21	RNA polymerase II	1728	191,9	10
RPP0	Putative ribosomal protein	312	33,3	13
RPP1A	Conserved acidic ribosomal protein	106	11	2

RPP1B	Conserved acidic ribosomal protein, likely involved in regulation of translation elongation	108	10,8	2
RPP2A	Acidic ribosomal protein	108	10,9	5
RPS1	Putative ribosomal protein 10 of the 40S subunit	256	29	16
RPS10	Ribosomal protein S10	118	13,8	5
RPS12	Acidic ribosomal protein S12	143	15,7	3
RPS13	Putative ribosomal protein of the small subunit	151	16,9	9
RPS14B	Putative ribosomal protein	132	14	11
RPS15	Putative ribosomal protein	142	15,9	5
RPS16A	Putative 40S ribosomal subunit	142	15,7	15
RPS17B	Ribosomal protein 17B	137	15,7	5
RPS18	Predicted ribosomal protein	145	17	12
RPS19A	Putative ribosomal protein S19	145	16,1	12
RPS20	Putative ribosomal protein	119	13,3	11
RPS21	Protein component of the small (40S) subunit	249	26,9	11
RPS21B	Ribosomal protein S21	87	9,6	7
RPS22A	Predicted ribosomal protein	130	14,8	6
RPS23A	Putative ribosomal protein	145	16	4
RPS24	Predicted ribosomal protein	135	15,5	10
RPS25B	Ribosomal protein	105	11,6	6
RPS26A	Ribosomal protein	119	13,6	5
RPS27	Putative ribosomal protein	82	9	3
RPS3	Ribosomal protein S3	251	27,3	17
RPS30	Putative 40S ribosomal protein S30	63	7,1	3
RPS5	Ribosomal protein S5	225	25,3	11
RPS6A	Ribosomal protein 6A	236	27,1	11
RPS7A	Ribosomal protein S7	186	21,2	13
RPS8A	Small 40S ribosomal subunit protein	206	22,7	11
RPS9B	Predicted ribosomal protein	189	21,7	12
RPT1	Putative 26S proteasome regulatory subunit 7	444	49,8	5
RPT2	Putative ATPase of the 19S regulatory particle of the 26S proteasome	465	51,8	6
RPT6	Putative ATPase of the 19S regulatory particle of the 26S proteasome	401	44,9	7
RSR1	RAS-related protein	248	27,6	3
RVS161	Protein required for endocytosis	264	30,1	2
RVS167	SH3-domain- and BAR domain-containing protein involved in endocytosis	440	49,2	2
SAC6	Fimbrin	647	72,1	8
SAH1	S-adenosyl-L-homocysteine hydrolase	450	49	20
SAM2	S-adenosylmethionine synthetase	385	42,2	17
SAM51	Component of the SAM complex involved in mitochondrial protein import, involved in beta-barrel protein assembly	508	57,4	3
SAR1	Functional homolog of S. cerevisiae Sar1	190	21,5	5
SBA1	Similar to co-chaperones	221	23,9	12
SBP1	Similar to RNA binding proteins	282	31,9	15
SCL1	Proteasome subunit YC7alpha	247	27,4	8
SCO1	Putative copper transporter	301	33,8	2
SDH12	Succinate dehydrogenase	641	70,1	10
SDH2	Succinate dehydrogenase, Fe-S subunit	263	30,1	8
SDS22	Putative protein serine-threonine phosphatase	374	43	7
SEC13	Putative protein transport factor	298	33	5
SEC14	Essential protein	301	34,7	6
SEC2	Guanyl-nucleotide exchange factor for the small G-protein Sec4	751	85,3	3

SEC21	Protein of unknown function	936	104,2	9
SEC23	Putative GTPase-activating protein	762	85,6	8
SEC24	Protein with a possible role in ER to Golgi transport	928	102	11
SEC26	Secretory vesicles coatomer complex protein	952	107,1	7
SEC27	Protein of unknown function	939	106	11
SEC4	Small GTPase of Rab family	210	23,1	3
SEC61	ER protein-translocation complex subunit	479	52,5	3
SEC7	Putative guanine nucleotide exchange factor (GEF)	1839	208,9	4
SER1	Putative 3-phosphoserine aminotransferase	392	43	8
SER33	Predicted enzyme of amino acid biosynthesis	463	50,3	17
SES1	Seryl-tRNA synthetase	458	52,6	16
SFC1	Putative succinate-fumarate transporter	303	33,2	12
SFH5	Putative phosphatidylinositol transporter	320	36,6	3
SGT1	Putative co-chaperone protein with a predicted role in kinetochore assembly	413	47,3	6
SGT2	Putative small tetratricopeptide repeat (TPR)-containing protein	344	36,4	11
SHM1	Mitochondrial serine hydroxymethyltransferase	493	54,4	17
SHM2	Cytoplasmic serine hydroxymethyltransferase	470	52	15
SHP1	Regulator of the type 1 protein phosphatase Glc7p activity, involved in control of morphogenesis, progression through the cell cycle and response to DNA damage	371	41,4	15
SIK1	Putative U3 snoRNP protein	516	57,7	2
SIN3	Protein similar to S. cerevisiae Sin3p (transcriptional corepressor involved in histone deacetylase recruitment)	1411	159,9	7
SIS1	Putative Type II HSP40 co-chaperone	343	36,8	9
SKI3	Protein of unknown function	1400	159,1	3
SKP1	Putative subunit D of kinetochore protein complex CBF3	164	19	5
SLA2	Actin binding protein with roles in growth control and morphogenesis	1063	120,8	15
SLK19	Alkaline-induced protein of plasma membrane	1197	133,9	39
SLP2	Protein similar to stomatin mechanoreception protein	263	29,5	2
SMI1	Cell wall biosynthesis protein	620	68,9	5
SMT3	SUMO, small ubiquitin-like protein	102	11,1	6
SNF2	Protein involved in transcriptional regulation	1690	193,5	1
SNF7	ESCRT III complex protein	226	25,9	5
SNL1	Ribosome-associated protein predicted to function in protein synthesis	199	23,5	2
SNO1	Protein with a predicted role in pyridoxine metabolism	249	28,4	2
SNX4	Putative sorting nexin	630	71,8	2
SNZ1	Stationary phase protein	292	31,8	10
SOD1	Cytosolic copper- and zinc-containing superoxide dismutase	154	16,1	8
SOD2	Mitochondrial Mn-containing superoxide dismutase	234	26,2	5
SOL3	Putative 6-phosphogluconolactonase	259	28,5	14
SPA2	Protein involved in cell polarity, Spitzenkorper formation	1466	164,3	5
SPE3	Putative spermidine synthase	297	33,9	6
SPL1	Protein similar to S. cerevisiae Spl1p, which is involved in tRNA splicing	488	53,5	3
SPT5	Protein similar to S. cerevisiae Spt5p transcription elongation factor	956	105,8	5
SPT6	Putative transcription elongation factor	1401	162	3
SRB1	Essential GDP-mannose pyrophosphorylase	362	40	15
SRV2	Adenylate cyclase-associated protein	545	59,6	12
SSA2	HSP70 family chaperone	645	70	20
SSB1	HSP70 family heat shock protein	613	66,4	27
SSC1	Heat shock protein	648	69,7	41
SSD1	Protein with role in resistance to host antimicrobial peptides	1274	141,2	17
SSZ1	Putative HSP70 chaperone	540	58,5	12

STF2	Protein involved in ATP biosynthesis	89	10,5	6
STI1	Protein that interacts with Cdc37 and Crk1 in two-hybrid	589	66	36
STR2	Protein of unknown function	595	67,1	3
SUB2	Putative TREX complex component with a predicted role in nuclear mRNA export	433	49,1	10
SUI1	Putative translation initiation factor	109	12,3	3
SUI2	Translation initiation factor eIF2, alpha chain	300	33,9	5
SUP35	Translation factor eRF3	721	79,9	10
SVF1	Putative survival factor	382	43	4
SYS3	Protein similar to S. cerevisiae Sys3p	895	102,8	6
TAF14	Putative DNA-binding transcription factor	263	29,1	3
TAL1	Transaldolase	323	35,7	25
TBP1	Transcription initiation factor	238	26,6	2
TCP1	Chaperonin-containing T-complex subunit, induced by alpha pheromone in SpiderM medium	554	60	8
TDH3	NAD-linked glyceraldehyde-3-phosphate dehydrogenase	335	35,8	31
TEF2	Translation elongation factor 1-alpha	458	50	17
TFG1	Protein similar to S. cerevisiae Tfg1p, which is part of transcription factor TFIIF	608	69	3
TFP1	Subunit of vacuolar H+-ATPase	617	67,6	19
TFS1	Putative carboxypeptidase y inhibitor	259	29,3	8
THR4	Putative threonine synthase	510	57,7	12
THS1	Putative threonyl-tRNA synthetase	706	81,6	17
TIF	Translation initiation factor	397	44,6	20
TIF11	Translation initiation factor eIF1a	154	17,4	2
TIF3	Putative translation initiation factor	455	50,7	8
TIF34	Putative translation initiation factor eIF3, p39 subunit	350	38,2	8
TIF4631	Putative translation initiation factor eIF4G	1084	118,2	16
TIF5	Putative translation initiation factor	420	46,6	8
TIM10	Predicted protein of the mitochondrial intermembrane space with role in protein import into mitochondrial inner membrane	91	10,1	4
TIM23	Protein involved in mitochondrial matrix protein import	218	23,1	2
TIM50	Predicted component of the Translocase of the Inner Mitochondrial membrane (TIM23 complex)	469	54,2	7
TIP120	Protein similar to human CAND1 (Cullin-Associated Nedd8-Dissociated) protein involved in regulation of SCF complexes	1195	134,4	8
TKL1	Putative transketolase	677	73,7	27
TMA19	Cell wall protein, ortholog of S. cerevisiae Tma19p (Ykl065cp)	167	18,5	8
TOM1	Putative E3 ubiquitin ligase	3286	375,3	4
TOM20	Putative mitochondrial primary import receptor	171	19	3
TOM22	Putative mitochondrial import receptor subunit	150	16,6	3
TOM40	Protein involved in mitochondrial protein import	390	42,3	3
TPI1	Triose-phosphate isomerase	248	26,6	14
TPK1	cAMP-dependent protein kinase catalytic subunit	405	46,7	2
TPM2	Putative tropomyosin isoform 2	161	19	22
TPS1	Trehalose-6-phosphate synthase	478	54,4	7
TPS2	Trehalose-6-phosphate (Tre6P) phosphatase	888	100,3	13
TPS3	Predicted trehalose-phosphate synthase regulatory subunit	1007	112,3	7
TRP2	Putative anthranilate synthase with a predicted role in tryptophan biosynthesis	522	58,9	15
TRP3	Putative bifunctional enzyme with predicted indole-3-glycerol-phosphate synthase and anthranilate synthase activities	520	58,3	3
TRP4	Predicted enzyme of amino acid biosynthesis	369	39,5	5
TRP5	Predicted tryptophan synthase	702	76	9
TRP99	Putative thioredoxin peroxidase/alkyl hydroperoxide reductase	184	20,1	3

TRR1	Thioredoxin reductase	320	34,7	6
TRX1	Thioredoxin	103	11,5	5
TSA1	TSA/alkyl hydroperoxide peroxidase C (AhPC) family protein	196	21,8	12
TSM1	Putative transcription initiation factor TFIID subunit	1425	161,8	2
TTR1	Putative glutaredoxin	119	13,1	5
TUB1	Alpha-tubulin	448	49,9	8
TUB2	Beta-tubulin	449	50	8
TUF1	Translation elongation factor TU	426	46,7	11
TUP1	Transcriptional corepressor	512	57,6	15
TYS1	Putative tRNA-Tyr synthetase	409	45	11
UAP1	UDP-N-acetylglucosamine pyrophosphorylase, catalyzes biosynthesis of UDP-N-acetylglucosamine from UTP and N-acetylglucosamine 1-phosphate	486	54,6	5
UBA1	Ubiquitin-activating enzyme	1021	114,2	32
UBI3	Fusion of ubiquitin with the S34 protein of the small ribosomal subunit	193	22,6	9
UBP6	Putative ubiquitin-specific protease of the 26S proteasome	478	54,1	6
UCF1	Upregulated by cAMP in filamentous growth	201	23,6	10
UGA1	Putative GABA transaminase	471	52,6	8
UGA2	Predicted succinate semialdehyde dehydrogenase	509	54,6	7
UGP1	UTP-glucose-1-phosphaturidyl transferase	500	55,5	21
URA2	Putative bifunctional carbamoylphosphate synthetase-aspartate transcarbamylase	2216	244,5	44
URA5	Putative orotate phosphoribosyltransferase	218	23,5	5
URA7	CTP synthase 1	573	64	5
USO6	Putative vesicular transport protein	1130	128,6	8
VAC8	Protein involved in vacuolar inheritance	585	63,4	2
VAS1	Putative tRNA-Val synthetase	1119	127,5	22
VID27	Protein similar to S. cerevisiae Vid27p	745	84,8	2
VMA10	Subunit G of the V1 peripheral membrane domain of the vacuolar H+-ATPase (V-ATPase)	113	12,8	8
VMA2	Vacuolar H(+)-ATPase	512	57,2	21
VMA4	H+ transporting ATPase E chain	226	25,4	11
VMA5	Putative vacuolar H(+)-ATPase	388	44,7	6
VMA8	Putative vacuolar H+-ATPase subunit	267	30	3
VPH1	Vacuolar H(+)-ATPase	821	93,3	3
VPS1	Dynamin-family GTPase-related protein	693	76,9	7
VPS21	Late endosomal Rab small monomeric GTPase involved in transport of endocytosed proteins to the vacuole	216	23,4	2
VPS35	Putative role in vacuolar sorting	944	109	2
VRP1	Verprolin-related protein involved in actin cytoskeleton organization and polarized morphogenesis	664	68,1	4
WRS1	Putative tRNA-Trp synthetase	424	48,1	9
XKS1	Putative xylulokinase	616	68,9	5
XYL2	D-xylulose reductase	360	38,7	15
YBN5	P-loop ATPase with similarity to human OLA1 and bacterial YchF	396	44,3	18
YCF1	Putative glutathione S-conjugate transporter	1580	177,6	4
YCP4	Putative flavodoxin	288	29,8	2
YDJ1	Putative type I HSP40 co-chaperone	393	42,7	3
YHB1	Nitric oxide dioxygenase	398	45,8	4
YHM1	Putative mitochondrial carrier protein	304	33,2	6
YHM2	Predicted carrier protein	301	32,8	5
YNK1	Nucleoside diphosphate kinase (NDP kinase)	151	16,9	7

YPT1	Functional homolog of S. cerevisiae Ypt1p, which is an essential small Ras-type GTPase involved in protein secretion at ER-to-Golgi	207	23	7
YRB1	Functional homolog of S. cerevisiae Yrb1p	212	24,2	6
YSA1	Predicted Nudix hydrolase family member with ADP-ribose pyrophosphatase activity	233	26,1	3
YST1	Ribosome-associated protein	261	28,7	9
ZPR1	Protein with putative zinc finger	509	57	10
ZUO1	Ortholog of S. cerevisiae Zuo1	427	48,3	12
ZWF1	Glucose-6-phosphate dehydrogenase	507	58,3	26

Table S2 b. Proteins identified in at least 2 replicates of HWCL (hyphal whole cell extract) with at least 2 peptides in a replicate

	Description	MW [kDa]	Unique Peptides
AAT1	Aspartate aminotransferase	48,9	24
AAT21	Putative aspartate aminotransferase	46	10
AAT22	Aspartate aminotransferase	46,8	3
ABP1	Ortholog of S. cerevisiae Abp1	70,6	32
ACB1	Protein similar to a region of acyl-coenzyme-A-binding protein	9,9	5
ACC1	Putative acetyl-coenzyme-A carboxylases	253,3	48
ACH1	Acetyl-coA hydrolase	57,9	24
ACO1	Aconitase	84,2	30
ACO2	Putative aconitate hydratase 2	85,9	8
ACS1	Acetyl-CoA synthetase	75,1	13
ACS2	Acetyl-CoA synthetase	74,3	27
ACT1	Actin	41,7	10
ADE1	Phosphoribosylaminoimadazole succinocarboxamide synthetase, enzyme of adenine biosynthesis	32,9	10
ADE12	Adenylosuccinate synthase	47,9	9
ADE13	Adenylosuccinate lyase	54,4	17
ADE17	5-Aminoimidazole-4-carboxamide ribotide transformylase, enzyme of adenine biosynthesis	64,9	23
ADE2	Phosphoribosylaminoimadazole carboxylase	62,4	7
ADE5,7	Phosphoribosylamine-glycine ligase and phosphoribosylformylglycinamidine cyclo-ligase	86	16
ADE6	5-Phosphoribosylformyl glycinamidine synthetase	150,7	15
ADH1	Alcohol dehydrogenase	36,8	19
ADH2	Alcohol dehydrogenase	36,8	13
ADH5	Putative alcohol dehydrogenase	35,7	6
ADK1	Putative adenylate kinase	27,6	14
ADO1	Adenosine kinase	38,2	11
AGM1	Phosphoacetylglucosamine mutase (N-acetylglucosamine-phosphate mutase)	60,4	10
AHA1	Putative Hsp90p co-chaperone	38,7	10
AHP1	Alkyl hydroperoxide reductase	19,2	5
AHP2	Putative thiol-specific peroxiredoxin	20	4
AIP2	Putative actin interacting protein	58,4	8
ALA1	Alanyl-tRNA synthetase	108,2	26
ALD5	NAD-aldehyde dehydrogenase	53,9	29
ALS3	Cell wall adhesin	123,7	2
ALT1	Putative alanine transaminase	58,4	4
AMS1	Putative alpha-mannosidase	131,5	34
ANB1	Translation initiation factor eIF-5A	17,1	9
APE2	Neutral arginine, alanine, leucine specific metallo-aminopeptidase	104,3	31
APE3	Putative vacuolar aminopeptidase Y,	60,8	6
APR1	Vacuolar aspartic proteinase	45,4	6
APT1	Adenine phosphoribosyltransferase	20,9	5
ARA1	D-Arabinose dehydrogenase	37,4	9
ARC1	Putative G4 nucleic acid binding protein	40,9	14
ARC15	Putative ARP2/3 complex subunit	17,7	5
ARC19	Putative ARP2/3 complex subunit	20,1	4

ARC35	Putative ARP2/3 complex subunit	40,5	2
ARC40	Protein similar to S. cerevisiae Arc40	42,3	4
ARD	D-arabitol dehydrogenase, NAD-dependent (ArDH)	30,7	6
ARF2	Putative ADP-ribosylation factor	20,6	9
ARG1	Argininosuccinate synthase	46,1	10
ARG3	Putative ornithine carbamoyltransferase	38,6	5
ARG4	Argininosuccinate lyase, catalyzes the final step in the arginine biosynthesis pathway	52,8	7
ARG5,6	Arginine biosynthetic enzyme	95,1	13
ARG8	Putative acetylornithine aminotransferase	50,1	4
ARO1	Putative pentafunctional arom enzyme	169,4	15
ARO2	Putative chorismate synthase	44,8	6
ARO3	3-deoxy-D-arabinoheptulosonate-7-phosphate synthase	40,6	8
ARO4	3-deoxy-D-arabinoheptulosonate-7-phosphate synthase	40,3	14
ARO8	Aromatic transaminase of the Ehrlich fusel oil pathway of aromatic alcohol biosynthesis	54,7	9
ARO9	Aromatic transaminase	58,8	6
ARP3	Protein with Myo5p-dependent localization to cortical actin patches at hyphal tip	46,6	6
ARP4	Subunit of the NuA4 histone acetyltransferase complex	52,8	3
ARP8	Putative chromatin-remodeling enzyme complex protein	121,3	4
ARP9	Protein similar to S. cerevisiae Arp3p, a component of the Arp2/3 complex involved in actin-dependent processes	54,6	4
ARX1	Putative ribosomal large subunit biogenesis protein	62,2	6
ASC1	40S ribosomal subunit similar to G-beta subunits	34,5	13
ASN1	Putative asparagine synthetase	64,6	13
ASR1	Heat shock protein	37,2	7
ASR3	Adenylyl cyclase and stress responsive protein	13	3
ATP1	ATP synthase alpha subunit	58,9	26
ATP18	F1F0 ATP synthase complex subunit	7,3	2
ATP2	F1 beta subunit of F1F0 ATPase complex	53,9	22
ATP20	Putative mitochondrial ATP synthase	12,6	4
ATP3	F1-ATP synthase complex subunit	32	8
ATP4	Putative F0-ATP synthase subunit 4	25,8	12
ATP5	Putative F0-ATP synthase FO subunit B	22,1	9
ATP7	Putative subunit of the F1F0-ATPase complex	19,4	5
AUT7	Putative autophagosome protein	15,6	3
BAT21	Putative branched chain amino acid aminotransferase	43,8	5
BAT22	Putative branched chain amino acid aminotransferase	40,8	12
BBC1	Putative SH3-domain-containing protein	104,5	3
BCY1	Protein kinase A regulatory subunit	50,2	13
BFR1	Protein involved in the maintenance of normal ploidy	53,5	17
BGL2	Cell wall 1,3-beta-glucosyltransferase	33,6	4
BMH1	Sole 14-3-3 protein in C. albicans	29,5	19
BRE1	Putative transcription factor with C3HC4 zinc finger DNA-binding motif	78,5	5
BUB3	Protein similar to S. cerevisiae Bub3	42,8	3
BUD7	Protein of unknown function	86,2	9
CAM1	Putative translation elongation factor eEF1 gamma	47	12
CAM1-1	Putative translation elongation factor	47,5	9
CAR2	Ornithine aminotransferase	47,3	12
CAT1	Catalase	54,8	9
CAT2	Major carnitine acetyl transferase	71,4	21
CBP1	Corticosteroid binding protein	55,5	6

CCS1	Copper chaperone involved in activation and protection of superoxide dismutase Sod1p	26,5	3
CCT2	Chaperonin of the cytosolic TCP1 ring complex	56,8	7
CCT3	Putative cytosolic chaperonin Cct ring complex subunit	58,6	10
CCT5	T-complex protein 1, epsilon subunit	59,8	8
CCT6	Putative cytosolic chaperonin Cct ring complex subunit	60,7	5
CCT7	Cytosolic chaperonin Cct ring complex	60,4	4
CCT8	Chaperonin-containing T-complex subunit	58,2	8
CDC10	Septin, required for wild-type cell, hyphal, or chlamydospore morphology	40,6	10
CDC11	Septin	46,7	5
CDC12	Septin	45,8	5
CDC19	Pyruvate kinase at yeast cell surface	55,4	30
CDC3	Septin	49	9
CDC37	Chaperone for Crk1p	58,5	4
CDC48	Putative microsomal ATPase	90,9	29
CDC60	Cytosolic leucyl tRNA synthetase	125,3	27
CEF3	Translation elongation factor 3	116,9	30
CGR1	Negative regulator of yeast-form growth	38,4	4
CHC1	Clathrin heavy chain	188,2	29
CHS5	Putative chitin biosynthesis protein	62,1	5
CIP1	Possible oxidoreductase	33	8
CIT1	Citrate synthase	52	26
CLC1	Clathrin light chain	26	9
CMD1	Calmodulin	16,5	8
CMK2	Putative calmodulin-dependent protein kinase	53,7	8
CMP1	Catalytic subunit of calcineurin (Ca[2+]-calmodulin-regulated S/T protein phosphatase)	69,6	6
COF1	Putative cofilin	15,8	7
COQ6	Protein of unknown function	53,2	6
COX2	Subunit II of cytochrome c oxidase, which is the terminal member of the mitochondrial inner membrane electron transport chain	29,8	2
COX5	Cytochrome oxidase subunit V	18,6	7
CPA1	Putative carbamoyl-phosphate synthase subunit	46,9	4
CPA2	Putative arginine-specific carbamoylphosphate synthetase	127	11
CPR3	Putative peptidyl-prolyl cis-trans isomerase	21	5
CPR6	Putative peptidyl-prolyl cis-trans isomerase	40,7	9
CPY1	Carboxypeptidase Y	61	3
CRG1	Methyltransferase involved in sphingolipid homeostasis, methylates a drug cantharidin	33,8	4
CRH11	GPI-anchored cell wall transglycosylase, putative ortholog of S. cerevisiae Crh1p	46,7	5
CRM1	Functional homolog of S. cerevisiae Crm1, which acts in protein nuclear export	124	17
CRN1	Coronin	70,6	11
CSH1	Aldo-keto reductase	38,2	10
CSP37	Hyphal cell wall protein	37	15
CTA3	Protein similar to <i>S. cerevisiae</i> Ede1p, which is involved in endocytosis	140,4	16
CTM1	Putative cytochrome c lysine methyltransferase	79	4
CTR1	Copper transporter	27,8	2
CUE5	Predicted ubiquitin-binding protein	48	7
CYC1	Cytochrome c	12,2	7
CYM1	Putative metalloprotease of the mitochondrial intermembrane space	117,5	3
CYP1	Peptidyl-prolyl cis-trans isomerase	17,6	11

CYP5	Putative peptidyl-prolyl cis-trans isomerase	22,4	7
CYS3	Cystathionine gamma-lyase	42,9	8
CYS4	Cystathionine beta-synthase	53,9	22
DAC1	N-acetylglucosamine-6-phosphate (GlcNAcP) deacetylase	45,2	4
DAK2	Putative dihydroxyacetone kinase	64,9	21
DDI1	Putative DNA damage inducible v-SNARE binding protein	35,9	8
DDR48	Immunogenic stress-associated protein	22,6	20
DED81	Putative tRNA-Asn synthetase	62,2	17
DOA1	WD repeat protein	84,3	9
DOS2	Protein of unknown function	41,5	2
DOT5	Putative nuclear thiol peroxidase	29,1	15
DPS1-1	Putative tRNA-Asp synthetase	65,3	16
DQD1	Putative 3-dehydroquinate dehydratase	16	3
DTD2	Protein of unknown function	17,9	3
DUT1	dUTP pyrophosphatase	16,9	4
EBP7	Putative NADPH oxidoreductase	43,8	2
ECM17	Putative sulfite reductase beta subunit	160	9
ECM29	Putative scaffold protein	208,6	6
ECM33	GPI-anchored cell wall protein	43,5	10
ECM4	Cytoplasmic glutathione S-transferase	39	15
ECM42	Ornithine acetyltransferase	46,8	2
EFB1	Translation elongation factor EF-1 beta	23,5	8
EFT2	Elongation Factor 2 (eEF2)	93,3	39
EGD1	Putative GAL4 DNA-binding enhancer protein	17	7
EGD2	Nascent polypeptide associated complex protein alpha subunit	19,5	9
EIF4E	Translation initiation factor eIF4E	24,2	11
END3	Regulated by Gcn4p	45	5
ENO1	Enolase	47,2	35
ERF1	Putative translation release factor 1, which interacts with stop codons and promotes release of nascent peptides from ribosomes	49,1	4
ERG10	Acetyl-CoA acetyltransferase	41,9	22
ERG12	Protein of unknown function	46,9	3
ERG13	3-hydroxy-3-methylglutaryl coenzyme A synthase	49,7	14
ERG20	Putative farnesyl pyrophosphate synthetase involved in isoprenoid and sterol biosynthesis, based on similarity to S. cerevisiae Erg20p	40,7	10
ERG8	Putative phosphomevalonate kinase	48,3	3
ERO1	Ortholog of S. cerevisiae Ero1	66,9	3
ESS1	Prolyl isomerase (parvulin class)	19,8	4
ETR1	Putative 2-enoyl thioester reductase	39,9	7
FAA4	Predicted acyl CoA synthase	77,3	9
FAS1	Beta subunit of fatty-acid synthase	227,7	67
FAS2	Alpha subunit of fatty-acid synthase	207,2	66
FBA1	Fructose-bisphosphate aldolase	39,2	20
FBP1	Fructose-1,6-bisphosphatase	35,9	7
FDH1	Formate dehydrogenase	41,8	14
FDH3	Glutathione-dependent formaldehyde dehydrogenase	40,6	9
FET34	Multicopper ferroxidase	70,7	5
FGR44	Protein lacking an ortholog in S. cerevisiae	46,8	4
FOX2	3-hydroxyacyl-CoA epimerase	99,3	23
FRS1	Phenylalanyl-tRNA synthetase	66,8	14
FRS2	Putative tRNA-Phe synthetase	56,4	15

FUM11	Fumarate hydratase	50	10
FUM12	Putative fumarate hydratase	55,4	14
FUR1	Uracil phosphoribosyltransferase	24,5	4
GAD1	Putative glutamate decarboxylase	63,9	17
GAL1	Galactokinase	57,1	13
GAL10	UDP-glucose 4-epimerase	75,4	26
GAL7	Putative galactose-1-phoshphate uridyl transferase	44,7	12
GBP2	Putative single-strand telomeric DNA-binding protein	49,2	8
GCD11	Gamma subunit of translation initiation factor eIF2	58,5	6
GCF1	HMG box mitochondrial protein	28,5	3
GCV1	Putative T subunit of glycine decarboxylase	43,7	5
GCV2	Glycine decarboxylase P subunit	109,8	8
GCV3	Glycine decarboxylase, subunit H	20	7
GCY1	Aldo/keto reductase	33	9
GDB1	Putative glycogen debranching enzyme	173,8	14
GDH2	Putative NAD-specific glutamate dehydrogenase	119,9	18
GDH3	NADP-glutamate dehydrogenase	49,5	26
GDI1	Putative Rab GDP-dissociation inhibitor	50,1	8
GFA1	Glucosamine-6-phosphate synthase, homotetrameric enzyme of chitin/hexosamine biosynthesis	79,2	5
GGA2	Protein involved in Golgi trafficking	64,7	4
GLC3	Putative 1,4-glucan branching enzyme	78,6	10
GLC7	Putative catalytic subunit of type 1 serine/threonine protein phosphatase	37,8	3
GLE2	Putative nuclear pore complex	42	5
GLG2	Putative self-glucosylating initiator of glycogen synthesis	42	5
GLK1	Putative glucokinase	52,1	18
GLN1	Putative glutamate synthase	41,7	8
GLN4	Putative tRNA-Gln synthetase	91,3	20
GLO1	Putative monomeric glyoxalase I	39,5	5
GLO2	Protein of unknown function	30,7	4
GLO3	Putative ARF GTPase activator	49,3	6
GLR1	Glutathione reductase	56,4	13
GLX3	Glutathione-independent glyoxalase	25,8	9
GNA1	Glucosamine-6-phosphate acetyltransferase	16,9	3
GND1	6-phosphogluconate dehydrogenase	54,3	30
GPD2	Surface protein similar to glycerol 3-P dehydrogenase	40,8	7
GPH1	Putative glycogen phosphorylase	102,8	31
GPM1	Phosphoglycerate mutase	27,4	19
GRE2	Putative reductase	38	3
GRE3	Putative D-xylose reductase	42,4	12
GRP2	Methylglyoxal reductase	37,6	20
GRS1	Putative tRNA-Gly synthetase	73,7	25
GRX3	Putative glutaredoxin	28,4	4
GSP1	Small RAN G-protein	24,3	8
GST2	Glutathione S transferase	25,2	7
GTT11	Glutathione S-transferase, localized to ER	28,6	6
GUA1	Putative GMP synthase, involved in the final step of guanine biosynthesis	58,8	7
GUK1	Putative guanylate kinase	21,3	6
GUS1	Putative glutamine-tRNA ligase	82,5	15
GVP36	BAR domain protein	36,1	8

HAL21	Putative phosphoadenosine-5'-phosphate or 3'-phosphoadenosine 5'-phosphosulfate phosphatase	36,8	3
HAL22	Putative phosphoadenosine-5'-phosphate or 3'-phosphoadenosine 5'-phosphosulfate phosphatase	38,7	4
HAP2	CCAAT-binding transcription factor	41	2
HAT1	Hat1-Hat2 histone acetyltransferase complex subunit	48,3	4
HAT2	Putative Hat1-Hat2 histone acetyltransferase complex subunit	42,8	3
HBR1	Essential protein involved in regulation of MTL gene expression	28,7	4
HBR2	Putative alanine glyoxylate aminotransferase	46,4	4
HCH1	Ortholog of S. cerevisiae Hch1, a regulator of heat shock protein Hsp90	17,7	3
HCR1	Putative translation initiation factor	32	3
HEM13	Coproporphyrinogen III oxidase	36,9	11
HEM2	Putative porphobilinogen synthase	37,1	6
HET1	Putative sphingolipid transfer protein	21,8	6
HEX1	Beta-N-acetylhexosaminidase/chitobiase, highly glycosylated enzyme that is secreted to the periplasm and culture medium	63,4	3
HHF1	Putative histone H4	11,6	4
HHO1	Putative histone H1	18,5	4
HIS1	ATP phosphoribosyl transferase	32,6	10
HIS4	Multifunctional enzyme that catalyzes three steps of histidine biosynthesis, with phosphoribosyl-AMP cyclohydrolase, phosphoribosyl-ATP diphosphatase, and histidinol dehydrogenase activities	91,9	12
HIS5	Putative histidinol-phosphate aminotransferase	43,1	5
HIS7	Putative imidazole glycerol phosphate synthase	69,6	6
HMT1	Major type I protein arginine methyltransferase (PRMT)	39,3	3
HNT1	Protein with similarity to protein kinase C inhibitor-I	17	2
HOG1	MAP kinase of osmotic-, heavy metal-, and core stress response	42,9	3
HOM2	Aspartate-semialdehyde dehydrogenase	39,2	11
HOM3	Putative L-aspartate 4-P-transferase	59,6	9
HOM6	Putative homoserine dehydrogenase	38,8	16
HPT1	Putative hypoxanthine-guanine phosphoribosyltransferase	24,2	5
HRR25	Predicted protein serine/threonine kinase	50,5	2
HRT2	Protein described as having a role in Ty3 transposition	52,7	6
HSE1	ESCRT-0 complex subunit	56,6	2
HSP104	Heat-shock protein	99,9	34
HSP12	Heat-shock protein	13,3	1
HSP21	Small heat shock protein	21,5	12
HSP60	Heat shock protein	60,1	37
HSP70	Putative hsp70 chaperone	70,3	23
HSP78	Heat-shock protein	91,8	22
HSP90	Essential chaperone, regulates several signal transduction pathways and temperature-induced morphogenesis	80,8	54
HTA1	Histone H2A	14	1
HTA2	Putative histone H2A	13,8	1
HTA3	Putative histone H2A	14,4	1
HTS1	Putative tRNA-His synthetase	55,6	9
HXK1	N-acetylglucosamine (GlcNAc) kinase	54,9	7
HXK2	Hexokinase II	53,4	32
ICL1	Isocitrate lyase	61,4	24
IDH1	Putative mitochondrial NAD-isocitrate dehydrogenase subunit 1	39,2	17
IDH2	Putative mitochondrial NAD-isocitrate dehydrogenase subunit	39,8	17
IDI1	Protein of unknown function	32,3	6

IDP1	Putative isocitrate dehydrogenase	48,5	20
IDP2	Isocitrate dehydrogenase	46,3	20
IFD6	Aldo-keto reductase	39,2	1
IFE2	Putative alcohol dehydrogenase	48,5	5
IFG3	Putative D-amino acid oxidase	38,7	9
IFR2	Zinc-binding dehydrogenase	38,5	10
IFU5	Predicted membrane protein	26	3
ILS1	Putative isoleucyl-tRNA synthetase, the target of drugs including the cyclic beta-amino acid icofungipen/PLD-118/BAY-10-8888 and mupirocin	125,2	30
ILV2	Putative acetolactate synthase	74,3	14
ILV3	Dihydroxyacid dehydratase	62,8	8
ILV5	Ketol-acid reductoisomerase	44,9	13
ILV6	Putative regulatory subunit of acetolacetate synthase	33,2	2
IMH3	Inosine monophosphate (IMP) dehydrogenase	56,2	14
INO1	Inositol-1-phosphate synthase	57,7	32
IPK2	Putative inositol polyphosphate multikinase	39,5	3
IPP1	Putative inorganic pyrophosphatase	32,1	14
IRO1	Putative transcription factor	65,3	4
IST2	Protein of unknown function	106,9	10
KAR2	Similar to Hsp70 family chaperones	74,5	38
KEL1	Kelch repeat domain-containing protein	113,9	4
KEX2	Subtilisin-like protease (proprotein convertase)	104,8	2
KGD1	Putative 2-oxoglutarate dehydrogenase	112	34
KGD2	Putative dihydrolipoamide S-succinyltransferase	48	11
KIS1	Snf1p complex scaffold protein	46,7	3
KRE5	UDP-glucose:glycoprotein glucosyltransferase	166,1	6
KRE9	Protein of beta-1,6-glucan biosynthesis	29,1	3
KRS1	Putative tRNA-Lys synthetase	67,7	17
LAP3	Putative aminopeptidase	58,2	7
LAP41	Putative aminopeptidase yscI precursor	56,7	13
LAT1	Putative dihydrolipoamide acetyltransferase component (E2) of pyruvate dehydrogenase complex	50	14
LEU1	3-isopropylmalate dehydratase	86	19
LEU2	Isopropyl malate dehydrogenase	40,1	11
LEU4	Putative 2-isopropylmalate synthase	64,2	8
LEU42	Putative alpha-isopropylmalate synthase	63,4	10
LHP1	Protein of unknown function	37,5	10
LHS1	Protein similar to S. cerevisiae Hsp70p	104,8	11
LIG1	tRNA ligase	95,5	5
LKH1	Putative leukotriene A(4) hydrolase	71,4	7
LPD1	Putative dihydrolipoamide dehydrogenase	52,8	10
LPG20	Aldo-keto reductase family protein	39,9	2
LSC1	Putative succinate-CoA ligase subunit	33,7	10
LSC2	Putative succinate-CoA ligase beta subunit	44,4	21
LSP1	Eisosome component with a predicted role in endocytosis	35,6	15
LYS1	Saccharopine dehydrogenase (biosynthetic)	42,4	10
LYS12	Homoisocitrate dehydrogenase	40,6	17
LYS2	Heterodimeric alpha-aminoadipate reductase large subunit	155,2	17
LYS21	Homocitrate synthase	47,5	5
LYS22	Putative homocitrate synthase	46,2	6
LYS4	Homoaconitase	74,4	10

LYS9	Saccharopine dehydrogenase	49,2	11
MAE1	Malic enzyme, mitochondrial	69,7	8
MAL2	Alpha-glucosidase	66,2	22
MAM33	Putative mitochondrial acidic matrix protein	29,9	6
MAS2	Putative processing peptidase, catalytic (alpha) subunit	58,2	3
MBF1	Putative transcriptional coactivator	16,3	10
MCR1	NADH-cytochrome-b5 reductase	33,4	12
MDG1	Protein of unknown function	70,5	21
MDH1	Mitochondrial malate dehydrogenase	36	14
MDH1-1	Predicted malate dehydrogenase precursor	34,7	18
MDH1-3	Predicted malate dehydrogenase	35,8	5
MES1	Cytoplasmic methionyl-tRNA synthetase	84,9	12
MET10	Sulfite reductase	121,9	7
MET13	Putative methionine biosynthesis protein	69,9	3
MET14	Putative adenylylsulfate kinase	22,5	5
MET15	O-acetylhomoserine O-acetylserine sulfhydrylase	48	23
MET3	ATP sulfurlyase	58,8	6
MET6	Essential 5-methyltetrahydropteroyltriglutamate-homocysteine methyltransferase (cobalamin-independent methionine synthase)	85,6	48
MEU1	Putative methylthioadenosine phosphorylase	37,6	6
MIA40	Predicted component of the mitochondrial intermembrane space import machinery	27,9	3
MIR1	Putative mitochondrial phosphate transporter	32,5	11
MIS11	Predicted mitochondrial C1-tetrahydrofolate synthase precursor	102,1	26
MIS12	Mitochondrial C1-tetrahydrofolate synthase precursor	111,2	8
MLC1	Microtubule-dependent localized protein	16	6
MLP1	Protein of unknown function	207,6	13
MLS1	Malate synthase	62,5	29
MMD1	Mitochondrial protein	17	5
MP65	Cell surface mannoprotein	39,2	4
MPRL36	Mitochondrial ribosomal protein of the large subunit	23,7	2
MRF1	Putative mitochondrial respiratory protein	38,6	8
MRP2	Protein similar to S. cerevisiae Mrp2p, which is a component of the small subunit of the mitochondrial ribosome	13,2	2
MRP20	Component of mitochondrial ribosome	34,3	4
MRP7	Mitochondrial ribosomal protein of the large subunit	44,5	5
MRP8	Mitochondrial ribosomal protein	23,4	3
MRPL27	Putative 60S ribosomal protein L27, mitochondrial precursor	19,3	4
MRPL33	Putative mitochondrial ribosomal protein of the large subunit	11,2	2
MRPL40	Putative mitochondrial ribosomal protein	38,9	3
MRPL8	Mitochondrial 60S ribosomal protein subunit	29,4	3
MSI3	Essential HSP70 family protein	78,5	33
MTR10	Putative importin	110,2	4
MVD	Mevalonate diphosphate decarboxylase	39,5	13
MXR1	Putative methionine sulfoxide reductase	21,1	6
NAG1	Glucosamine-6-phosphate deaminase	27,4	9
NAP1	Nucleosome assembly protein	49,5	4
NCE103	Carbonic anhydrase	31,6	3
NCP1	NADPH-cytochrome P450 reductase, acts with Erg11p in sterol 14 alpha-demethylation in ergosterol biosynthesis	76,6	8
NHP6A	Putative non-histone chromatin component	10,5	4
NIF3	Protein of unknown function	31	5

NIP1	Putative translation initiation factor	99,8	13
NIT3	Putative nitrilase	32,9	6
NMD3	Putative nonsense-mediated mRNA decay protein	59,2	5
NMT1	Myristoyl-CoA:protein N-myristoyltransferase	51,8	4
NOT5	Protein with similarity to S. cerevisiae Not5p, a member of the transcription regulatory CCR4-NOT complex	74,7	5
NPT1	Putative nicotinate phosphoribosyltransferase, involved in NAD salvage pathway	48,3	6
NSP1	Essential component of the nuclear pore complex	74,7	10
NTF2	Putative nuclear envelope protein	14,2	4
OFD1	Putative prolyl hydroxylase family member	70,8	6
OFR1	Protein of unknown function	15,4	4
orf19.1037	Protein of unknown function	68,8	4
orf19.1052	Predicted histone H2B	14,1	6
orf19.1058	Protein of unknown function	31,4	3
orf19.1085	Protein of unknown function	25,4	5
orf19.1086	Protein of unknown function	67	9
orf19.1107	Protein of unknown function	21,4	5
orf19.1152	Protein of unknown function	30,6	2
orf19.117	Protein of unknown function	58,7	4
orf19.1180	Putative 2-aminoadipate transaminase	46,5	2
orf19.1214	Protein of unknown function	50,3	3
orf19.1229	Protein of unknown function	119,1	7
orf19.1338	Protein of unknown function	41,1	4
orf19.1340	Putative aldose reductase	35,2	5
orf19.1355	Putative protein of unknown function	38,6	11
orf19.1374	Protein of unknown function	21,4	6
orf19.1389	Protein of unknown function	18,5	6
orf19.1394	Putative protein of unknown function	18,7	4
orf19.1409	Ribosomal 60S subunit protein L22B	14	3
orf19.1414	Protein of unknown function	31,6	3
orf19.1444	Protein of unknown function	60	6
orf19.1448	Protein of unknown function	10,3	3
orf19.1460	Putative glutamine-dependent NAD synthetase, involved in NAD salvage pathway	81,1	7
orf19.1544	Putative cis-golgi localized protein involved in ER to Golgi transport	29,8	6
orf19.1565	Protein of unknown function	69,5	2
orf19.1589	Protein of unknown function	51,2	3
orf19.1642	Ortholog of S. cerevisiae Loc1, a nuclear protein involved in asymmetric localization of ASH1 mRNA in S. cerevisiae	23,8	2
orf19.1662	Protein of unknown function	39,9	2
orf19.1697	Protein of unknown function	40,4	6
orf19.1709	Sterol carrier domain protein	13,8	4
orf19.1723	Protein of unknown function	24,2	2
orf19.1730	HMG-box protein	34,6	3
orf19.1777	Protein of unknown function	159,7	4
orf19.1785	Protein with a PI31 proteasome regulator domain	35,9	5
orf19.1796	Putative glyoxylate reductase	40,7	6
orf19.185	Protein of unknown function	41	6
orf19.1860	Protein of unknown function	53,7	4
orf19.1862	Possible stress protein	13,1	4
orf19.1889	Putative phosphoglycerate mutase family protein	27,2	3
orf19.1940	Protein of unknown function	27,5	3

orf19.1946	Similar to an aldose 1-epimerase-related protein	33,8	9
orf19.1967	Protein of unknown function	21	2
orf19.1993	Protein of unknown function	48,3	5
orf19.200	Putative nuclear RNA-binding protein	22	9
orf19.2036	Predicted dihydrodiol dehydrogenase	40,7	9
orf19.2065	Protein of unknown function	38,2	2
orf19.2114	Predicted uricase	34,4	9
orf19.2124	Predicted alcohol dehydrogenase	38,5	7
orf19.2125	Protein of unknown function	17,4	4
orf19.213	Protein of unknown function	32,1	5
orf19.2150	Putative ortholog of mammalian electron transfer flavoprotein complex subunit ETF-alpha	34,3	5
orf19.2175	Putative mitochondrial cell death effector	39,9	2
orf19.2214	Protein of unknown function	32,8	3
orf19.2244	Similar to oxidoreductases and to S. cerevisiae Yjr096wp	36,3	3
orf19.2262	Protein similar to quinone oxidoreductases	42,9	4
orf19.2263	Protein of unknown function	20,5	4
orf19.2269	Putative 3-phosphoserine phosphatase	27,7	6
orf19.2275	Putative mitochondrial ribosomal protein	18,7	6
orf19.2278	Putative 20S proteasome assembly protein	29,5	5
orf19.2286	Putative deoxyhypusine hydroxylase	35,8	5
orf19.2296	Predicted mucin-like protein	264,3	55
orf19.2304	Protein similar to S. cerevisiae Gvp36p	38,4	10
orf19.2305	Protein of unknown function	14,3	4
orf19.2333	Protein of unknown function	54,8	2
orf19.2335	Putative aspartyl aminopeptidase	55,1	4
orf19.2414	Ortholog of S. cerevisiae Mpm1	37,1	10
orf19.2478	60S ribosomal protein L7	27,4	14
orf19.2489	Putative karyopherin beta	122,1	11
orf19.2499	Putative peptidyl-prolyl cis-trans isomerase	42,5	2
orf19.2520	Protein of unknown function	37	3
orf19.2533	Protein with a predicted role in protein translocation from the endoplasmic reticulum	9,9	4
orf19.2639	Protein of unknown function	20,6	3
orf19.2710	Protein of unknown function	24,6	3
orf19.2720	Cytosolic chaperonin Cct ring complex subunit	59,3	7
orf19.2737	Carbohydrate kinase domain-containing protein	81,1	4
orf19.2755	Protein of unknown function	27,3	8
orf19.2769	Putative protease B inhibitor	13,6	5
orf19.2794	Putative non-specific single-domain racemase	31,2	3
orf19.2821	Protein of unknown function	19,9	6
orf19.2828	Protein of unknown function	32,6	4
orf19.285	Ortholog of S. cerevisiae Igo2	14,4	4
orf19.2930	Predicted translation initiation factor	71,5	9
orf19.3022	Protein of unknown function	35,8	3
orf19.3037	Putative poly(A)-binding protein	70,4	22
orf19.3053	Protein of unknown function	20,3	8
orf19.3141	Protein of unknown function	97,8	5
orf19.3235	Putative F-actin capping protein subunit alpha	32	4
orf19.3260	Protein of unknown function	80,3	2
orf19.3312	Protein of unknown function	71,4	9

orf19.3319	Putative thioredoxin	36,9	4
orf19.3335	Plasma membrane protein of unknown function	21,8	12
orf19.3341	Putative tRNA-Arg synthetase	70,8	18
orf19.3354	Protein of unknown function	29,4	18
orf19.3362	Protein of unknown function	50,3	2
orf19.3367	Protein of unknown function	34,3	4
orf19.338	Putative glycoside hydrolase	121,2	18
orf19.3442	Putative oxidoreductase	47	2
orf19.3475	Described as a Gag-related protein	19,6	14
orf19.3480	Protein of unknown function	54,6	5
orf19.3482	Protein of unknown function	52,9	4
orf19.3508	Putative protein of unknown function	42	6
orf19.3515	Putative 3-hydroxyanthranilic acid dioxygenase, involved in NAD biosynthesis	19,5	3
orf19.3559	S. cerevisiae ortholog Mrps35p is a structural constituent of ribosome and localizes to mitochondrial small ribosomal subunit	36,2	9
orf19.3572	Ribosomal 60S subunit protein L31B	13	11
orf19.3583	Protein of unknown function	43,1	6
orf19.36.1	Protein of unknown function	7,6	6
orf19.3649	Protein of unknown function	31,8	2
orf19.3681	Protein of unknown function	96,5	5
orf19.3690	Ribosomal 60S subunit protein	14,2	9
orf19.3755	Protein of unknown function	24,6	3
orf19.3797	Protein of unknown function	29,1	2
orf19.3799	Protein of unknown function	38,7	8
orf19.3915	Putative metallodipeptidase	47,9	10
orf19.3922	Possible pyrimidine 5' nucleotidase	33,2	2
orf19.3932	Predicted RNA binding protein	30,3	13
orf19.397	Protein of unknown function	17,2	4
orf19.3982	Maltase	67,8	29
orf19.4016	Putative ubiquinol-cytochrome-c reductase	47,9	9
orf19.4107	Exopolyphosphatase, hydrolyzes inorganic polyphosphate (poly P) into Pi residues	45,9	4
orf19.4149	Protein component of the small (40S) ribosomal subunit	17,6	9
orf19.4153	Protein of unknown function	60,9	3
orf19.4164	Protein of unknown function	27,2	3
orf19.4176	Protein of unknown function	28,9	4
orf19.4204	Protein of unknown function	35,2	6
orf19.4216	Putative heat shock protein	13,3	1
orf19.4220	Predicted pyridoxal 5'-phosphate synthase	20,8	2
orf19.4230	20S proteasome subunit (beta7)	31,5	4
orf19.4246	Protein with similarity to S. cerevisiae Ykr070w	66,3	13
orf19.4248	Ortholog of S. cerevisiae YLR118C (alias Apt1)	32,7	4
orf19.4278	Protein of unknown function	77,4	5
orf19.4306	Protein of unknown function	30,6	3
orf19.4340	Protein of unknown function	14,7	3
orf19.4373	Protein similar to S. cerevisiae Fmn1p, which is riboflavin kinase	20,1	3
orf19.4395	Putative actin cytoskeleton component	67,2	11
orf19.4476	Protein with a NADP-dependent oxidoreductase domain	39,1	2
orf19.449	Putative phosphatidyl synthase	44,8	5
orf19.4503	Similar to HMG-box variant of S. pombe	25,1	6
orf19.4530	Protein of unknown function	40,6	4

orf19.4532	Protein of unknown function	39,9	2
orf19.4570	Protein of unknown function	16,8	3
orf19.4609	Putative dienelactone hydrolase	26,7	9
orf19.4622	Protein of unknown function	41,2	5
orf19.4633	Protein of unknown function	29,1	6
orf19.4639	Protein present in exponential and stationary growth phase yeast cultures	25,2	5
orf19.4736	Protein of unknown function	60,7	2
orf19.4751	Protein of unknown function	34,2	5
orf19.477	Protein of unknown function	29,6	2
orf19.4796	Putative eIF-4E-binding repressor of CAP-dependent translation	20	5
orf19.4850	Protein of unknown function	70,7	2
orf19.4878	Protein of unknown function	93,3	6
orf19.4898	Putative protein of unknown function	24,3	6
orf19.4931	Putative tRNA-Cys synthetase	89,7	17
orf19.4932	Protein of unknown function	84,3	9
orf19.4947	Mitochondrial membrane protein of unknown function	18,6	3
orf19.4953	Putative ATPase	34,3	5
orf19.4963	Protein of unknown function	45,6	3
orf19.498	Putative mitochondrial ribosomal component of the small subunit	53,4	5
orf19.5054	Putative quinolinate phosphoribosyl transferase, involved in NAD biosynthesis	32,4	8
orf19.5095	Putative oxysterol-binding protein	148,3	6
orf19.511	Protein of unknown function	27,5	2
orf19.5126	Putative adhesin-like protein	43,2	7
orf19.5184	Putative Ran guanyl-nucleotide exchange factor	54,1	4
orf19.5194	Putative protein of unknown function	27,4	4
orf19.5201	Protein of unknown function	9,6	8
orf19.5235	Putative mitochondrial ribosomal protein of the large subunit	27,7	4
orf19.5239	Predicted alanine-tRNA ligase	52,2	3
orf19.5250	Protein of unknown function	17,9	3
orf19.5281	Predicted essential RNA-binding G protein	133,9	33
orf19.5293	Protein of unknown function	57,8	14
orf19.5345	Putative ubiquitin-like polyubiquitin-binding protein	35,9	3
orf19.5369	Uroporphyrinogen decarboxylase	40,6	5
orf19.5393	Putative cysteine sulfinate decarboxylase	55,6	4
orf19.5517	Similar to alcohol dehydrogenases	40,2	8
orf19.5525	Putative oxidoreductase	37,7	2
orf19.5553	Protein of unknown function	19,3	3
orf19.5597	Protein of unknown function	6,7	3
orf19.5619	Protein of unknown function	80,4	3
orf19.5620	Stationary phase enriched protein	33,8	6
orf19.5666	Protein of unknown function	16,6	5
orf19.5671	Protein of unknown function	90,1	7
orf19.5689	Epsilon-COP subunit of the coatomer	35,6	3
orf19.5747	Protein of unknown function	52	7
orf19.5763	Protein of unknown function	28,7	3
orf19.577	Predicted protein tyrosine phosphatase	74,1	4
orf19.5773	Putative dipeptidyl-peptidase III	79,7	19
orf19.581	Putative RNA-binding protein	75,4	6
orf19.5812	Ortholog of S. cerevisiae Ett1, a nuclear protein that inhibits replication of Brome mosaic virus	48,2	2

orf19.5833	Protein of unknown function	40,4	3
orf19.5834	Protein of unknown function	114,4	3
orf19.585	Protein of unknown function	33,2	3
orf19.590	Putative thiamine biosynthesis enzyme	33,6	6
orf19.5917	RNA binding protein required for export of poly(A)+ mRNA from the nucleus	24	5
orf19.5943	Protein of unknown function	29,1	16
orf19.5961	Protein of unknown function	27,3	5
orf19.6065	RNA polymerase II holoenzyme/mediator subunit	37,3	3
orf19.6136	Ortholog of S. cerevisiae Mrpl4, a mitochondrial ribosomal protein of the large subunit	36,4	3
orf19.6147	Putative histone chaperone	19,7	4
orf19.6160	Protein of unknown function	89,6	26
orf19.6170	Protein of unknown function	34,2	4
orf19.6220	Ribosomal 60S subunit protein	13,7	3
orf19.6250	Protein of unknown function	37,8	3
orf19.6358	Protein of unknown function	15,6	3
orf19.6415	Protein of unknown function	6,6	4
orf19.6423	Protein of unknown function	39,2	3
orf19.6424	Protein of unknown function	25,6	2
orf19.6453	Protein of unknown function	151,4	2
orf19.6477	Protein of unknown function	49,1	2
orf19.6507	Putative curved DNA-binding protein orthologous to S. pombe Cdb4	43,1	12
orf19.6554	Regulator of calcineurin	31,5	2
orf19.6559	RNA polymerase III transcription initiation factor complex (TFIIIC) subunit	54,3	11
orf19.6596	Putative esterase	34,2	9
orf19.6602	Protein of unknown function	33,3	2
orf19.6612	Putative mitochondrial protein	29,6	6
orf19.6625	Protein of unknown function	32,4	2
orf19.6665	Protein of unknown function	84,3	4
orf19.6693	Protein of unknown function	131,4	4
orf19.6701	Protein with similarity to amino acid-tRNA ligase	77,3	22
orf19.6731	Protein of unknown function	9,6	5
orf19.6739	Protein of unknown function	69,3	4
orf19.6809	Putative phosphomutase-like protein	37,3	11
orf19.6810	Protein of unknown function	73,1	10
orf19.6843	Protein of unknown function	42,1	5
orf19.6853	Protein of unknown function	39,3	6
orf19.6867	Protein with a predicted cytochrome b5-like heme/steroid binding domain	14,9	4
orf19.6882	Ribosomal 60S subunit protein	12,1	2
orf19.6883	Putative oxysterol binding protein family	48,2	4
orf19.6893	Protein of unknown function	51,6	4
orf19.6898	Protein of unknown function	10,4	2
orf19.7012	Protein of unknown function	20,1	2
orf19.7020	Protein similar to S. cerevisiae Kex1p, which is a pheromone-processing peptidase	78,7	3
orf19.7085	Protein of unknown function	50,4	5
orf19.7097	Putative cytoplasmic RNA-binding protein	20,5	4
orf19.7109	Protein of unknown function	19,8	4
orf19.7116	Protein of unknown function	37,2	4
orf19.713	Protein of unknown function	15,3	4
orf19.7140	Putative catechol o-methyltransferase	28,2	5

orf19.7152	Protein similar to Aspergillus CYSK O-acetylserine sulfhydrylase, suggesting that C. albicans uses an O-acetyl-serine (OAS) pathway of sulfur assimilation	39,3	7
orf19.7160	Protein of unknown function	23,8	2
orf19.7179	Protein of unknown function	70,5	6
orf19.7196	Putative vacuolar protease	52,1	6
orf19.7210	Protein of unknown function	37,4	2
orf19.7214	Glucan 1,3-beta-glucosidase	56,9	17
orf19.7215.3	Protein of unknown function	11,5	7
orf19.7234	Putative RSC chromatin remodeling complex component	64,3	5
orf19.7244	Putative fumarylacetoacetate hydrolase	25,7	4
orf19.7250	Protein of unknown function	16,8	4
orf19.7263	Putative X-Pro aminopeptidase	51,3	3
orf19.7264	Metalloprotease subunit of the 19S regulatory particle of the 26S proteasome lid	34,9	4
orf19.7269	Putative polyamine acetyltransferase	24,8	7
orf19.7297	Putative cystathionine gamma-synthase	42,4	9
orf19.7306	Aldo-keto reductase	39	13
orf19.7322	Protein of unknown function	17,2	4
orf19.7326	Protein of unknown function	67,2	3
orf19.7328	Protein with a Staphylococcal nuclease domain	100	11
orf19.7345	Protein of unknown function	39	3
orf19.7357	Protein of unknown function	40,1	6
orf19.7368	Protein of unknown function	56,6	2
orf19.7437	Putative protein of unknown function	34,6	5
orf19.7485	Putative mitochondrial ribosomal protein of the large subunit	32	3
orf19.7499	Putative nicotinic acid mononucleotide adenylyltransferase, involved in NAD salvage pathway	45,1	3
orf19.7531	Protein of unknown function	25,2	8
orf19.7675	Mitochondrial ribosomal protein of the large subunit	18,5	2
orf19.86	Putative glutathione peroxidase	18,1	7
orf19.863	Protein of unknown function	43,9	4
orf19.92	Protein with a predicted thioredoxin-like domain	99,9	28
orf19.94	Protein of unknown function	26,4	6
orf19.951	Protein of unknown function	18,3	2
orf19.969	Protein of unknown function	44,4	5
OSH3	Protein required for wild-type filamentation	95,2	3
OSM1	Putative flavoprotein subunit of fumarate reductase	54,3	18
OSM2	Putative mitochondrial fumarate reductase	70,4	8
OYE23	Putative NAPDH dehydrogenase	46	1
OYE32	NAD(P)H oxidoreductase family protein	47,5	8
PAN1	Essential protein involved in endocytosis and polarized growth	152,4	11
PAN6	Protein of unknown function	35,3	3
PCK1	Phosphoenolpyruvate carboxykinase	60,9	9
PDA1	Putative pyruvate dehydrogenase alpha chain	44,1	21
PDB1	Putative pyruvate dehydrogenase	41,3	13
PDC11	Pyruvate decarboxylase	62,4	24
PDI1	Putative protein disulfide-isomerase	63	28
PDX1	Pyruvate dehydrogenase complex protein X	46	12
PDX3	Pyridoxamine-phosphate oxidase	31,3	3
PEP8	Protein similar to S. cerevisiae Pep8p, which is involved in retrograde transport	39,4	2
PET9	Mitochondrial ADP/ATP carrier protein involved in ATP biosynthesis	32,7	15

PFK1	Phosphofructokinase alpha subinit	108,5	32
PFK2	Phosphofructokinase beta subunit	104,1	17
PFY1	Profilin	13,8	5
PGA4	GPI-anchored cell surface protein	49	6
PGA45	Putative GPI-anchored cell wall protein	48,9	3
PGA52	GPI-anchored cell surface protein of unknown function	41,4	3
PGA63	Component COPII vesicle coat	136,3	23
PGI1	Glucose-6-phosphate isomerase	61,1	28
PGK1	Phosphoglycerate kinase	45,2	40
PGM2	Ortholog of S. cerevisiae Pgm2	61,8	14
PHA2	Putative prephenate dehydratase	34,7	2
PHHB	Putative 4a-hydroxytetrahydrobiopterin dehydratase	13,2	2
PHO15	4-nitrophenyl phosphatase, possible histone H2A phosphatase	34,2	4
PHR1	Cell surface glycosidase	59,5	10
PHR2	Glycosidase	58,7	5
PIL1	Eisosome component	34,6	14
PIN3	Putative SH3-domain-containing protein	31	5
PLB3	GPI-anchored cell surface phospholipase B	69,7	3
PMA1	Plasma membrane H(+)-ATPase	97,6	23
PMI1	Phosphomannose isomerase	48,8	13
PMM1	Phosphomannomutase	29	19
PNG2	Putative peptide:N-glycanase	111,8	11
PNP1	Purine nucleoside phosphorylase	33,4	5
POL30	Similar to proliferating cell nuclear antigen (PCNA)	29	4
POR1	Mitochondrial outer membrane porin	29,7	16
POX1-3	Predicted acyl-CoA oxidase	78,9	16
PPH21	Putative protein phosphatase of the Type 2A-related family (serine/threonine-specific), similar to <i>S. cerevisiae</i> Pph21p	40,9	2
PR26	Protein with similarity to proteasomal 26S regulatory subunit of S. cerevisiae, H. sapiens, Methanobacterium thermoautotrophicum (Archaebacterium)	45,9	5
PRA1	Cell surface protein that sequesters zinc from host tissue	33,1	3
PRD1	Putative proteinase	81,7	11
PRE1	Putative beta 4 subunit of the 20S proteasome	22	7
PRE2	Putative proteasome beta-5 subunit	31,3	5
PRE3	Putative beta-1 proteasome subunit	23,3	6
PRE5	Alpha6 subunit of the 20S proteasome	31,4	11
PRE6	Putative alpha-4 subunit of the proteasome	27,4	8
PRE8	Putative alpha-2_sc subunit of proteasome	27,7	6
PRE9	Alpha3 (C9) subunit of the 20S proteasome	27,8	10
PRO2	Putative gamma-glutamyl phosphate reductase with a predicted role in proline biosynthesis	49,1	8
PRO3	Delta 1-pyrroline-5-carboxylate reductase	29	3
PRT1	Putative translation initiation factor eIF3	84,2	7
PRX1	Thioredoxin peroxidase	27,4	18
PSA2	Mannose-1-phosphate guanyltransferase	50,8	9
PST1	Putative 1,4-benzoquinone reductase	21,1	3
PST2	Putative NADH:quinone oxidoreductase	21,7	2
PST3	Putative flavodoxin	21,2	6
PTC2	Protein phosphatase of the Type 2C-related family (serine/threonine-specific) with a potential role in DNA damage checkpoint control	64,5	6
PTC7	Protein phosphatase, type 2C	38	3
PUP1	Putative beta 2 subunit of the 20S proteasome	29,4	3

PUP2	Alpha5 subunit of the 20S proteasome	27,7	7
PUP3	Putative beta 3 subunit of the 20S proteasome	22,6	5
PUT2	Putative delta-1-pyrroline-5-carboxylate dehydrogenase	65,7	11
PWP1	Putative rRNA processing protein	67,9	3
PYC2	Putative pyruvate carboxylase	129,7	46
QCR2	Ubiquinol-cytochrome-c reductase	39,5	15
RAD23	S. cerevisiae Rad23 ortholog	43,8	2
RAS1	RAS signal transduction GTPase	32,5	6
RBP1	Peptidyl-prolyl cis-trans isomerase	13,3	3
RCT1	Fluconazole-induced protein	20,5	9
RDI1	Putative rho GDP dissociation inhibitor	22,9	15
REG1	Putative protein phosphatase regulatory subunit	110,7	4
RET2	Delta subunit of the coatomer complex (COPI)	62,8	5
RFA2	Putative DNA replication factor A	29,4	3
RHO1	Small GTPase of Rho family	22	4
RIB4	Lumazine synthase (6,7-dimethyl-8-ribityllumazine synthase, DMRL synthase)	23,1	8
RIB5	Putative riboflavin synthase	25,7	8
RIM1	Putative single-stranded DNA-binding protein	16,2	9
RIP1	Putative ubiquinol cytochrome c-reductase	23,2	3
RKI1	Protein of unknown function	26	3
RNA1	Putative GTPase-activating protein	46	10
RNR21	Ribonucleoside-diphosphate reductase	47,4	7
RPL10	Ribosomal protein L10	25,2	12
RPL10A	Predicted ribosomal protein	24,4	11
RPL11	Ribosomal protein	19,8	8
RPL12	Ribosomal protein L12, 60S ribosomal subunit	17,8	7
RPL13	Putative ribosomal subunit	23	12
RPL14	Ribosomal protein L14	14,7	10
RPL15A	Putative ribosomal protein	24,3	7
RPL16A	Ribosomal protein	22,6	12
RPL17B	Ribosomal protein L17	21	8
RPL18	Predicted ribosomal protein	20,8	9
RPL19A	Ribosomal protein L19	21,9	12
RPL2	Putative 60S ribosomal protein L2	27,3	11
RPL20B	Ribosomal protein L20	20,3	14
RPL21A	Putative ribosomal protein	18	7
RPL23A	Ribosomal protein	14,5	7
RPL24A	Predicted ribosomal protein	17,4	10
RPL25	Putative rRNA-binding ribosomal protein component of the 60S ribosomal subunit	15,8	7
RPL27A	Ribosomal protein L27	15,5	8
RPL28	Putative ribosomal protein	16,7	9
RPL3	Ribosomal protein, large subunit	43,9	22
RPL30	Ribosomal 60S subunit protein	11,5	5
RPL32	Component of the large (60S) ribosomal subunit	14,9	8
RPL35	Ribosomal protein	14,1	10
RPL37B	Ribosomal protein L37	10,1	4
RPL38	60S ribosomal ribosomal protein subunit	8,9	4
RPL39	Ribosomal protein L39	11,1	4
RPL42	Putative 60S ribosomal subunit protein	12,2	3
RPL43A	Putative ribosomal protein, large subunit	10,1	2

RPL4B	Ribosomal protein 4B	39,2	14
RPL5	Ribosomal protein	34,5	7
RPL6	Ortholog of S. cerevisiae ribosomal subunit, Rpl6B	19,8	13
RPL7	Ribosomal protein L7	33,9	2
RPL82	Predicted ribosomal protein	28,2	1
RPL8B	Predicted ribosomal protein	28,5	4
RPL9B	Ribosomal protein L9	21,7	10
RPN1	Putative 19S regulatory particle of the 26S proteasome	109,9	18
RPN10	Putative 19S regulatory particle of the 26S proteasome	30,4	4
RPN2	Putative 26S proteasome subunit	104,7	13
RPN3	Putative non-ATPase regulatory subunit of the 26S proteasome lid	54,9	7
RPN6	Putative 26S proteasome subunit	49,4	7
RPN7	Subunit of the proteasome regulatory particle	46,2	4
RPN8	Putative regulatory subunit of the 26S proteasome	36,7	6
RPP0	Putative ribosomal protein	33,3	13
RPP1B	Conserved acidic ribosomal protein, likely involved in regulation of translation elongation	10,8	2
RPP2A	Acidic ribosomal protein	10,9	5
RPS1	Putative ribosomal protein 10 of the 40S subunit	29	16
RPS10	Ribosomal protein S10	13,8	5
RPS12	Acidic ribosomal protein S12	15,7	3
RPS13	Putative ribosomal protein of the small subunit	16,9	9
RPS14B	Putative ribosomal protein	14	11
RPS15	Putative ribosomal protein	15,9	5
RPS16A	Putative 40S ribosomal subunit	15,7	15
RPS17B	Ribosomal protein 17B	15,7	5
RPS18	Predicted ribosomal protein	17	12
RPS19A	Putative ribosomal protein S19	16,1	12
RPS20	Putative ribosomal protein	13,3	11
RPS21	Protein component of the small (40S) subunit	26,9	11
RPS21B	Ribosomal protein S21	9,6	7
RPS22A	Predicted ribosomal protein	14,8	6
RPS23A	Putative ribosomal protein	16	4
RPS24	Predicted ribosomal protein	15,5	10
RPS25B	Ribosomal protein	11,6	6
RPS26A	Ribosomal protein	13,6	5
RPS27	Putative ribosomal protein	9	3
RPS3	Ribosomal protein S3	27,3	17
RPS30	Putative 40S ribosomal protein S30	7,1	3
RPS5	Ribosomal protein S5	25,3	11
RPS6A	Ribosomal protein 6A	27,1	11
RPS7A	Ribosomal protein S7	21,2	13
RPS8A	Small 40S ribosomal subunit protein	22,7	11
RPS9B	Predicted ribosomal protein	21,7	12
RPT1	Putative 26S proteasome regulatory subunit 7	49,8	5
RPT2	Putative ATPase of the 19S regulatory particle of the 26S proteasome	51,8	6
RPT6	Putative ATPase of the 19S regulatory particle of the 26S proteasome	44,9	7
RSR1	RAS-related protein	27,6	3
SAC6	Fimbrin	72,1	8
SAH1	S-adenosyl-L-homocysteine hydrolase	49	20

SAM2	S-adenosylmethionine synthetase	42,2	17
SAP9	Secreted aspartyl protease	58,5	3
SAR1	Functional homolog of S. cerevisiae Sar1	21,5	5
SBA1	Similar to co-chaperones	23,9	12
SBP1	Similar to RNA binding proteins	31,9	15
SCL1	Proteasome subunit YC7alpha	27,4	8
SDH12	Succinate dehydrogenase	70,1	10
SDH2	Succinate dehydrogenase, Fe-S subunit	30,1	8
SDS22	Putative protein serine-threonine phosphatase	43	7
SEC13	Putative protein transport factor	33	5
SEC14	Essential protein	34,7	6
SEC21	Protein of unknown function	104,2	9
SEC23	Putative GTPase-activating protein	85,6	8
SEC24	Protein with a possible role in ER to Golgi transport	102	11
SEC26	Secretory vesicles coatomer complex protein	107,1	7
SEC27	Protein of unknown function	106	11
SEC4	Small GTPase of Rab family	23,1	3
SEC7	Putative guanine nucleotide exchange factor (GEF)	208,9	4
SER1	Putative 3-phosphoserine aminotransferase	43	8
SER33	Predicted enzyme of amino acid biosynthesis	50,3	17
SES1	Seryl-tRNA synthetase	52,6	16
SGT1	Putative co-chaperone protein with a predicted role in kinetochore assembly	47,3	6
SGT2	Putative small tetratricopeptide repeat (TPR)-containing protein	36,4	11
SHM1	Mitochondrial serine hydroxymethyltransferase	54,4	17
SHM2	Cytoplasmic serine hydroxymethyltransferase	52	15
SHP1	Regulator of the type 1 protein phosphatase Glc7p activity, involved in control of morphogenesis, progression through the cell cycle and response to DNA damage	41,4	15
SIS1	Putative Type II HSP40 co-chaperone	36,8	9
SKP1	Putative subunit D of kinetochore protein complex CBF3	19	5
SLA2	Actin binding protein with roles in growth control and morphogenesis	120,8	15
SLK19	Alkaline-induced protein of plasma membrane	133,9	39
SMI1	Cell wall biosynthesis protein	68,9	5
SMM1	Putative dihydrouridine synthase	49,2	2
SMT3	SUMO, small ubiquitin-like protein	11,1	6
SNF7	ESCRT III complex protein	25,9	5
SNO1	Protein with a predicted role in pyridoxine metabolism	28,4	2
SNZ1	Stationary phase protein	31,8	10
SOD1	Cytosolic copper- and zinc-containing superoxide dismutase	16,1	8
SOD2	Mitochondrial Mn-containing superoxide dismutase	26,2	5
SOD3	Cytosolic manganese-containing superoxide dismutase	22,7	5
SOD5	Cu and Zn-containing superoxide dismutase	23,6	5
SOL3	Putative 6-phosphogluconolactonase	28,5	14
SPE3	Putative spermidine synthase	33,9	6
SRB1	Essential GDP-mannose pyrophosphorylase	40	15
SRV2	Adenylate cyclase-associated protein	59,6	12
SSA2	HSP70 family chaperone	70	20
SSB1	HSP70 family heat shock protein	66,4	27
SSC1	Heat shock protein	69,7	41
SSD1	Protein with role in resistance to host antimicrobial peptides	141,2	17
SSZ1	Putative HSP70 chaperone	58,5	12

STF2	Protein involved in ATP biosynthesis	10,5	6
STI1	Protein that interacts with Cdc37 and Crk1 in two-hybrid	66	36
STR2	Protein of unknown function	67,1	3
SUB2	Putative TREX complex component with a predicted role in nuclear mRNA export	49,1	10
SUI1	Putative translation initiation factor	12,3	3
SUI2	Translation initiation factor eIF2, alpha chain	33,9	5
SUP35	Translation factor eRF3	79,9	10
SVF1	Putative survival factor	43	4
SYS3	Protein similar to S. cerevisiae Sys3p	102,8	6
TAF14	Putative DNA-binding transcription factor	29,1	3
TAL1	Transaldolase	35,7	25
TCP1	Chaperonin-containing T-complex subunit, induced by alpha pheromone in SpiderM medium	60	8
TDH3	NAD-linked glyceraldehyde-3-phosphate dehydrogenase	35,8	31
TEF2	Translation elongation factor 1-alpha	50	17
TFP1	Subunit of vacuolar H+-ATPase	67,6	19
TFS1	Putative carboxypeptidase y inhibitor	29,3	8
THR4	Putative threonine synthase	57,7	12
THS1	Putative threonyl-tRNA synthetase	81,6	17
TIF	Translation initiation factor	44,6	20
TIF11	Translation initiation factor eIF1a	17,4	2
TIF3	Putative translation initiation factor	50,7	8
TIF34	Putative translation initiation factor eIF3, p39 subunit	38,2	8
TIF4631	Putative translation initiation factor eIF4G	118,2	16
TIF5	Putative translation initiation factor	46,6	8
TIM10	Predicted protein of the mitochondrial intermembrane space with role in protein import into mitochondrial inner membrane	10,1	4
TIP120	Protein similar to human CAND1 (Cullin-Associated Nedd8-Dissociated) protein involved in regulation of SCF complexes	134,4	8
TKL1	Putative transketolase	73,7	27
TMA19	Cell wall protein, ortholog of S. cerevisiae Tma19p (Ykl065cp)	18,5	8
TOM1	Putative E3 ubiquitin ligase	375,3	4
TPI1	Triose-phosphate isomerase	26,6	14
TPM2	Putative tropomyosin isoform 2	19	22
TPS1	Trehalose-6-phosphate synthase	54,4	7
TPS2	Trehalose-6-phosphate (Tre6P) phosphatase	100,3	13
TPS3	Predicted trehalose-phosphate synthase regulatory subunit	112,3	7
TRP2	Putative anthranilate synthase with a predicted role in tryptophan biosynthesis	58,9	15
TRP3	Putative bifunctional enzyme with predicted indole-3-glycerol-phosphate synthase and anthranilate synthase activities	58,3	3
TRP4	Predicted enzyme of amino acid biosynthesis	39,5	5
TRP5	Predicted tryptophan synthase	76	9
TRP99	Putative thioredoxin peroxidase/alkyl hydroperoxide reductase	20,1	3
TRR1	Thioredoxin reductase	34,7	6
TRX1	Thioredoxin	11,5	5
TSA1	TSA/alkyl hydroperoxide peroxidase C (AhPC) family protein	21,8	12
TSR1	Component of 20S pre-rRNA processing unit	94,4	2
TTR1	Putative glutaredoxin	13,1	5
TUB1	Alpha-tubulin	49,9	8
TUB2	Beta-tubulin	50	8
TUF1	Translation elongation factor TU	46,7	11

TUP1	Transcriptional corepressor	57,6	15
TYS1	Putative tRNA-Tyr synthetase	45	11
UAP1	UDP-N-acetylglucosamine pyrophosphorylase, catalyzes biosynthesis of UDP-N-acetylglucosamine from UTP and N-acetylglucosamine 1-phosphate	54,6	5
UBA1	Ubiquitin-activating enzyme	114,2	32
UBI3	Fusion of ubiquitin with the S34 protein of the small ribosomal subunit	22,6	9
UBP6	Putative ubiquitin-specific protease of the 26S proteasome	54,1	6
UCF1	Upregulated by cAMP in filamentous growth	23,6	10
UGA1	Putative GABA transaminase	52,6	8
UGA2	Predicted succinate semialdehyde dehydrogenase	54,6	7
UGP1	UTP-glucose-1-phosphaturidyl transferase	55,5	21
URA2	Putative bifunctional carbamoylphosphate synthetase-aspartate transcarbamylase	244,5	44
URA4	Dihydroorotase	39,9	3
URA5	Putative orotate phosphoribosyltransferase	23,5	5
URA7	CTP synthase 1	64	5
USO6	Putative vesicular transport protein	128,6	8
UTR2	Putative GPI anchored cell wall glycosidase	51,7	3
VAS1	Putative tRNA-Val synthetase	127,5	22
VMA10	Subunit G of the V1 peripheral membrane domain of the vacuolar H+-ATPase (V-ATPase)	12,8	8
VMA2	Vacuolar H(+)-ATPase	57,2	21
VMA4	H+ transporting ATPase E chain	25,4	11
VPS1	Dynamin-family GTPase-related protein	76,9	7
VPS35	Putative role in vacuolar sorting	109	2
WRS1	Putative tRNA-Trp synthetase	48,1	9
XKS1	Putative xylulokinase	68,9	5
XYL2	D-xylulose reductase	38,7	15
YBN5	P-loop ATPase with similarity to human OLA1 and bacterial YchF	44,3	18
YCP4	Putative flavodoxin	29,8	2
YDJ1	Putative type I HSP40 co-chaperone	42,7	3
YHB1	Nitric oxide dioxygenase	45,8	4
YML6	Putative mitochondrial ribosomal protein	31,7	2
YNK1	Nucleoside diphosphate kinase (NDP kinase)	16,9	7
YPT1	Functional homolog of S. cerevisiae Ypt1p, which is an essential small Ras-type GTPase involved in protein secretion at ER-to-Golgi	23	7
YRB1	Functional homolog of S. cerevisiae Yrb1p	24,2	6
YSA1	Predicted Nudix hydrolase family member with ADP-ribose pyrophosphatase activity	26,1	3
YST1	Ribosome-associated protein	28,7	9
ZPR1	Protein with putative zinc finger	57	10
ZUO1	Ortholog of S. cerevisiae Zuo1	48,3	12
ZWF1	Glucose-6-phosphate dehydrogenase	58,3	26
